# Innovative Bioplasticizers from Residual **Cynara cardunculus** L. Biomass-Derived Levulinic Acid and Their Environmental Impact Assessment by LCA Methodology

**DOI:** 10.1021/acssuschemeng.3c02269

**Published:** 2023-08-01

**Authors:** Chiara Ruini, Paolo Neri, Gianluca Cavalaglio, Valentina Coccia, Franco Cotana, Anna Maria Raspolli Galletti, Davide Morselli, Paola Fabbri, Anna Maria Ferrari, Roberto Rosa

**Affiliations:** † Dipartimento di Scienze e Metodi dell’Ingegneria, Università degli Studi di Modena e Reggio Emilia, via G. Amendola 2, Reggio Emilia 42122, Italy; ‡ Università Telematica Pegaso, Centro Direzionale Isola f2, Napoli 80143, Italy; § Centro Interuniversitario di Ricerca sull’Inquinamento e sull’Ambiente “Mauro Felli”, Centro di Ricerca sulle Biomasse, University of Perugia, via G. Duranti 63, Perugia 06125, Italy; ∥ Dipartimento di Chimica e Chimica Industriale, Università di Pisa, via G. Moruzzi 13, Pisa 56124, Italy; ⊥ Dipartimento di Ingegneria Civile, Chimica, Ambientale e dei Materiali, Università di Bologna, via U. Terracini 28, Bologna 40131, Italy; # Consorzio Interuniversitario Nazionale per Scienza e Tecnologia dei Materiali (INSTM), via Giusti 9, Firenze 50121, Italy; ∇ Centro Interdipartimentale En&Tech, Università degli Studi di Modena e Reggio Emilia, Tecnopolo di Reggio Emilia, Piazzale Europa 1, Reggio Emilia 42123, Italy

**Keywords:** life cycle assessment, biomass valorization, plasticizers, bioplastics, environmental sustainability assessment, ReCiPe 2016

## Abstract

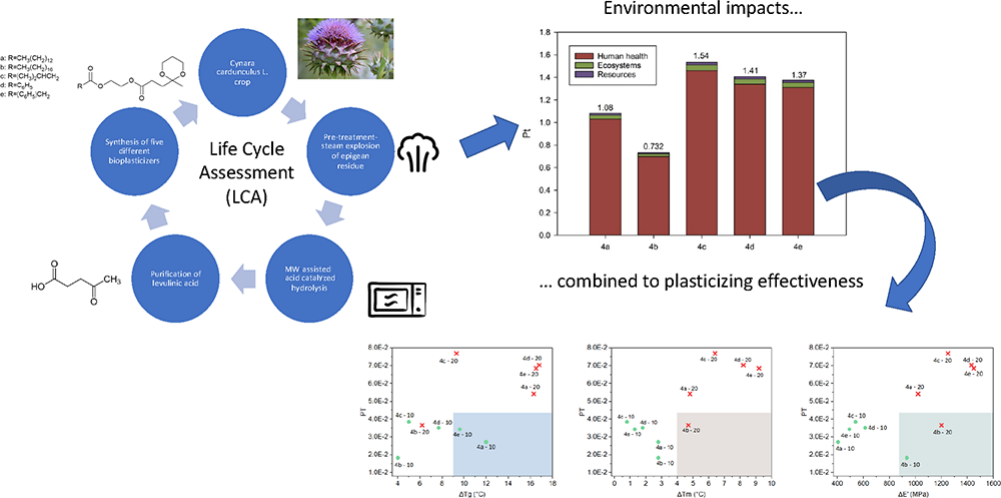

This work is focused on the application of Life Cycle Assessment (LCA) methodology for the quantification of the potential environmental impacts associated with the obtainment of levulinic acid from residual **Cynara cardunculus** L. biomass and its subsequent valorization in innovative bioplasticizers for tuning the properties as well as the processability of biopolymers. This potentially allows the production of fully biobased and biodegradable bioplastic formulations, thus addressing the issues related to the fossil origin and nonbiodegradability of conventional additives, such as phthalates. Steam explosion pretreatment was applied to the epigean residue of *C. cardunculus* L. followed by a microwave-assisted acid-catalyzed hydrolysis. After purification, the as-obtained levulinic acid was used to synthesize different ketal-diester derivatives through a three-step selective synthesis. The levulinic acid–base additives demonstrated remarkable plasticizing efficiency when added to biobased plastics. The LCA results were used in conjunction with those from the experimental activities to find the optimal compromise between environmental impacts and mechanical and thermal properties, induced by the bioadditives in poly(3-hydroxybutyrate), PHB biopolymer.

## Abstract 2

This work compares five bioplasticizers obtained from biomass-derived levulinic acid in terms of their environmental impacts together with PHB-induced thermal and mechanical properties.

## Introduction

In addition to their manufacturing phase, significant environmental burdens are also associated with fossil-based plastic materials because of their end of life since they are typically not biodegradable.^1^ Indeed, nowadays, plastic materials flow is mostly linear with less than 10% of globally generated plastic being recycled, approximately 40% being landfilled, *ca.* 14% being burned for energy recovery, and more than 30% being directly leaked into the environment.^2^

Therefore, biobased biodegradable plastics surely represent the most valuable alternative strategy to concurrently mitigate both the above-mentioned environmental issues.^3,4^ Moreover, the use of renewable resources, particularly nonedible biowastes (i.e., those avoiding any kind of conflict with the food chain) could also contribute to address further environmental issues, like for example the huge amount of greenhouse gases arising from the decomposition of their organic matter in landfills.^5,6^

Despite the great research efforts that were assiduously pursued in the past few decades with the aim to produce biobased polymers, with approximately 2 million tons per year of 100% biobased plastics, which are currently produced,^3^ the same is not surely true for the unavoidable additives,^7^ which are strongly necessary to tune the properties as well as the processability of most of the biobased polymers,^8^ thus obtaining fully biobased formulations. Indeed, approximately 70% of more than 8 million metric tons of plasticizers sold worldwide yearly are still based on o-phthalic acid esters (e.g., di-2-ethylhexyl phthalate or DEHP, diisononyl phthalate or DINP, and diisodecyl phthalate or DIDP), even if they are the object of intense environmental and human health-related concerns.^9^

Levulinic acid (LA) is receiving increasing attention due to its versatility as a building block. It is considered as one of the top 12 auspicious biomass derivatives for the synthesis of high-added value materials, among which are plasticizers.^10^ Moreover, LA and volatile fatty acids (VFAs) represent feasible cosubstrates to produce biopolyesters like polyhdroxyalkanoates (PHAs),^11^ which are considered one of the most promising classes of biopolymers^12^ due to their excellent biocompatibility and biodegradability.^13^ However, these biopolyesters are generally semicrystalline (up to 80%),^14^ thus being stiff and brittle. This hinders their use in selected applications if they are not compounded with opportunely selected bioadditives.^15^ Among PHAs, poly(3-hydroxybutyrate) (PHB) and related copolymers are the most known and they have been studied for several technological applications.^16−20^ Among the phthalate alternative plasticizers for PHA, the most employed are alkyl citrates or adipates and epoxidized vegetable oils (EVOs).^21^ However, alkyl citrates and adipates cannot be compared with phthalates in terms of plasticization efficiency and versatility. Their limited plasticization effect results in the need of an increased amount of additives to obtain the desired modification with the consequent increase in the polymeric compound cost.^21^ EVOs are another well-known family of biobased plasticizers that, despite their significant plasticization performance, show a quite high leaching rate (exudation), especially under UV irradiation.^22^ Moreover, EVO production (based on the Prilezhaev process) involves harsh conditions and the use of corrosive and harmful reagents, making this process environmentally unsustainable.^23^ Since 2002, the 1,2-cyclohexane dicarboxylic acid diisononyl ester (commercially known as DINCH) has been increasingly used as a substitute for phthalates, and nowadays, it represents probably the most known commercially available alternative. Nonetheless, recent studies have shown that prolonged exposure to DINCH can cause severe health problems.^24,25^

Some of the authors optimized an approach to effectively obtain LA from steam exploded **Cynara cardunculus** L. residual biomass through an acid-catalyzed hydrolysis.^26^*C. cardunculus* L. is an infesting plant that has been recently recognized as a remarkable source of chemicals, also because of its minimal cultivation inputs, leading to high adaptability.^27^ The high purity (i.e., 93%) LA obtained after purification was then employed for the synthesis of five ketal-diester derivatives that demonstrated remarkable plasticizing effectiveness when added to both poly(vinyl chloride)^28^ and poly(3-hydroxybutyrate).^29^ In this latter case, they did not significantly affect the cytocompatibility and biodegradability typical of the PHA class of biopolymers.

However, biodegradable biobased plastics and additives are not devoid of even significant environmental impacts.^1,30^ Therefore, the risk potentially associated with the production and use of these biomaterials to simply shift the environmental impacts to a different phase of their life cycle must be avoided. For this reason, the application of LCA (Life Cycle Assessment) methodology^31^ was recognized as imperative to reach clear evidence of their higher environmental sustainability with respect to petrochemical alternatives.^32^

This work aims at quantifying the environmental burdens associated with the synthesis of the innovative bioplasticizers obtained from LA derived from residual *C. cardunculus* L. biomass through a cradle-to-gate LCA approach. Although the further phases of the bioplasticizer life cycles would be necessary to be comprised in the holistic evaluation of their environmental sustainability, the here presented LCA results have been combined with the thermal and mechanical properties induced by the additives when added to PHB. This allows us to also comprise environmental sustainability considerations when assessing different bioplasticizers.

## Experimental Section

Agronomic data on the *C. cardunculus* L. crop are detailed in Table S1. They refer to 1 ha production carried out in Porto Torres, Sardinia Region, Italy, by Novamont S.p.A.^33^

The experimental procedures employed during the following phases are reported elsewhere^26−28^ and only summarized hereafter.

### Residual *C. cardunculus* L. Biomass Steam Explosion Pretreatment

The *C. cardunculus* L. epigean residue was chipped using a stationary electric chipper and then left to dry at room temperature. The dried biomass was subjected to overnight acid impregnation by using 98% H_2_SO_4_ at 1.5 wt % with respect to the whole mass, calculated to have a solid-to-liquid ratio of 1:10. The biomass was then separated from the liquid by filtration and immediately inserted into a CRB/CIRIAF steam explosion reactor. The latter is composed of a vapor generator, a charging section for the raw biomass, expansion valves, a high-pressure reactor, a post-explosion tank and a recovery section for exploded liquid.^34^ A dry epigean residue ( 447.5 g) was treated at 165 °C and 200 bar for 10 min, employing a severity factor log R0 = 2.91.^26^

At the end of the treatment, the biomass was left to decant, and then the solid part was collected and pressed to remove the liquid still present in the fibers. The solid part was then washed by immersion in 50 °C hot water (solid-to-liquid volume ratio of 1:10) for 30 min and then pressed again and stocked.

### Levulinic Acid Obtainment through Acid-catalyzed Hydrolysis of Exploded *C. cardunculus* L. Biomass

LA was obtained from the exploded biomass by a HCl-catalyzed hydrolysis as detailed elsewhere.^26^ Exploded and crushed biomass (4.98 g) were inserted in a 35 mL Pyrex vial and added with deionized water and concentrated HCl to reach biomass and catalyst loadings of 20 and 1.5 wt %, respectively, and a substrate-to-catalyst ratio of 2.0 mol/mol. The mixture was inserted in a single-mode microwave applicator (CEM Discover S-class) and treated at 190 °C for 40 min. The hydrolyzed products were separated from solid residues by vacuum filtration through a PTFE (poly(tetrafluoroethylene)) filter (0.2 μm).

Approximately 20 mL of the crude hydrolyzed products was extracted by liquid–liquid extraction with 60 mL of 2-methyltetrahydrofuran (2-methyl THF) for 4 h. The organic fraction was then separated and subsequently subjected to fractional distillation. A first distillation step under atmospheric pressure allowed removal of the solvent. The oil bath temperature was progressively increased up to 195 °C, and the pressure was progressively decreased up to 5 mbar to separate first any lights, and then LA, which was finally dried. It was characterized by a purity of 93%, as ascertained by high-performance liquid chromatography (HPLC) and gas chromatography (GC).^26,35^ The yield of the isolated product resulted in 20.3 wt % with respect to the starting biomass.^26^

### Three-Step Synthesis of Ketal-Diester Derivatives of Levulinic Acid

**Scheme 1:**
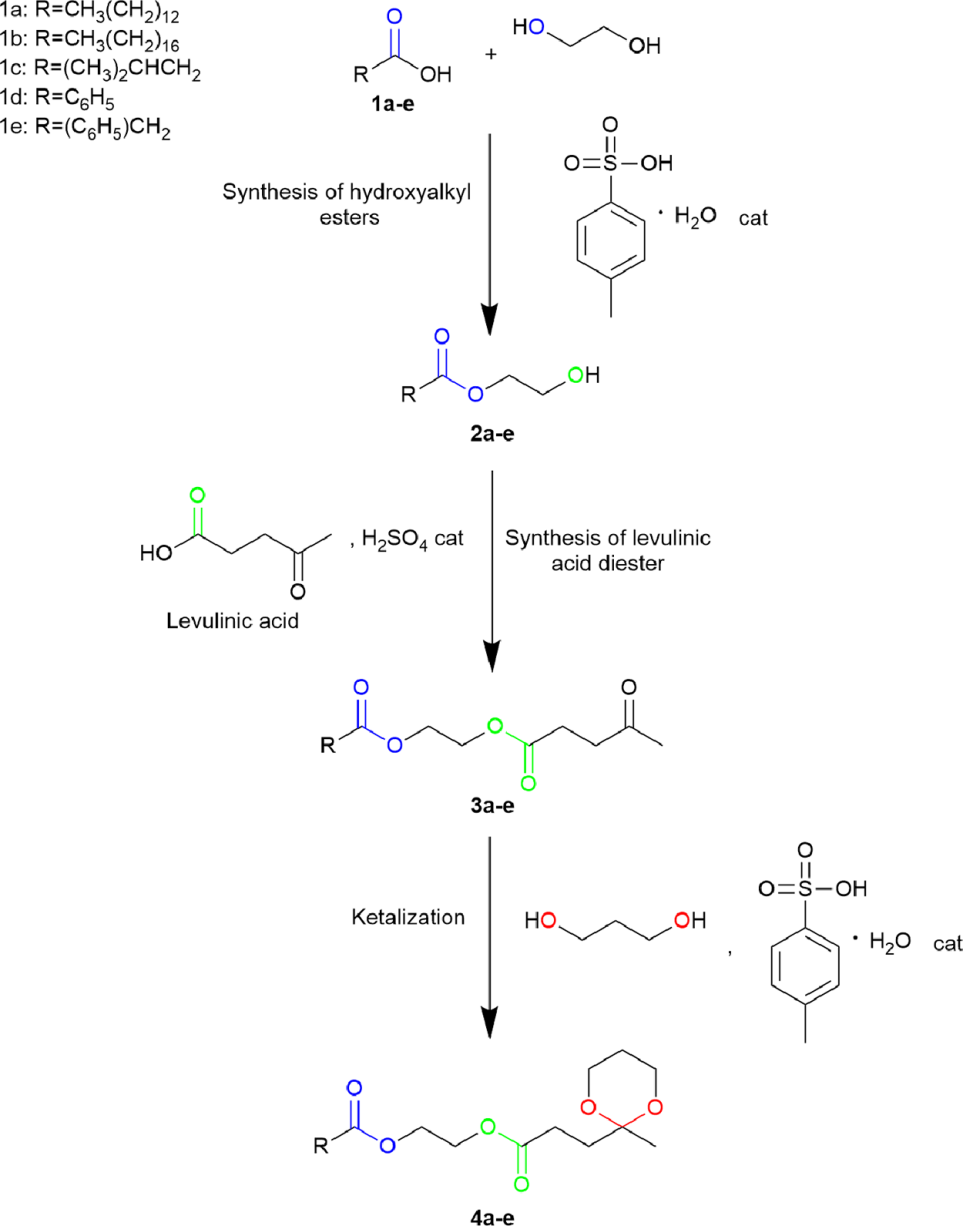
Reaction Scheme of the Three-Step Synthesis of Ketal-Diester Derivatives from Levulinic Acid. Adapted from Sinisi et al.^28^

Five different ketal-diester derivatives of LA were synthesized according to the procedure by Sinisi et al.,^28^ which is summarized in Scheme 1.

During the first step, 5 mmol of the carboxylic acid **1a**–**e** (Scheme 1, i.e., myristic, stearic, isovaleric, benzoic, and phenylacetic acids) and 0.25 mmol of *p*-toluensulfonic acid monohydrate were added to 100 mmol of ethylene glycol in a round-bottomed flask and magnetically stirred at 75 °C for 18 h. The mixture was then cooled and quenched with a 10 wt % aqueous Na_2_CO_3_ solution. The aqueous phase was then extracted twice by employing diethyl ether, which was then collected and washed with water and brine, dried with anhydrous Na_2_SO_4_, and finally evaporated under reduced pressure.

The as-obtained 2-hydroxyethyl ester **2a**–**e** (3 mmol) (Scheme 1) was added to the biomass-derived LA (9 mmol) and dissolved in toluene (0.15 M with respect to the limiting reagent) in the second step of the reaction. Then, a drop of 96% H_2_SO_4_ was added as a catalyst. The reaction mixture was maintained at 140 °C for 7 h, after which the same workup procedure followed for **2a**–**e** was applied.

In the last step, 1 mmol of the as-synthesized diester **3a**–**e** (Scheme 1) and 3 mmol of 1,3-propanediol were dissolved in toluene under stirring. *p*-Toluenesulfonic acid monohydrate (1 mmol) was added as a catalyst. The reaction was prolonged at 140 °C for 7 h, after which the same workup procedures described for **2a**–**e** were performed followed by the purification of the extracted residue by column chromatography (*n*-hexane/ethyl acetate 5:1) giving the ketal diesters in yields of 55% (**4a**), 60% (**4b**), and 50% (**4c**–**e**).^28^

## Life Cycle Assessment (LCA)

LCA was applied according to the ISO 14040-14044,^36,37^ as detailed hereafter.

### Goal and Scope Definition

#### Goal Definition

The goal of this study was to quantify “from cradle to gate” the environmental impacts associated with five different ketal-diester derivatives of LA (the latter obtained from the *C. cardunculus* L. epigean residue) and to compare them to consider also their environmental performances along with the mechanical and thermal ones when employed to increase the processability of biobased polymers.

#### System, Functional Unit, and Function of the System

The system object of this study is the preparation of five different ketal-diester derivatives of levulinic acid to be used as bioplasticizers for biobased polymers. The functional unit selected for each process was the amount of product experimentally obtained during its production as described in the previous experimental section. 1 g of each bioplasticizer was selected as the functional unit for their environmental impact comparison.

However, since the function of the studied system is to enhance the mechanical and thermal properties of biobased polymers, such as polyhydroxyalkanoates, the comparison among products **4a**–**e** was also performed by considering the amounts effectively employed to prepare poly(3-hydroxybutyrate) (PHB) compounded films.^29^ Particularly, **4a**–**e** were added at 10 and 20 per hundred of resin (phr) to 250 mg of PHB solubilized in CHCl_3_ (12.5 mg mL^–1^) and subsequently cast in a Petri dish to obtain compounded films of approximately 70 μm thickness.^29^

**Figure 1:**
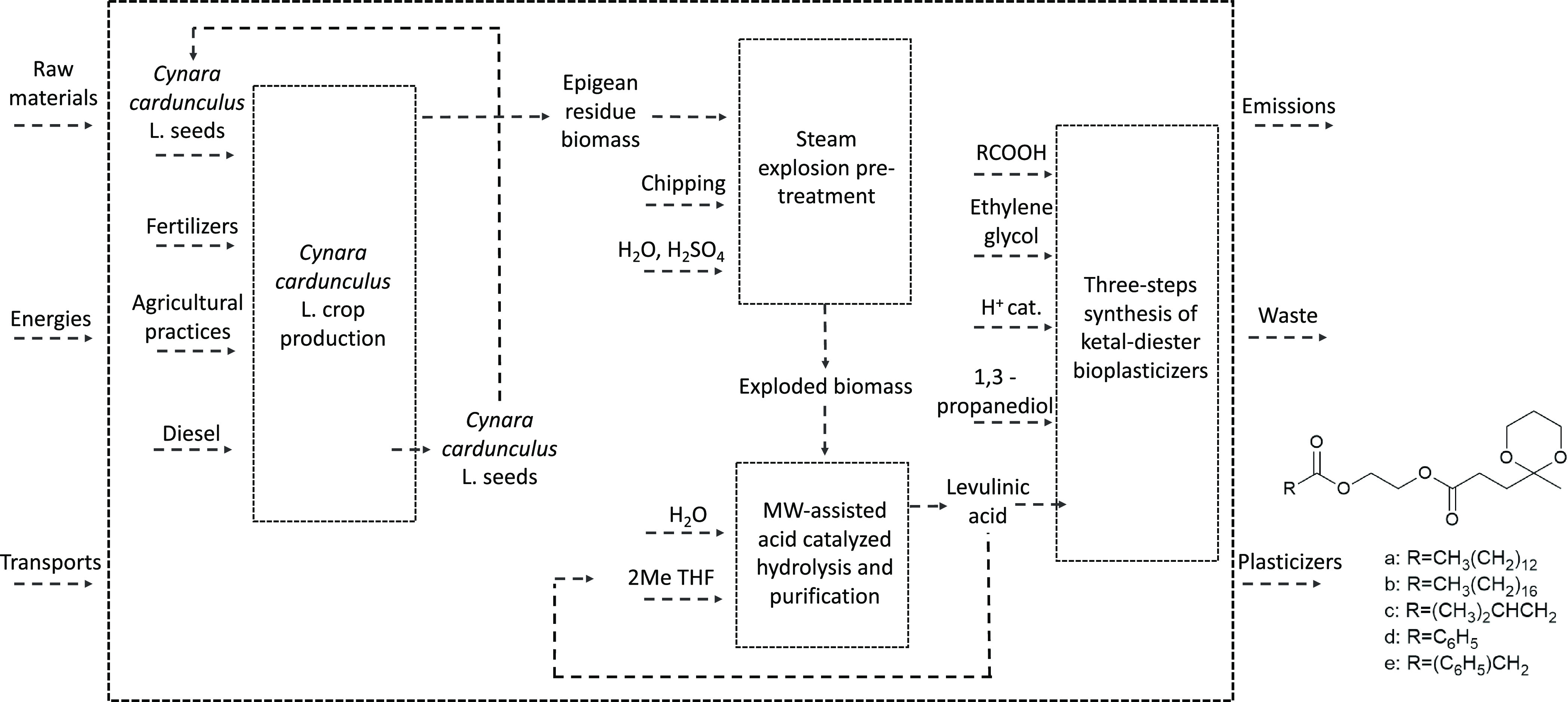
Flowchart summarizing the overall system boundaries considered in the LCA study for the synthesis of five innovative ketal-diester bioplasticizers obtained from residual *C. cardunculus* L. biomass-derived levulic acid.

The system boundaries (summarized in Figure 1 and detailed for each modeled phase in Figures S1–S4) range from crop production to biomass pretreatment and to the obtainment of LA through microwave-assisted acid-catalyzed hydrolysis and subsequent purification, up to its final use for the synthesis of the five bioplasticizers.

### Life Cycle Inventory (LCI) and Life Cycle Impact Assessment (LCIA)

The data for the LCI were mostly primary and thus collected during the experimental activities.

The modeling of the processes was done by employing data sets of the Ecoinvent database (EID, version 3.8),^38,39^ and particularly, an attributional approach was followed.^38^

A distance of 100 km was considered for transport contributions. Particularly, the road freight transport by diesel EURO 6 lorries was assumed with two different lorry capacities of 3.5–7.5 and 16–32 t.^40,41^

The potential emissions to the atmosphere were also considered in the modeled chemical processes. Advanced process calculations were performed rather than the basic process ones.^42^ Particularly, the working losses, *L*_w_,^43^ were considered and calculated, as detailed in eq 1, where *V* is the volume of the chemical (l), *V*_m_ is the molar volume (l) of ideal gas at 0 °C and 1 atm, *T* is the average temperature (*K*), *P*_*i*_^sat^ is the vapor pressure of liquid (mmHg), MW is the molecular weight of the chemical (g·mol^–1^), *K*_N_ is the turnover factor (dimensionless and equal to 1 in the present study), and *K*_P_ is the working loss product factor (dimensionless), equal to 1 for organic liquids.
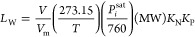
1

It was considered that 99% of each emitted substance was retained by an aspiration system endowed with an activated carbon filter.^40^

The complete inventories of *C. cardunculus* L. crop production are detailed in Tables S2 and S3. Due to its multioutput character, an economic allocation was applied between the main product (i.e., the seeds) and the coproduct (i.e., the epigean residue), as detailed in Table S3. The biomass was considered to capture carbon dioxide from air (indicated as input from nature in Table S3) and to partially rerelease it into the atmosphere (indicated as emission of CO_2_, biogenic in Table S3), similarly to what is typically considered for the agricultural production systems by the Ecoinvent database.^44^

The inventories of biomass pretreatment are detailed in Tables S4–S10. For the steam explosion process (inventoried in Table S10), the exploded biomass was considered as the only valuable output, with the rest of the outputs accounted as emissions and wastes (except for the recovered fractions of sulfuric acid and water, which instead were considered as avoided products).

The inventories related to the obtainment of purified LA are reported in Tables S11–S20. The purified LA was considered as the only valuable output of this process (Table S20). The further outputs were represented as emissions and waste treatments, in accordance with what was experimentally performed in the laboratory. Similarly, energy contributions were modeled as furnished by electricity as effectively performed during experiments.

Finally, the inventories related to the three-step synthesis of bioplasticizers **4a**–**e** (Scheme 1) are detailed in Tables S21–S40. In these inventories, bioplasticizers **4a**–**e** (Tables S36–S40) and their precursor compounds **3a**–**e** (Tables S31–S35) and **2a**–**e** (Tables S26–S30) represent the sole valuable outputs of the systems. The remaining outputs are emissions and waste treatment processes. Again, this choice reflects what was experimentally conducted during the synthetic procedures. The recovered fractions of the organic solvents used during each of the three synthetic steps were considered as avoided products. Again, in these lab-scale procedures, electric energy contributions were employed and therefore modeled.

All the inventories were modeled in SimaPro 9.3.0.2.^45^ The life cycle impact assessment (LCIA) phase was conducted by employing the global scale-oriented method ReCiPe2016 both at midpoint and endpoint levels, with a hierarchist (H) perspective and average weighting set (A).^46^ This method is one of the most widely accepted and applied global methods,^47−49^ comprising a high number of impact categories.^50^

## Results and Discussion

### Environmental Impacts of Ketal-Diester Derivatives of Levulinic Acid **4a**–**e**

The potential environmental impacts associated with 1 g of each of the five different bioplasticizers (**4a**–**e** in Scheme 1) are detailed at a midpoint level in Table 1. Their relative percentage values are summarized for each impact category in Figure S5.

**Table 1:** Midpoint Environmental Impacts (ReCiPe 2016, H) Associated with the Obtainment of 1 g of the Five Different Bioplasticizers **4a**–**e** (Scheme 1)

impact category	unit	plasticizer **4a**	plasticizer **4b**	plasticizer **4c**	plasticizer **4d**	plasticizer **4e**
global warming	kg CO_2_ equiv	30.1	20.4	42.7	39.1	38.1
stratospheric ozone depletion	kg CFC11 equiv	1.81 × 10^–05^	1.22 × 10^–05^	2.56 × 10^–05^	2.34 × 10^–05^	2.28 × 10^–05^
ionizing radiation	kBq Co-60 equiv	2.59	1.73	3.65	3.33	3.25
ozone formation, human health	kg NOx equiv	0.0481	0.0324	0.0681	0.0622	0.0607
fine particulate matter formation	kg PM2.5 equiv	0.0330	0.0222	0.0467	0.0427	0.0417
ozone formation, terrestrial ecosystems	kg NOx equiv	0.0492	0.0332	0.0697	0.0637	0.0622
terrestrial acidification	kg SO_2_ equiv	0.0914	0.0616	0.129	0.118	0.115
freshwater eutrophication	kg P equiv	0.0107	7.31 × 10^–03^	0.0153	0.0140	0.0136
marine eutrophication	kg N equiv	8.20 × 10^–04^	5.47 × 10^–04^	1.15 × 10^–03^	1.05 × 10^–03^	1.04 × 10^–03^
terrestrial ecotoxicity	kg 1,4-DCB	107	72.4	152	139	135
freshwater ecotoxicity	kg 1,4-DCB	1.14	0.773	1.62	1.48	1.44
marine ecotoxicity	kg 1,4-DCB	1.51	1.02	2.14	1.96	1.91
human carcinogenic toxicity	kg 1,4-DCB	2.10	1.41	2.96	2.71	2.64
human noncarcinogenic toxicity	kg 1,4-DCB	23.4	15.9	33.3	30.5	29.7
land use	m^2^a crop equiv	0.896	0.598	1.26	1.15	1.12
mineral resource scarcity	kg Cu equiv	0.137	0.0859	0.187	0.170	0.166
fossil resource scarcity	kg oil equiv	7.59	5.11	10.7	9.82	9.58
water consumption	m3	0.494	0.329	0.695	0.634	0.620

The environmental impacts obtained and reported in Table 1 (e.g., global warming potential values ranging from *ca*. 20 to 43 kgCO_2_ equiv/g bioplasticizer) are significantly higher with respect to those reported for both conventional phthalates (i.e., DEHP^51,52^ and DINP^53^) and commercially available bioplasticizers.^54^

Despite the differences in the impact assessment method employed and the modeling choices performed, this is necessarily to be ascribed to the laboratory-scale extent of the here modeled processes. Indeed, when moving from a laboratory scale to a pilot scale and to an industrial one, the environmental impacts can be significantly reduced,^55^ mainly because of the lower direct inputs of materials and direct energy consumptions (the latter also derived by sources different from electrical devices as in the present study).^56^ Therefore, the values reported in Table 1 are expected to be reduced to a great extent when the synthesis of bioplasticizers **4a**–**e** is upscaled, thus reaching a level of commercial readiness similar to those of conventional plasticizers and already commercially available bioplasticizers.

However, it needs to be reminded that the main goal of LCA studies performed at a lab scale is to identify the dominating hotspots for the development of new processes/products.^57^ Furthermore, the relative impact results of the comparative study performed should not be revolutionized when moving to a larger scale, since the identical experimental procedures characterizing the three-step syntheses lead to bioplasticizers **4a**–**e**.

The obtained environmental impacts follow the general trend **4b** < **4a** < **4e** < **4d** < **4c**, independently by the impact category considered.

Due to the similar experimental procedures leading to **4a**–**e**, only the **4b** derivative, i.e., the one characterized by the lowest midpoint environmental impacts, was investigated in full detail to identify the substances and the processes that mostly contribute to each impact category.

Particularly, in the global warming impact category, the environmental impact is 20.4 kg of CO_2_ equiv, and it is mainly (for 90.6%) due to the release in air of 18.5 kg of carbon dioxide, a fossil substance. This emission is mostly (for 18.6%) associated with the EID process “Spent solvent mixture {Europe without Switzerland}| treatment of spent solvent mixture, hazardous waste incineration, with energy recovery | APOS, U”. This is mainly associated with the treatment of the reaction wastes generated in the third synthetic step (for 74.3%) and with the obtainment of the diester precursor **3b**.

The impact in the “stratospheric ozone depletion” category results equals to 1.22 × 10^–5^ kg CFC11 equiv. This impact is mainly (for 76.4%) due to the emission in air of 845.5 mg of dinitrogen monoxide related for 18.2% to the process “Electricity, high voltage {IT}| market for | APOS, U”, associated with the Italian energy mix consumed during the preparation of precursor **3b** (for 50.4%) and the last ketalization step performed for 7 h at 140 °C (for 41.2%).

The synthesis of 1 g of **4b** leads to an impact of 1.73 kBq Co-60 equiv in the category “ionizing radiation”. This is due to 95% of the emission into air of an amount of Radon-222 corresponding to 1130.7 kBq. The process responsible for most (97.2%) of this emission is “Tailing, from uranium milling {GLO}| treatment of | APOS, U”, representing the treatment of tailings deriving from milling operations performed on the extracted uranium ore. This process is related to the Italian mix of electric energy necessary in the first two synthetic steps (for 49.0%) and during the operation of the heating plate in the last step of reaction (for 37.8%).

The value of the “ozone formation, human health” category is 3.24 × 10^–2^ kg NO_*x*_ equiv, and it is essentially (for 96%) due to the emission of 31.1 g of nitrogen oxides associated for 18.3% to the EID process “Electricity, high voltage {IT}| electricity production, hard coal | APOS, U”, comprised in the energy mix considered and necessary (for 50.4%) to synthesize precursor **3b** and (for 41.2%) to its ketalization.

The same emission is also responsible for 93.8% of the impact category “ozone formation, terrestrial ecosystems”.

The environmental load referring to the category “fine particulate matter formation” corresponds to 2.22 × 10^–2^ kg PM2.5 equiv. The main culprit (63%) of this environmental issue is the emission of 48.3 g of sulfur dioxide in air. Also, in this latter case, the process “Electricity, high voltage {IT}| electricity production, hard coal | APOS, U” contributes the most (for 23.5%).

The above-mentioned amount of SO_2_ emitted into air also represents the main contribution (78.4%) to the category “terrestrial acidification”.

The impact in the category “freshwater eutrophication” is 7.31 × 10^–3^ kg P equiv. It is essentially (for 77.8%) due to the emission of 17.2 g of phosphate into water. This emission is associated for 45.2% to the process “Spoil from hard coal mining {GLO}| treatment of, in surface landfill | APOS, U”, the latter being mainly comprised in the processes leading to precursor **3b** (for 48.1%) and in the electric energy needed for the operation of the hot plate in the last step of synthesis (for 27.6%). The same EID process is also responsible for the emission of 7.7 g of nitrate in water, corresponding to 95% of the environmental impact in the category “marine eutrophication”.

For the three impact categories related to ecosystem toxicity issues, the substance mainly responsible is copper, emitted in air (contributing for 71.7% to “terrestrial ecotoxicity”) and in water (contributing for 59.8 and 53.9% to “freshwater ecotoxicity” and “marine ecotoxicity”, respectively). In the first case, the process “Copper, anode {RoW}| smelting of copper concentrate, sulfide ore | APOS, U” represents the main contribution (i.e., 62.4%), and it is related to the copper used to model the aspiration system (for 42%) used in the third step of synthesis and in the previous ones leading to precursor **3b** (overall for 50.4%).

The emission of copper in water is instead associated with the process “Scrap copper {Europe without Switzerland}| treatment of scrap copper, municipal incineration | APOS, U”, mainly related to the end-of-life treatment of the electricity transmission network, comprised in the electricity mix used to obtain precursor **3b** and the final bioplasticizer **4b**.

The impact in the category “human carcinogenic toxicity” is 1.41 kg 1,4-DCB equiv. For 94.6%, it is due to the emission of 178.9 mg of chromium VI in water. This is related for 53% to the process “Electric arc furnace slag {RoW}| treatment of electric arc furnace slag, residual material landfill | APOS, U”. The latter describes the end-of-life treatment of wastes deriving from the production of the steel necessary for the aspiration system, used in the last synthetic step (for 25.9%) and in the previous ones leading to **3b** (for 49%).

The noncarcinogenic toxicity in humans is 15.9 kg 1,4-DCB equiv. This is due to 53.2% to 1.04 g of zinc emitted in water. The process mainly responsible (for 22.9%) for this emission is “Spoil from hard coal mining {GLO}| treatment of, in surface landfill | APOS, U”, which is related to the same subprocesses and in the same percentages as already discussed for the “freshwater eutrophication” impact category.

The impact in the category “land use” is 0.6 m2a crop equiv. It is due for 60.6% to land transformation (from forest, intensive). The process mainly responsible (i.e., for 31%) is “Wood chips, wet, measured as dry mass {SE}| hardwood forestry, birch, sustainable forest management | APOS, U”. It is comprised in the production of the electric energy used to synthesize compound **3b** (for 49.2) and to heat this latter at 140 °C for 7 h during its ketalization.

The category “mineral resource scarcity” has an impact of 8.59 × 10^–2^ kg Cu equiv. This contribution is due for 27.9% to the extraction of 387.1 g of iron, on its turn associated for 82.3% to the EID process “Iron ore, crude ore, 46% Fe {GLO}| iron ore mine operation, 46% Fe | APOS, U”. This process is comprised in the production of the steel necessary for the aspiration system used in the last reaction step (for 33.6%) and for the obtainment of precursor **3b** (for 49.1%).

Extraction of 3.3 m^3^ of natural gas is the main contribution (54.8%) to the impact category “fossil resource scarcity”. It is associated for 48.6% to the process “Natural gas, high-pressure {RU}| natural gas production | APOS, U”, that is comprised in the electricity mix used to obtain precursor **3b** (for 49.6%) and the final bioplasticizer **4b** through the last ketalization step performed by heating the reaction mixture at 140 °C for 7h.

The impact in the “water consumption” category is 0.33 m^3^, as a consequence of the consumption of “water, turbine use, unspecified natural origin, IT” that is mainly (for 96.7%) comprised in the process “Electricity, high voltage {IT}| electricity production, hydro, run-of-river | APOS, U”. The latter process is related to the operation of the heating plate in the last reaction step (for 41.2%) and to the obtainment of diester **3b** (for 50.4%).

**Figure 2:**
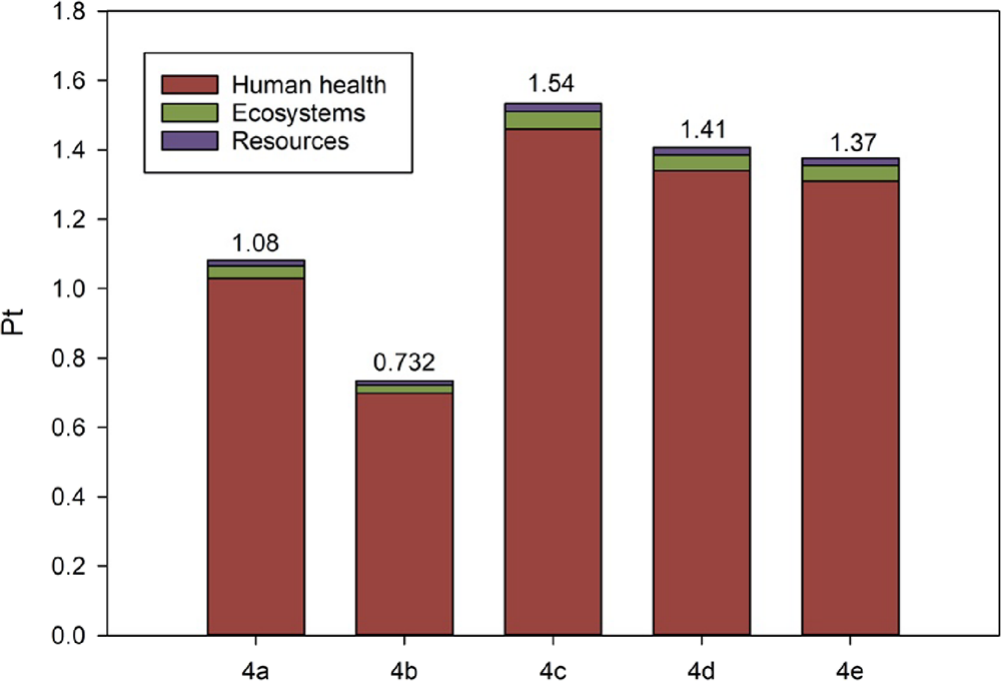
Single score results for the synthesis of 1 g of each of the five bioplasticizers **4a**–**e**.

The endpoint results can be obtained by grouping the results of the 18 impact categories into the opportune damage categories (i.e., human health, ecosystems, and resources) and referring them at the point at which the environmental effects potentially occur. They are reported in Table S41 and Figure S6 for all five bioplasticizers. The environmental loads associated with the ketal-diester derivatives **4a**–**e** can be better compared if expressed as a single score (i.e., in terms of the ecoindicator point, Pt: the smaller the value, the lower the potential environmental impact of that particular product or process results) as calculated after normalization and weighting operations. Particularly, global normalization factors for reference year 2010, included in the ReCiPe 2016 endpoint method, were applied. The “A” (i.e., average) weighting set was selected: this latter considers the weighting factors of Eco-indicator 99 (i.e., 400 for human health, 400 for ecosystems, and 200 for resources).^58^ The single score results are detailed in Table S42 and depicted in Figure 2. From Figure 2, it is immediately visible that the most affected damage category is human health, independently by the bioplasticizer considered, followed by the ecosystems and resources ones. The most responsible impact category is global warming, human health in all five different cases. Particularly its contribution to the damage category, human health is approximately 45% for all the bioplasticizers, with the substance contributing most (i.e., for *ca.* 91% independently of additives **4a**–**e**) to that impact category being carbon dioxide, fossil.

The ketal-diester derivative of levulinic acid obtained by stearic acid precursor (**4b**) is characterized by the lowest single score, i.e., 0.732 Pt, while on the opposite hand, the bioplasticizer **4c** obtained by employing isovaleric acid is the one possessing the highest impact, i.e., 1.54 Pt.

Again, 1 g of bioplasticizer **4b** was selected as the representative example among the five different ketal diesters to determine and quantify the role of the different subprocesses into the environmental impacts associated with its life cycle phases. Particularly, they are detailed and depicted, in terms of single score, in Table S43 and Figure S7, respectively.

The major contributions to the environmental impact of 1 g of bioplasticizer **4b** are due to the diester derivative **3b** (for 44.2%), to the electric energy necessary for the functioning of the heating plate for 7 h (for 23.0%), and to the waste treatment considered for the reaction and workup procedure waste (for 6.7%).

**Figure 3:**
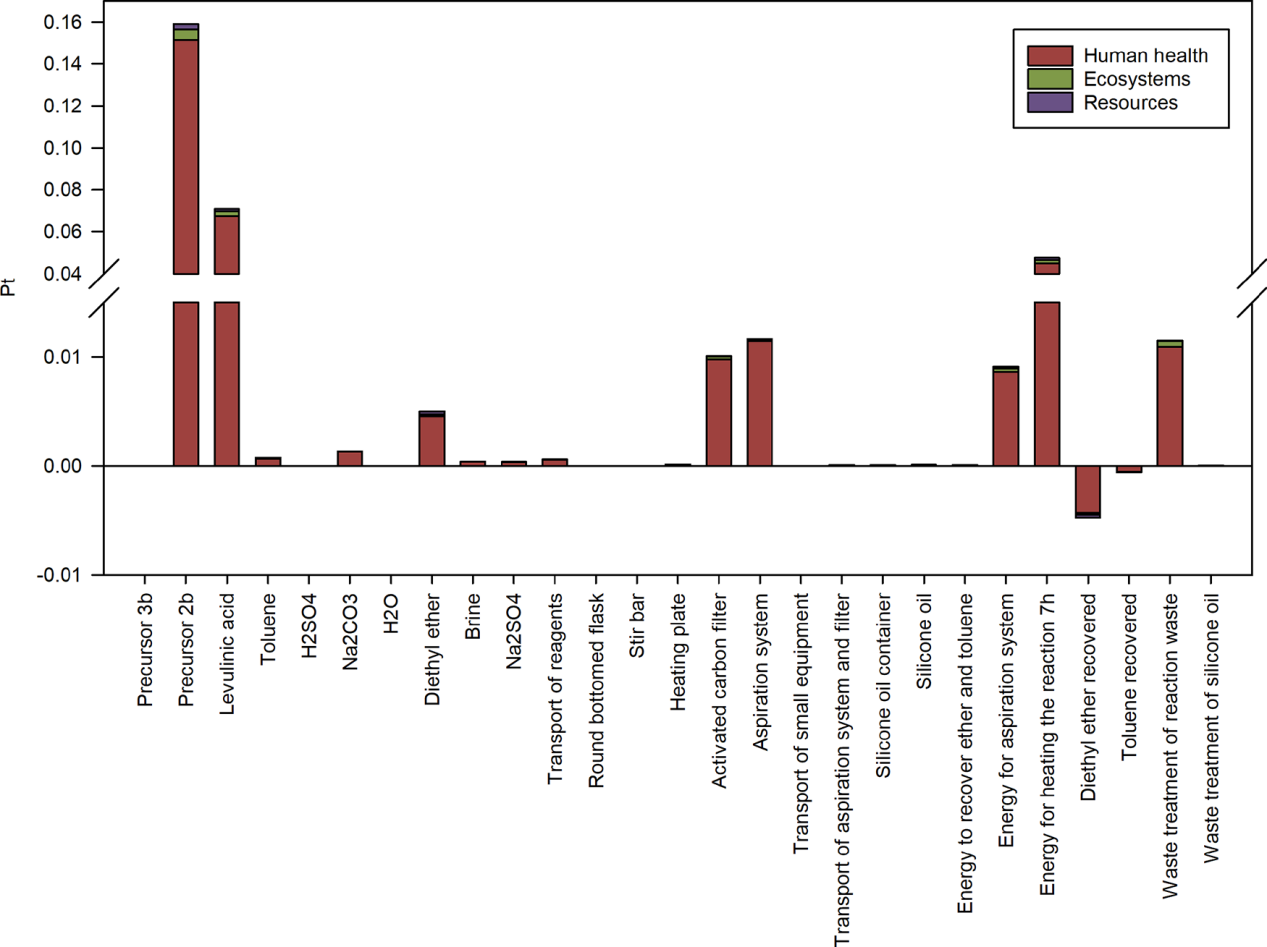
Single score results (ReCiPe 2016 H/A) associated with the production of 1.1314 g of the diester precursor **3b**.

By analyzing in more detail the environmental impacts associated with the synthesis of precursor **3b**, the single score results detailed in Table S44 are obtained, which are instead summarized in Figure 3 (referring to 1.1314 g, which corresponds to the amount of **3b** experimentally needed to obtain 1 g of plasticizer **4b**), from which the contribution of biomass-derived LA is visible, representing the second for importance contribution to the whole impact (i.e., 21.9%), preceded by precursor **2b** (for 49.1%), and followed by electric energy needed to conduct the reaction at 140 °C for 7 h (for 14.7%).

**Figure 4:**
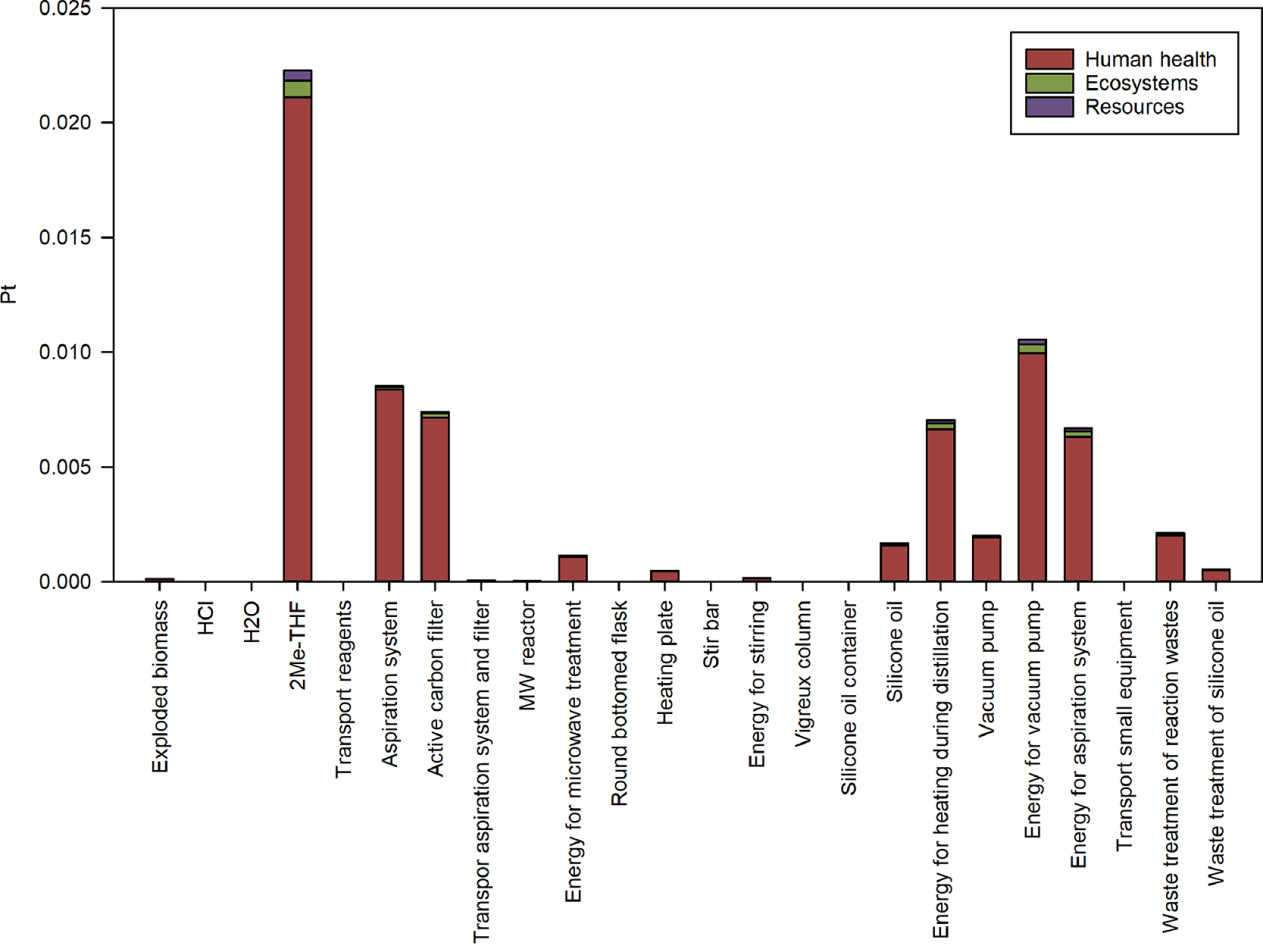
Single score results (ReCiPe 2016 H/A) associated with the obtainment of 1.0166 g of levulinic acid from exploded *C. cardunculus* L. biomass through a microwave-assisted acid-catalyzed hydrolysis followed by purification.

More interestingly, the environmental impacts of LA derived from the residual biomass can be better investigated by calculating its single score results. The calculation was performed for 1.0166 g of LA, i.e., the amount necessary to obtain 1.1314 g of **3b**. The results are listed in Table S45 and summarized in Figure 4. The higher contribution is 2-methyl THF used as the solvent for liquid–liquid extraction from the crude hydrolyzed product. However, it is worthy to be mentioned that for the modeling of this chemical substance, it has been considered its synthesis from the same levulinic acid for the purification of which it is used, according to the hydrogenation procedure described elsewhere.^59,60^

Although the contribution of exploded biomass is extremely low, accounting only for 0.2% (Figure 4), the contributions to its impact and those related to the residual biomass as harvested are detailed in Tables S46 and S47, respectively, and summarized in Figures S8 and S9, respectively.

The environmental impact associated with 5.008 g of exploded biomass (i.e., the amount necessary to obtain the above considered 1.066 g of LA) is 1.44 × 10^–4^Pt (Table S46 and Figure S8). Most (50.57%) of this impact is due to the end-of-life treatment considered for wastes containing H_2_SO_4_ used for the impregnation of the biomass, even if 70% of the impregnation solution was recovered. The epigean residue accounts only for 5.67% of the whole impact of the exploded biomass. This is also a consequence of the allocation criteria considered (i.e., economic one) in the modeling of the multioutput agricultural process leading to both *C. cardunculus* L. seeds (1.5 t/ha) and epigean residue (15 ton/ha). According to this allocation, only 7.13% of the entire damage is to be attributed to the epigean residue (see Table S3 for details).

By analyzing the environmental impact of the epigean residue as harvested (considering an amount of relevance, e.g., 1 t), the total impact expressed as a single score is 4.076 Pt (Table S47 and Figure S9). This is mainly due (for 53.86%) to the direct emissions into air, water, and soil characterizing the agricultural process itself, which were calculated according to the Ecoinvent report for the inventories of agricultural production systems^44^ (see Table S3 for details). These direct emissions prevalently (for 91.69%) affect the damage category ecosystems.

### Sensitivity Analysis

The results demonstrated the great contributions of electric energy consumptions to the environmental impacts associated with the synthesis of the innovative bioplasticizers at the lab scale. Therefore, a sensitivity analysis was performed to pinpoint opportunities for improvement. This sensitivity analysis was conducted on 1 g of bioplasticizer **4b**, which was selected again as the representative example of the ketal diesters synthesized.

First, the influence of a different electric energy mix on the obtained results was investigated. Particularly, the Italian energy mix was replaced by the Swedish one since Sweden is known as a global leader in the utilization of renewable energy^61^ (the EID data set used in the first alternative scenario was Electricity, low voltage {SE}| electricity voltage transformation from medium to low voltage | APOS, U). This replacement was considered limited to the acid-catalyzed hydrolysis of exploded biomass for the obtainment of purified LA and to the three steps of synthesis leading to bioplasticizer **4b**.

Moreover, in the second alternative scenario, an increase of 30% in the reaction yields was also studied in the obtainment of purified LA and in the last step of the reaction leading from precursor **3b** to bioplasticizer **4b**. Indeed, the first two steps of reaction are already characterized by high reaction yields (80 and 91%). In this way, this second alternative scenario distinguishes itself for the 50.3% yield for the obtainment of purified and isolated LA and for the 90% yield for the ketalization of **3b** in **4b**.

Last, in the third scenario of the sensitivity analysis, the solvent recycling percentages of all the employed solvents during the three-step synthesis of **4b** were increased up to 95%, the latter already corresponding to the considered recycling percentage of the sole diethyl ether.

**Figure 5:**
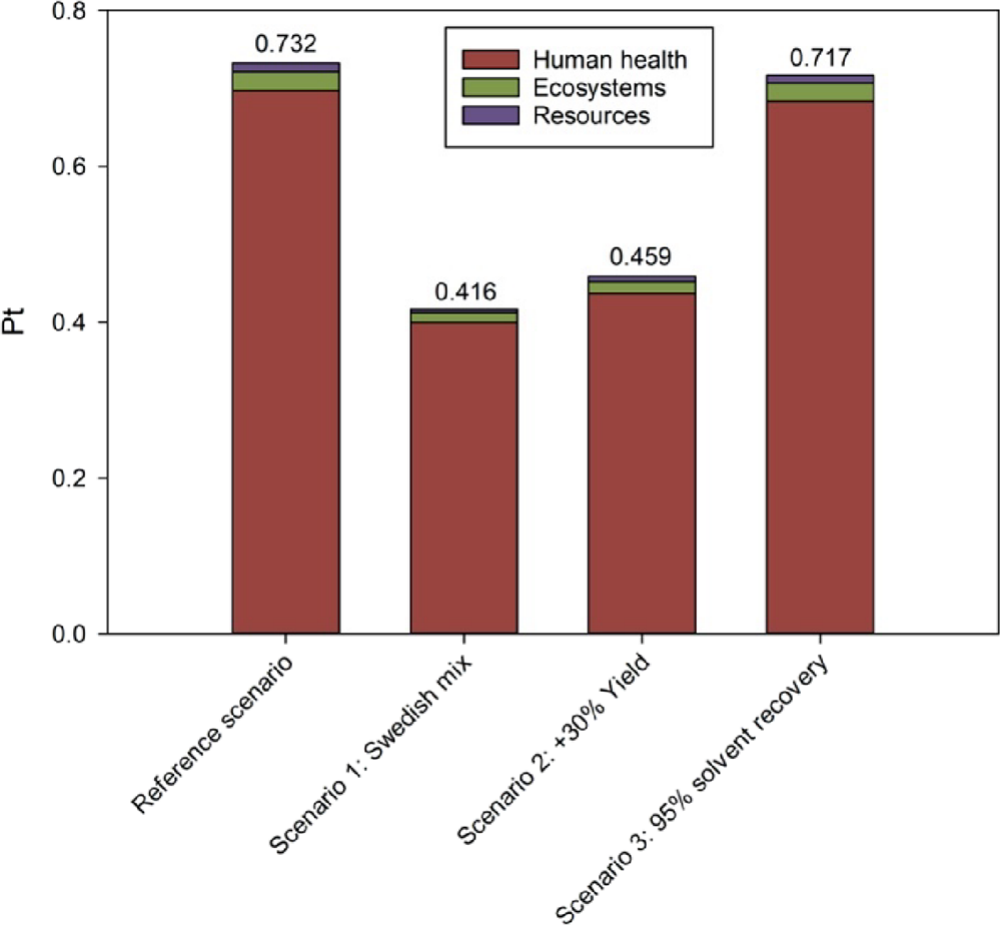
Results of the sensitivity analysis expressed as a single score (ReCiPe 2016 endpoint, H/A) and referred to the synthesis of 1 g of bioplasticizer **4b**, in accordance with the three scenarios considered alternatively to the reference one.

The results of the sensitivity analysis at a midpoint level are detailed in Table S48 and depicted in terms of relative impact percentages for each impact category in Figure S10. More immediately, the single score results depicted in Figure 5 show that the alternative scenario 1 (i.e., the one considering the Swedish electric energy mix) is the one potentially leading to the higher reduction of the overall environmental impact (−43.2%). Therefore, the possibility to significantly reduce the environmental impacts associated with the synthesis bioplasticizers **4a**–**e** when moving to larger-scale syntheses can arise from the use of more renewable energy sources, even to the detriment of some midpoint impact categories. Indeed, the scenario considering the Swedish electricity mix has the highest values in the impact categories ionizing radiation (IR, kBq Co-60 equiv), land use (LU, m2a crop equiv), and mineral resource scarcity (MRS, kg Cu equiv), as reported in Table S48 and Figure S10.

On the opposite, scenario 3, considering 95% as the recovery percentage for all solvents used in the three-step synthesis of **4b**, is characterized by the lowest reduction of the environmental impact (*ca*. 2.05%). Indeed, this latter finding is to be related to the high solvent recovery percentages (80–95%) already characterizing the reference scenario.

### Trade-Off Evaluation between Environmental and Plasticizing Performances

Despite the environmental performances associated with bioplasticizers **4a**–**e**, they necessarily need to be also considered for the function for which they were prepared, i.e., enhancing the limiting mechanical and thermal properties of polyhydroxyalkanoates.

The plasticizing performances of **4a**–**e** were previously tested^29^ in terms of reductions of glass transition temperature (*T*_g_), melting temperature (*T*_m_), and storage modulus (*E*′) induced in poly(3-hydroxybutyrate) (PHB) compounded films when added at 10 and 20 per hundred of resin (phr), leading to the results summarized in Table S49.

Overall, the plasticization performances were comparable with those of some commercially available green plasticizers. As an example, bioplasticizers **4a**, **4d**, and **4e** induced a reduction in *T*_g_ of *ca*. 16 °C when added at 20 phr to PHB, a value very close to the reduction induced by tributyl citrate when added in similar concentrations.^62^ Moreover, independently of the employed bioplasticizers **4a**–**e**, the *E*′ values obtained when added at 20 phr (<2000 MPa) are lower than the values reported for epoxidized vegetable oil plasticizers added in a similar content.^63^

For each entry of Table S49, it has been also reported the environmental impact expressed as single score results (i.e., Pt) and recalculated (ReCiPe 2016 H/A) for the plasticizer content added that leads to that modification of each studied property (i.e., 25 mg for entries labeled as **-10** and 50 mg for entries labeled as **-20**).

**Figure 6:**
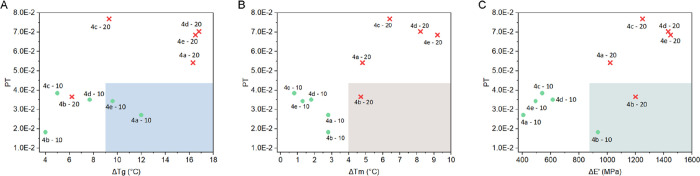
Reductions in (A) glass transition temperature (*T*_g_), (B) melting temperature (*T*_m_), and (C) storage modulus (*E*′) as a function of the single score environmental impacts of the bioplasticizers **4a**–**e** added at 10 (green dots) and 20 (red crosses) phr to 250 mg of PHB. The colored bottom right quarters represent the regions characterized by simultaneous low environmental impacts and high plasticization effect, evaluated for a specific parameter.

The plasticizing effect of each additive, with respect to neat PHB, increases when the plasticizer content is increased from 10 to 20 phr. The same is true for the environmental impact that obviously doubles when doubling the additive content. Therefore, by plotting the data of Table S49, as Pt as a function of Δ*T*_g_(°C), Δ*T*_m_(°C), and Δ*E*′ (MPa) (depicted in Figure 6 A–C, respectively), the entries characterized by 10 phr (green dots) of the plasticizer should generally lie on the bottom left quarters, while those entries characterized by 20 phr (red crosses) of the plasticizer lie on the top right ones.

However, the different ketal diesters’ side chains (aliphatic for **4a**–**c** and aromatic for **4d**–**e**) lead not only to different plasticizing effects but also to significant differences of the environmental impacts (as shown in Figure 2). These differences result in a change of the expected positions for some entries in Figure 6A–C, which were found on the bottom right quarters of each plot. This is clear for the ketal-diester plasticizer **4b** when added at 20 phr (entry code: **4b-20**). Indeed, this entry could be considered the trade-off solution to reach a good decrease not only in the melting temperature and in the storage modulus of PHB but also a relatively low environmental impact, which is comparable with those of entries characterized by 10 phr of plasticizer content added (Figure 6B,C).

On the glass transition temperature being considered (Figure 6A), entry **4a-10** represents the best compromise between the *T*_g_ reduction and the associated environmental impact. Indeed, it leads to an intermediate *T*_g_ reduction with respect to the further plasticizers (**4b**–**e**) added at 20 phr, but it presents an environmental impact that is only second to that of entry **4b**-**10**.

The results reported in Table S49 and Figure 6 highlight that it is possible, and it should represent a common practice, to concurrently comprise the environmental performances together with the induced thermal and mechanical properties when different plasticizers are assessed.

## Conclusions

Five innovative bioplasticizers derived from levulinic acid, the latter obtained from residual *C. cardunculus* L. biomass, were completely assessed from an environmental perspective by applying the LCA methodology from the cradle (i.e., the agricultural crop) to the gate (i.e., the chemical synthesis of the ketal-diester derivatives).

Independently by the impact category considered, the ketal-diester derivative obtained by a stearic acid precursor (**4b**) resulted in the one characterized by the lowest environmental impacts followed by those derived from myristic, phenylacetic, benzoic, and isovaleric acids.

By investigating in more detail the life cycle phases of plasticizer **4b**, the major contributions to its overall impact are those associated with the electric energy needed during the three steps of synthesis, for which reaction times of 18 (first step) and 7 (second and third steps) h are needed.

During microwave-assisted acid-catalyzed hydrolysis and subsequent purification to obtain highly pure levulinic acid from exploded biomass, the most impacting contributions are those related to the purification by liquid–liquid extraction with 2-methyl THF.

Notably, the environmental impact of the exploded biomass accounts only for 0.2% of the impact of the obtained LA. Of this 0.2%, only 5.67% is due to the epigean residue obtained as the coproduct during the agricultural *C. cardunculus* L. crop production.

The LCA results were considered in conjunction with those related to the plasticizing performances of the ketal-diester derivatives (**4a**–**e**) when added at different phrs to the PHB biopolymer. In this way, it was possible to highlight that bioplasticizer **4b** when added at 20 phr could be considered a good compromise between its environmental performances and the decreases in *T*_m_ and *E*′ when added to PHB. By considering the sole decrease in *T*_g_, the trade-off alternative could be represented by plasticizer **4a** (i.e., derived from myristic acid) when added to PHB at 10 phr. The adoption of similar approaches, when assessing bioplasticizers, is highly desirable to contribute to find out the optimal trade-off solution in terms of both environmental and thermo-mechanical performances.

## Supporting Information Available

Agronomic data for 1 ha of *C. cardunculus* L. cultivated farmland (Table S1); complete inventories used to model *C. cardunculus* L. crop production and associated processes (Tables S2 and S3), residual biomass pretreatment (Tables S4–S10), the obtainment of LA (Tables S11–S20), the three-step synthesis of **4a**–**e** (Tables S21–S40); endpoint results for the production of **4a**–**e** (Table S41); single score results for the production of **4a**–**e** (Table S42); single score results for 1 g of **4b** (Table S43); single score results for precursor **3b** (Table S44); single score results for the obtainment of LA from exploded biomass (Table S45); single score results for the obtainment of exploded biomass (Table S46); single score results for 1 t of the epigean residue (Table S47); midpoint results of the sensitivity analysis referred to the synthesis of 1 g of bioplasticizer **4b**, in accordance with the three scenarios considered alternatively to the reference one (Table S48); reductions in *T*_g_, *T*_m_, and *E*′ induced by **4a**–**e** when added to PHB and their associated single score environmental impacts (Table S49); flowcharts summarizing the boundaries of the system studied (Figures S1–S4); relative percentage of the midpoint environmental impacts associated with 1 g of the five bioplasticizers **4a**–**e** (Figure S5); relative impact percentage associated with 1 g of the five bioplasticizers **4a**–**e**, calculated at the endpoint level (Figure S6); single score results associated with the production of 1 g of bioplasticizer **4b** (Figure S7); single score results associated with 5.008 g of exploded biomass (Figure S8); single score results associated with 1 t of the epigean residue (Figure S9); relative midpoint environmental impacts associated with the preparation of 1 g of the bioplasticizer **4b** in accordance with the three scenarios considered alternatively to the reference one (Figure S10) (PDF)
