# Supplementary material for: Innovative Bioplasticizers from Residual Cynara cardunculus L. Biomass-Derived Levulinic Acid and Their Environmental Impact Assessment by LCA Methodology
Source: ACS Sustain Chem Eng. 2023 Aug 1;11(32):12014–26. doi: 10.1021/acssuschemeng.3c02269 (PMC10428505; doi:10.1021/acssuschemeng.3c02269)
Supplement: Supplementary file 1 — sc3c02269_si_001.pdf [file sc3c02269_si_001.pdf]

SUPPORTING INFORMATION

Innovative bioplasticizers from residual *Cynara cardunculus* L. biomass-derived levulinic acid and their environmental impact assessment by LCA methodology

Chiara Ruini[1], Paolo Neri[1], Gianluca Cavalaglio[2], Valentina Coccia[3], Franco Cotana[3], Anna Maria Raspolli Galletti[4], Davide Morselli[5,6], Paola Fabbri[5,6], Anna Maria Ferrari[1,6,7], Roberto Rosa[1,6,7*]

[1]Dipartimento di Scienze e Metodi dell'Ingegneria, Università degli Studi di Modena e Reggio Emilia, via G. Amendola 2, 42122 Reggio Emilia, Italy

[2]Università Telematica Pegaso, Centro Direzionale Isola f2, 80143 Napoli, Italy

[3]Centro Interuniversitario di Ricerca sull'Inquinamento e sull'Ambiente "Mauro Felli", Centro di Ricerca sulle Biomasse, University of Perugia, via G. Duranti 63, 06125 Perugia, Italy

[4]Dipartimento di Chimica e Chimica Industriale, Università di Pisa, via G. Moruzzi 13, 56124 Pisa, Italy

[5]Dipartimento di Ingegneria Civile, Chimica, Ambientale e dei Materiali, Università di Bologna, via U. Terracini 28, 40131 Bologna, Italy

[6]Consorzio Interuniversitario Nazionale per Scienza e Tecnologia dei Materiali (INSTM), via Giusti 9, 50121 Firenze, Italy

[7]Centro Interdipartimentale En&Tech, Università degli Studi di Modena e Reggio Emilia, Tecnopolo di Reggio Emilia, Piazzale Europa 1, 42123, Reggio Emilia, Italy

*corresponding author: E-mail: roberto.rosa@unimore.it; Phone: +39 0522523558.

Number of pages: 90

Number of Tables: 49

Number of Figures: 10

TABLES

Table S1. Agronomic data referred to one hectare of farmland cultivated with *Cynara Cardunculus* L.

Agronomic data description		Amount (unit)
Seeds	*Cynara Carnduculus* L. seeds	1.5 (t)
	Humidity	8 (%)
	Nitrogen (on dry matter basis)	3 (%)
	Oil	25 (%)
Residual biomass	Epigean residue (as it is)	15 (t)
	Epigean residue (on dry matter basis)	13.5 (t)
	Epigean residue humidity	15 (%)
	C content in the epigean residue	36 (%)
	N content in the epigean residue	0.6 (%)
	PCI epigean residue (on dry matter basis)	16.4 (MJ/kg)
	Hypogeum residue (on dry matter basis)	3 (t)
	Hypogeum residue humidity	50 (%)
Necessary crop inputs	Seeds	0.46 (kg)
	Diesel	77.3 (l)
	18:46 (diammonium phosphate) - N	4.5 (kg)
	18:46 (diammonium phosphate) - P	147 (kg)
	N of chemical origin	57.5 (kg)
	N of biological origin (compost)	20 (kg)

Table S2. Contributions to the Life Cycle Inventory (LCI) for the production of 1p of HDPE bag used for containing and transport 50 kg of fertilizers. The dimensions of the bag were assumed to be 0.5x0.15x0.7 m^3 and the thickness was assumed to be 0.0005 m. The density of HDPE was considered 953 kg/m^3. The resulting weight of the bag was 0.5051 kg.

Description			Amount	Process data source
Input	Materials	HDPE	0.5175 kg	Ecoinvent v. 3.8[1]
	Processing	Forming	0.5175 kg	Ecoinvent v. 3.8[2]
	Transport	Transport of raw materials	51.75 kgkm	Ecoinvent v. 3.8[3]
Output	End of life	Recycling	0.0124 kg	Ecoinvent v. 3.8[4]
	End of life	Incineration	0.5051 kg	Ecoinvent v. 3.8[5]

[1]The Ecoinvent process used was: Polyethylene, high density, granulate {RoW}| production | APOS, U. A higher amount is needed to account for the loss during forming.
[2]The Ecoinvent process used was: Extrusion, plastic film {RoW}| extrusion, plastic film | APOS, U. 1 kg of this process leads to 0.976 kg of extruded plastic film.
[3]The Ecoinvent process used was: Transport, freight, lorry 16-32 metric ton, EURO6 {RoW}| transport, freight, lorry 16-32 metric ton, EURO6 | APOS, U. An average distance of 100 km was considered.
[4]Recycling of the surplus plastic lost during forming. The recycling process was modified by the authors. Particularly a new process was created with the inputs taken from the Ecoinvent database process of the Polypropylene recycling. However, the environmental loads were allocated to both the recycling process and the secondary recycled plastic with a contribution of 50 % each.
[5]The Ecoinvent process used was: Hazardous waste, for incineration {RoW}| treatment of hazardous waste, hazardous waste incineration, with energy recovery | APOS, U.

Table S3. Contributions to the Life Cycle Inventory (LCI) for the cultivation of 1 ha of farmland with *Cynara Cardunculus* L. crop, leading to 1.5 t of *Cynara Cardunculus* L. seeds and 15 t of epigean residue (as it is). Due to the multioutput character of the process an economic allocation between the main product (i.e., the seeds with an allocation percentage of 92.87%) and the co-product (i.e., the epigean residue with an allocation percentage of 7.13%) was employed. Particularly an average price for lignocellulosic biomasses of 0.035 €/kg and a price of 4.56 €/kg were considered for epigean residue and *Cynara cardunculus* L. seeds respectively.

Description			Amount	Process data source
Outputs	Avoided products	Nitrogen fertiliser	0.0315 t	Ecoinvent v. 3.8[1]
		Phosphorus fertiliser	0.018 t	Ecoinvent v. 3.8[2]
		Potassium fertiliser	0.027 t	Ecoinvent v. 3.8[3]
Inputs	Inputs from nature	Land occupation	5000 m^2y	SimaPro raw materials list[4]
		Land	10000 m^2	SimaPro raw materials

		transformation		list[5]
		Land transformation	10000 m^2	SimaPro raw materials list[6]
		Gross calorific value	26308.35 MJ	SimaPro raw materials list[7]
		CO_2	27720 kg	SimaPro raw materials list[8]
	Materials	Cynara Cardunculus L. seeds	0.46 kg	modeled from Ecoinvent v. 3.8 database sub processes as detailed in Table S2[9]
		Nitrogen from fertiliser	57.5 kg	Ecoinvent v. 3.8[10]
		Phosphorus from fertiliser	146.94 kg	Ecoinvent v. 3.8[11]
		Nitrogen from compost	20 kg	Ecoinvent v. 3.8[12]
	Equipment/plants	Bag	41.405 p	modeled from Ecoinvent v. 3.8 database sub processes as detailed in Table S1[13]
	Agricultural processes	Deep tillage	1 ha	Ecoinvent v. 3.8[14]
		Surface tillage	1 ha	Ecoinvent v. 3.8[15]
		Harrowing	1 ha	Ecoinvent v. 3.8[16]
		Sowing	1 ha	Ecoinvent v. 3.8[17]
		Fertilising	1 ha	Ecoinvent v. 3.8[18]
		Seeds harvesting	1 ha	Ecoinvent v. 3.8[19]
		Biomass harvesting	1 ha	Ecoinvent v. 3.8[20]
	Transport	Transport of the fertilizers	209115.356 kgkm	Ecoinvent v. 3.8[21]
Outputs	Emissions to air	CO_2, land transformation	1.078 t	SimaPro airborne emission substance list[22]
		CO_2, biogenic	3.96 t	SimaPro airborne emission substance list[23]
		Ammonia	3.76 kg	SimaPro airborne emission substance list[24]
		Nitrogen oxides	1.027 kg	SimaPro airborne emission substance list[25]
		Dinitrogen monoxide	4.888 kg	SimaPro airborne emission substance list[26]

Emissions to water	Nitrate in groundwater	102.925 kg	SimaPro waterbone emission substance list[27]
	Phosphate in river	0.239 kg	SimaPro waterbone emission substance list[28]
	Phosphate in groundwater	0.07 kg	SimaPro waterbone emission substance list[29]
	Phosphorus in river	1767 kg	SimaPro waterbone emission substance list[30]
	Cadmium in river	40.869 mg	SimaPro waterbone emission substance list[31]
	Cadmium in groundwater	45.777 mg	SimaPro waterbone emission substance list[32]
	Chromium in river	4275.66 mg	SimaPro waterbone emission substance list[33]
	Chromium in groundwater	20221.3 mg	SimaPro waterbone emission substance list[34]
	Copper in river	3362.125 mg	SimaPro waterbone emission substance list[35]
	Copper in groundwater	3237.482 mg	SimaPro waterbone emission substance list[36]
	Nickel in river	3398.969	SimaPro waterbone emission substance list[37]
	Lead in river	2989.819 mg	SimaPro waterbone emission substance list[38]
	Lead in groundwater	494.594 mg	SimaPro waterbone emission substance list[39]
	Zinc in river	5874.275 mg	SimaPro waterbone emission substance list[40]
	Zinc in groundwater	21012.299 mg	SimaPro waterbone emission substance list[41]
Emissions to soil	Carbon dioxide, to soil or biomass stock	3.96 t	SimaPro soil emission substance list[42]
	Cadmium	7439.989 mg	SimaPro soil emission substance list[43]
	Chromium	49165.957 mg	SimaPro soil emission substance list[44]
	Copper	-27171.195 mg	SimaPro soil emission substance list[45]
	Nickel	15905.066 mg	SimaPro soil emission substance list[46]
	Lead	82101.991 mg	SimaPro soil emission

			substance list[47]
	Zinc	101225.846 mg	SimaPro soil emission substance list[48]

[1]Amount of nitrogen saved as a consequence of the hypogeum residue decomposition. As its nitrogen content it was assumed the amount of nitrogen contained in the Ecoinvent database process Compost {GLO}|nutrient supply from compost| Conseq, U, i.e. 0.7%. The Ecoinvent process used was: Nitrogen fertilizer, as N {RER}| diammonium phosphate production | APOS, U.
[2]Amount of phosphorous saved as a consequence of the hypogeum residue decomposition. As its phosphorus content it was assumed the amount of phosphorus contained in the Ecoinvent database process Compost {GLO}|nutrient supply from compost| Conseq, U, i.e. 0.4%. The Ecoinvent process used was: Inorganic phosphorus fertiliser, as P2O5 {RER}| nutrient supply from diammonium phosphate | APOS, U.
[3]Amount of potassium saved as a consequence of the hypogeum residue decomposition. As its potassium content it was assumed the amount of potassium contained in the Ecoinvent database process Compost {GLO}|nutrient supply from compost| Conseq, U, i.e. 0.6%. The Ecoinvent process used was: Potassium chloride, as K2O {RER}| potassium chloride production | APOS, U.
[4]For the land occupation, 6 months were considered since for *Cynara Cardunculus* L. sowing starts in Spring while the biomass harvesting occurs at the end of the Summer season. The SimaPro raw material considered was Occupation, annual crop in the subcompartment land.
[5]The SimaPro raw material considered was Transformation, to annual crop in the subcompartment land.
[6]The SimaPro raw material considered was Transformation, from shrub land, sclerophyllous in the subcompartment land.
[7]This value was calculated starting from the value of 1.25 MJ referred to the production of 1 kg of Celery (i.e. Celery{GLO}| 675 production | APOS, U). The gross energy for Celery was calculated using following formula; gross energy (MJ) = crude protein (kg) * 23.04 (MJ/kg) + crude fat (kg) * 38.96 (MJ/kg) + crude fibre (kg) * 17.95 (MJ/kg) + carbohydrates (kg) * 17.17 (MJ/kg). The product composition of fruits and vegetables was extracted from the USDA National Nutrient Database for Standard Reference (http://ndb.nal.usda.gov/). Crude protein (g)= 6.9, crude fat (g)= 1.7, crude fibre (g)= 16, ash (g)= 7.5, water (g)= 954.3, carbohydrates (g)= 29.7. The gross energy per kg of DM is 18.26 MJ. The calculated gross energy per kg of reference product is thus 1.25 MJ.
This value was then multiplied by the mass of *Cynara Cardunculus* L. seeds, epigean and hypogeum residues.
The SimaPro raw material considered was Energy, gross calorific value, in biomass in the subcompartment biotic.
[8]Amount of CO_2 captured from the atmosphere by the *Cynara Cardunculus* L. biomass. This value was calculated by assuming the 36% of C content experimentally determined for epigean residue (as reported in Table S1) also for hypogeum residue and the seeds. The calculation performed is the following: 0.36*(mass of seeds (=1500 kg) + mass of epigean residue (= 15000 kg) + mass of hypogeum residue (=4500 kg))/atomic mass of carbon (= 12)*molecular mass of CO_2 (= 44) =27720 kg. This is in full agreement to what performed by Ecoinvent database in modelling agricultural production systems [1].
The SimaPro raw material considered was Carbon dioxide, in air.
[9]Amount of Cynara Cardunculus L. seeds used for seeding.
[10]Amount of Nitrogen deriving from the total amount of 18:46 diammonium phosphate fertilizer used (i.e., 319.44 kg). The Ecoinvent process used was: Inorganic nitrogen fertilizer, as N {RER}| nutrient supply from diammonium phosphate as N | APOS, U.
[11]Amount of Phosphorus deriving from the total amount of 18:46 diammonium phosphate fertilizer used (i.e., 319.44 kg). The Ecoinvent process used was: Inorganic phosphorus fertilizer, as P2O5 {RER}| nutrient supply from diammonium phosphate as N | APOS, U.
[12]Amount of Nitrogen deriving from the total amount of compost used. The Ecoinvent process used was: Organic nitrogen fertiliser, as N {GLO}| nutrient supply from compost | APOS, U. 1 kg of this process correspond to 87.54 kg of compost material.
[13]Number of 50 kg capacity HDPE bags for the transport of all the fertilizers. The indicated value was calculated by applying the formula 1/50*(319.44+20), where 319.44 is the total mass of 18:46 diammonium phosphate used for 1ha and 20 is the mass of compost used for 1 ha.
[14]The Ecoinvent process used was: Tillage, subsoiling, by subsoiler plow {RoW}| tillage, subsoiling, by subsoiler plow | APOS, U.
[15]The Ecoinvent process used was: Tillage, ploughing {RoW}| processing | APOS, U.
[16]The Ecoinvent process used was: Tillage, harrowing, by spring tine arrow {RoW}| processing | APOS, U.
[17]The Ecoinvent process used was: Sowing {RoW}| processing | APOS, U.
[18]The Ecoinvent process used was: Fertilising, by broadcaster {RoW}| processing | APOS, U.
[19]The Ecoinvent process used was: Harvesting, by complete harvester, ground crops {RoW}| harvesting, by complete harvester, ground crops | APOS, U.
[20]The Ecoinvent process used was: Haying, by rotary tedder {RoW}| processing | APOS, U.

[21]Transport of 18:46 diammonium phosphate and compost in the HDPE bags of 50 kg of capacity and 0.5051 kg of weight each. The Ecoinvent process used was: Transport, freight, lorry 16-32 metric ton, EURO6 {RoW}| transport, freight, lorry 16-32 metric ton, EURO6 | APOS, U. An average distance of 100 km was considered.
[22]This value represent the lacking absorption of CO_2 from the atmosphere, as a consequence of the land transformation from shrub land, sclerophyllous to the Cynara Cardunculus L. crop production. For the calculation it has been assumed the life time of the shrub land as equal to 10 years. Then, the number of sclerophyllous plants in 1 ha of land was calculated as ca. 4444 by considering an average distance between the plants of 1.5 m. For each sclerophyllous plant it has been considered the total weight comprising the trunk, the branches, and the roots. Particularly for the weight of the trunk it has been assumed an average diameter of 0.1 m, and average height of 2.5 m and the density of wood equal to 0.8 t/m^3. The overall weight of the trunks resulted 69.81 ton. The weight of the branches was assumed to be one third of the weight of the trunks, while the weight of the roots was assumed to be one half of the weight of the trunks.
The overall weight of the sclerophyllous plants resulted 314.16 t. The carbon dioxide potentially captured by this plant material was calculated by multiplying the overall weight by 0.5 that approximately corresponds to the average C content of biomasses. This value was then allocated to the 0.5 years of the *Cynara Cardunculus* L. crop lifetime. To this value it has been subtracted the CO_2 potentially captured by the overall *Cynara Cardunculus* L. biomass (i.e., 21 t), calculated by multiplying the 21 t by the C percentage contained in the epigen residue (i.e., 36%), by subsequently dividing by the atomic weight of C and finally multiplying by the molecular weight of carbon dioxide.
[23]Carbon dioxide re-released into air by the hypogeum. This value was calculated by assuming that 2/3 of the C content (i.e., 36%) of the biomass remaining in the soil (i.e., the 4.5 t of hypogeum residue) will be re-released into air. The formula used is the following: 2/3*0.36*4.5 t / atomic mass of carbon (= 12)*molecular mass of CO_2 (= 44) = 3.96 t [1].
[24]This is the ammonia released into the air deriving from NH_4^+ contained in the fertilizers used. This was calculated by assuming the emission factor for NH_3 from mineral fertilisers of 4%, as the one typical for a multinutrient fertilizer [1]. Thus, the total amount of N in the fertilisers used (i.e., 77.5 kg) was multiplied by 0.04, then divided by atomic weight of Nitrogen and finally multiplied by the molecular weight of ammonia.
[25]During denitrification processes in soils, NO_x may also be produced and emitted into air. These emissions were estimated from the emissions of N_2O by the formula $NO_x = 0.21 * N_2O$ [1], where N_2O (emission of kg N_2O/ha) = $44/28*(0.0125(N_{av}-14/17*NH_3+N_{cr}+0.6N_{bf})+0.01*14/17*NH_3+0.025*14/62*NO_3^-)$, with N_{av} = available nitrogen (kg N/ha)= 77.5, Ncr = nitrogen contained in the crop residues (kg N/ha) = 62.5 (the latter being the average between 10 kg/ha in wheat under both tillage systems and 115 kg/ha in clover GM under zero tillage [1, 2]), N_{bf} = nitrogen from biological N fixation (kg N/ha) = 105 (the latter being the average value as estimated by the quantity of N contained in the shoots of legumes, as reported in [3]), NH_3 = loss of nitrogen as ammonia emissions = 3.76 as calculated above (see note n° 24) and NO_3^- = loss of nitrogen in the form of nitrate (kg NO_3^-/ha) = 102.9 (the latter calculated by assuming a 30% of the N_{av} that transforms into NO_3^-).
[26]Calculated by the formula $N_2O = 44/28*(0.0125(N_{av}-14/17*NH_3+N_{cr}+0.6N_{bf}) + 0.01*14/17*NH_3+0.025*14/62*NO_3^-)$ [1]. For the meaning of the variables in the equation please refer to the above reported note n°25.
[27]Calculated considering the 30% of the available nitrogen released in groundwater as nitrate by the formula NO_3^- =(0.3*Nav/14.0067)*62.0067. For the meaning of the variables in the equation please refer to the above reported note n°25.
[28]Run-off of soluble phosphate to surface water. Calculated by multiplying the average quantity of P lost through run-off for a land use category which is 0.175 kg P/(ha*y) for open arable land [1], by the correction factor for fertilization with P (the latter calculated as 1+0.2/80*P_2O_5contained in the fertilizer used [1]).
[29]Leaching of soluble phosphate to ground water P_{gw}. Calculated by multiplying the average quantity of P leached to ground water for a land use category which is 0.07 kg P/(ha*y) for arable land [1] by the correction factor for fertilization by slurry (1+0.2/80*P_2O_5contained in the slurry) which resulted 1 since no slurry was employed.
[30]Erosion of soil particles containing phosphorus. Calculated by the formula $P_{er}=10000*S_{er}*P_{cs}*F_r*F_{erw}$ [1]. S_{er} represents the quantity of soil eroded (kg/(ha*y)) considered as 500 kg /(ha*y) as the average value indicated between those of 0-1 t/ha/y for most of the Italian Sardinia region in [4]. P_{cs} = 0.00095 kg/kg is the average value used for the P content in the top soil [1]. F_r represents the enrichment factor for P for which the average value of 1.86 was used, while F_{erw} is the average value of 0.2 used for the fraction of the eroded soil that reaches the river [1].
[31]Cd emissions through erosion. Calculated by the formula $M_{erosion,\,Cd}$ (mg)=$Cd_{soil}*B*a*f_{erosion}*A_{Cd}$. Cd_{soil} is the Cd content in mg per kg of soil and it was assumed 0.24 as indicated for arable land in [1]. B represents the quantity of soil eroded (kg/(ha*y)) considered as 500 kg /(ha*y) as the average value indicated between those of 0-1 t/ha/y for most of the Italian Sardinia region in [4]. a is instead the accumulation factor of 1.86 [1]. $F_{erosion}$ represents the erosion factor that was considered equal to 0.2 as average value [1]. Acd is the allocation factor for the share of agricultural inputs in the total inputs for Cd. A_{cd}=mass of Cd in the fertilisers used/(mass of Cd in the fertilisers used+mass of Cd from atmospheric deposition). The mass of Cd in the fertilisers used resulted 7587.196 mg while for the mass of Cd from atmospheric deposition the value of 700 mg/ha/y was used as suggested in [1].
[32]Leaching of Cd to the ground water. Calculated by multiplying the value of 50 mg Cd/ha/y [1, 5] by A_{cd} previously described in note n° 31.
[33]Cr emissions through erosion. Calculated by a similar formula as the one used for Cd reported in note n°31, with Cr_{soil}=24.1 as indicated for arable land [1] and A_{Cr}= 0.9538, calculated by considering the mass of Cr in the fertilisers = 75414.1 mg and the mass of Cr from atmospheric deposition =3650 mg/ha/y as suggested in [1].

[34]Leaching of Cr to the ground water. Calculated by multiplying the value of 21200 mg Cr/ha/y [1, 5] by A_{Cr} previously described in note n° 33.
[35]Cu emissions through erosion. Calculated by a similar formula as the one used for Cd reported in note n°31, with Cu_{soil}=20.1mg/kg as indicated for arable land [1] and A_{Cu}= 0.8993, calculated by considering the mass of Cu in the fertilisers = 21433.313 mg and the mass of Cu from atmospheric deposition =2400 mg/ha/y as suggested in [1].
[36]Leaching of Cu to the ground water. Calculated by multiplying the value of 3600 mg Cu/ha/y [1, 5] by A_{Cu} previously described in note n° 35.
[37]Ni emissions through erosion. Calculated by a similar formula as the one used for Cd reported in note n°31, with Ni_{soil}=23.0mg/kg as indicated for arable land [1] and A_{Ni}= 0.7945, calculated by considering the mass of Ni in the fertilisers = 21170.306 mg and the mass of Ni from atmospheric deposition =5475 mg/ha/y as suggested in [1].
[38]Pb emissions through erosion. Calculated by a similar formula as the one used for Cd reported in note n°30, with Pb_{soil}=19.5 mg/kg as indicated for arable land [1] and A_{Pb}= 0.8243, calculated by considering the mass of Pb in the fertilisers = 87745.232 mg and the mass of Pb from atmospheric deposition =18700 mg/ha/y as suggested in [1].
[39]Leaching of Pb to the ground water. Calculated by multiplying the value of 600 mg Pb/ha/y [1, 5] by A_{Pb} previously described in note n° 38.
[40]Zn emissions through erosion. Calculated by a similar formula as the one used for Cd reported in note n°30, with Zn_{soil}=49.6 mg/kg as indicated for arable land [1] and A_{Zn}= 0.6367, calculated by considering the mass of Zn in the fertilisers = 158455.056 mg and the mass of Zn from atmospheric deposition =90400 mg/ha/y as suggested in [1].
[41]Leaching of Zn to the ground water. Calculated by multiplying the value of 33000 mg Zn/ha/y [1, 5] by A_{Zn} previously described in note n° 40.
[42]Carbon dioxide released into soil by the hypogeum. Calculated by the formula: 1/3*Mass of hypogeum residue as it is*C fraction in the hypogeum residue/12*44, where the mass of hypogeum residue as it is = 4.5 t, and the carbon fraction in the hypogeum residue was assumed equal to the one of the epigean residue, i.e., 0.36. It is assumed that 1/3 of the carbon will return to the atmosphere.
[43]Cadmium released in soil and in the agricultural subcompartment. Calculated by the formula $M_{soil,\ Cd} = (\sum inputs_{Cd} - \sum outputs_{Cd}) * A_{Cd}$. For the calculation of the total input into the soil of Cd, the values previously reported in note n°31 for Cd in fertilisers (i.e., 7587.196 mg) and for Cd deposited from atmosphere (i.e., 700 mg), were added with Cd deriving from the biomass remaining on the soil (i.e., 14.702 mg, obtained by multiplying the average content of Cd for biomasses of 0.0049 mg/kg, by the mass of hypogeum and Cynara Cardunculus L. seeds used for the seeding). To this Cd input, it was subtracted the amount of Cd leached into the groundwater and eroded from the soil and released in surface water, together with the amount contained in the removed biomass (i.e., 80.85 mg, the latter obtained by multiplying the average content of Cd for biomasses of 0.0049 mg/kg, by the mass of the exported epigean residue and seeds). Details on the calculation of A_{Cd} are reported in note n°31.
[44]Chromium released in soil and in the agricultural subcompartment. Calculated by the formula $M_{soil,\ Cr} = (\sum inputs_{Cr} - \sum outputs_{Cr}) * A_{Cr}$. For the calculation of the total input into the soil of Cr, the values previously reported in note n°33 for Cr in fertilisers (i.e., 75414.1 mg) and for Cr deposited from atmosphere (i.e., 650 mg), were added with Cr deriving from the biomass remaining on the soil (i.e., 408.063 mg, obtained by multiplying the average content of Cr for biomasses of 0.136 mg/kg, by the mass of hypogeum and Cynara Cardunculus L. seeds used for the seeding). To this Cr input, it was subtracted the amount of Cr leached into the groundwater and eroded from the soil and released in surface water, together with the amount contained in the removed biomass (i.e., 2244 mg, the latter obtained by multiplying the average content of Cr for biomasses of 0.136 mg/kg, by the mass of the exported epigean residue and seeds). Details on the calculation of A_{Cr} are reported in note n°33.
[45]Copper released in soil and in the agricultural subcompartment. Calculated by the formula $M_{soil,\ Cu} = (\sum inputs_{Cu} - \sum outputs_{Cu}) * A_{Cu}$. For the calculation of the total input into the soil of Cu, the values previously reported in note n°35 for Cu in fertilisers (i.e., 21433.313 mg) and for Cu deposited from atmosphere (i.e., 2400 mg), were added with Cu deriving from the biomass remaining on the soil (i.e., 10381.592 mg, obtained by multiplying the average content of Cu for biomasses of 3.46 mg/kg, by the mass of hypogeum and Cynara Cardunculus L. seeds used for the seeding). To this Cu input, it was subtracted the amount of Cu leached into the groundwater and eroded from the soil and released in surface water, together with the amount contained in the removed biomass (i.e., 57090 mg, the latter obtained by multiplying the average content of Cu for biomasses of 3.46 mg/kg, by the mass of the exported epigean residue and seeds). Details on the calculation of A_{Cu} are reported in note n°35.
As in the present case, some of the values for emissions of heavy metals to the soil are negative. This means that more heavy metals are exported than imported. It must, however, be borne in mind that these heavy metals are transferred either to the water bodies or to the products harvested from the field [1].
[46]Nickel released in soil and in the agricultural subcompartment. Calculated by the formula $M_{soil,\ Ni} = (\sum inputs_{Ni} - \sum outputs_{Ni}) * A_{Ni}$. For the calculation of the total input into the soil of Ni, the values previously reported in note n°37 for Ni in fertilisers (i.e., 21170.306 mg) and for Ni deposited from atmosphere (i.e., 5475 mg), were added with Ni deriving from the biomass remaining on the soil (i.e., 522.080 mg, obtained by multiplying the average content of Ni for biomasses of 0.174 mg/kg, by the mass of hypogeum and Cynara Cardunculus L. seeds used for the seeding). To this Ni input, it was subtracted the amount of Ni leached into the groundwater and eroded from the soil and released in surface water, together with the amount contained in the removed biomass (i.e., 2871 mg, the latter obtained by

multiplying the average content of Ni for biomasses of 0.174 mg/kg, by the mass of the exported epigean residue and seeds). Details on the calculation of A_{Ni} are reported in note n°37.

[47]Lead released in soil and in the agricultural subcompartment. Calculated by the formula $M_{soil,\ Pb} = (\sum inputs_{Pb} - \sum outputs_{Pb}) * A_{Pb}$. For the calculation of the total input into the soil of Pb, the values previously reported in note n°38 for Pb in fertilisers (i.e., 87745.232 mg) and for Pb deposited from atmosphere (i.e., 18700 mg), were added with Pb deriving from the biomass remaining on the soil (i.e., 582.089 mg, obtained by multiplying the average content of Pb for biomasses of 0.194 mg/kg, by the mass of hypogeum and Cynara Cardunculus L. seeds used for the seeding). To this Pb input, it was subtracted the amount of Pb leached into the groundwater and eroded from the soil and released in surface water, together with the amount contained in the removed biomass (i.e., 3201 mg, the latter obtained by multiplying the average content of Pb for biomasses of 0.194 mg/kg, by the mass of the exported epigean residue and seeds). Details on the calculation of A_{Pb} are reported in note n°38.

[48]Zinc released in soil and in the agricultural subcompartment. Calculated by the formula $M_{soil,\ Zn} = (\sum inputs_{Zn} - \sum outputs_{Zn}) * A_{Zn}$. For the calculation of the total input into the soil of Zn, the values previously reported in note n°40 for Zn in fertilisers (i.e., 158455.056 mg) and for Zn deposited from atmosphere (i.e., 90400 mg), were added with Zn deriving from the biomass remaining on the soil (i.e., 10591.624 mg, obtained by multiplying the average content of Zn for biomasses of 3.53 mg/kg, by the mass of hypogeum and Cynara Cardunculus L. seeds used for the seeding). To this Zn input, it was subtracted the amount of Zn leached into the groundwater and eroded from the soil and released in surface water, together with the amount contained in the removed biomass (i.e., 58245 mg, the latter obtained by multiplying the average content of Zn for biomasses of 3.53 mg/kg, by the mass of the exported epigean residue and seeds). Details on the calculation of A_{Zn} are reported in note n°40.

Table S4. Contributions to the Life Cycle Inventory (LCI) for the production of 1 p of steel tank (for the acid impregnation of the biomass), with a weight of 0.6162 ton, a capacity of 7.2 m^3. The tank with dimensions of 3x2x1.20 m is endowed with a steel filter with dimensions of 3x2x0.002 m. The lifetime of the tank and the filter was assumed to be 30 years, during which it was assumed to be used for 8 h/day, for 5 days/week and for 48 weeks/year, thus for a total of 57600 h.

Description			Amount	Process data source
Input	Materials	Steel of the tank	0.6162 ton	Ecoinvent v. 3.8[1]
		Steel of the filter	0.0936 ton	Ecoinvent v. 3.8[2]
	Processing	Hot rolling	0.6162 ton	Ecoinvent v. 3.8[3]
		Welding	14.8 m	Ecoinvent v. 3.8[4]
		Deep drawing	0.0936 ton	Ecoinvent v. 3.8[5]
		Drilling	0.00936 ton	Ecoinvent v. 3.8[6]
	Transport	Transport of materials	70.98 tkm	Ecoinvent v. 3.8[7]
	End of life	Recycling	0.6162 ton	Ecoinvent v. 3.8[8]
		Recycling	0.0936 ton	Ecoinvent v. 3.8[8]

[1]Steel used for the production of the tank. The Ecoivent process used was: Steel, chromium steel 18/8 {RoW}| steel production, electric, chromium steel 18/8 | APOS, U.

[2]Steel used for the production of the filter. The Ecoivent process used was: Steel, chromium steel 18/8 {RoW}| steel production, electric, chromium steel 18/8 | APOS, U.

[3]Steel processing. The Ecoinvent process used was: Hot rolling, steel {RER}| processing | APOS, U.

[4]The Ecoinvent process used was: Welding, arc, steel {RoW}| processing | APOS, U.

[5]Process of deep drawing for the production for the filter. The Ecoinvent process used was: Deep drawing, steel, 650 kN press, automode {RoW}| deep drawing, steel, 650 kN press, automode | APOS, U.

[6]Drilling process to produce the holes in the filter. A 10% of the weight of the filter was assumed to have been removed by drilling. The Ecoivent process used was: Chromium steel removed by drilling, computer numerical controlled {RoW}| chromium steel drilling, computer numerical controlled | APOS, U.

[7]Transport of steel to the processing site. An average distance of 100 km was considered. The Ecoinvent process used was: Transport, freight, lorry 16-32 metric ton, EURO6 {RoW}| transport, freight, lorry 16-32 metric ton, EURO6 | APOS, U.

[8]The recycling process for steel was modified by the authors. A new multi-output process was created with the input of 1 kg of steel (the Ecoinvent process used was Steel, low-alloyed {RER}| steel production, electric, low-alloyed | APOS, U). The main product of the process created was the recycling treatment of 1.105 kg of steel, while the co-product was 1 kg of secondary steel. The environmental loads were allocated to both the recycling process and the secondary recycled steel obtained with a contribution of 50 % each. The process comprised also the end of life of the remaining scraps (the Ecoinvent process used was Scrap steel {CH}| treatment of, inert material landfill | APOS, U).

Table S5. Contributions to the Life Cycle Inventory (LCI) for the production of 1 p of the steam explosion reactor, with a weight of 40 kg, a capacity of 10 l and a power of 2 HP. Its lifetime was assumed to be 30 years, during which it was assumed to be used for 8 h/day, for 5 days/week and for 48 weeks/year, thus for a total of 57600 h.

Description			Amount	Process data source
Input	Materials	Steel	40 kg	Ecoinvent v. 3.8[1]
	Processing	Forming	40 kg	Ecoinvent v. 3.8[2]
	Transport	Transport of materials	4000 kgkm	Ecoinvent v. 3.8[3]
	End of life	Recycling	49.08 kg	Ecoinvent v. 3.8[4]

[1]Steel used for the production of the reactor. The Ecoivent process used was: Steel, chromium steel 18/8 {RoW}| steel production, electric, chromium steel 18/8 | APOS, U.
[2]Forming of the steel reactor. The Ecoivent process used was: Metal working, average for chromium steel product manufacturing {RoW}| processing | APOS, U. This process comprises an additional contribution of steel (i.e., 0.227 kg for 1 kg of formed steel).
[3]Transport of steel to the processing site. An average distance of 100 km was considered. The Ecoinvent process used was: Transport, freight, lorry 16-32 metric ton, EURO6 {RoW}| transport, freight, lorry 16-32 metric ton, EURO6 | APOS, U.
[4]The recycling process for steel was modified by the authors. A new multi-output process was created with the input of 1 kg of steel (the Ecoinvent process used was Steel, low-alloyed {RER}| steel production, electric, low-alloyed | APOS, U). The main product of the process created was the recycling treatment of 1.105 kg of steel, while the co-product was 1 kg of secondary steel. The environmental loads were allocated to both the recycling process and the secondary recycled steel obtained with a contribution of 50 % each. The process comprised also the end of life of the remaining scraps (the Ecoinvent process used was Scrap steel {CH}| treatment of, inert material landfill | APOS, U).

Table S6. Contributions to the Life Cycle Inventory (LCI) for the production of 1 p of the settling steel tank, with a weight of 0.6162 ton, a capacity of 7.2 m^3. The tank has dimensions of 3x2x1.20 m. The lifetime of the tank is assumed to be 30 years, during which it was assumed to be used for 8 h/day, for 5 days/week and for 48 weeks/year, thus for a total of 57600 h. The tank is used after the steam explosion process and allows to separate by decantation the exploded biomass from the liquid media.

Description			Amount	Process data source
Input	Materials	Steel of the tank	0.6162 ton	Ecoinvent v. 3.8[1]
	Processing	Hot rolling	0.6162 ton	Ecoinvent v. 3.8[2]
		Welding	14.8 m	Ecoinvent v. 3.8[3]
	Transport	Transport of materials	61.62 tkm	Ecoinvent v. 3.8[4]
	End of life	Recycling	0.6162 ton	Ecoinvent v. 3.8[5]

[1]Steel used for the production of the tank. The Ecoivent process used was: Steel, chromium steel 18/8 {RoW}| steel production, electric, chromium steel 18/8 | APOS, U.
[2]Processing of the steel tank. The Ecoinvent process used was: Hot rolling, steel {RER}| processing | APOS, U.
[3]Processing of the steel tank. The Ecoinvent process used was: Welding, arc, steel {RoW}| processing | APOS, U.

[4]Transport of steel to the processing site. An average distance of 100 km was considered. The Ecoinvent process used was: Transport, freight, lorry 16-32 metric ton, EURO6 {RoW}| transport, freight, lorry 16-32 metric ton, EURO6 | APOS, U.
[5]The recycling process for steel was modified by the authors. A new multi-output process was created with the input of 1 kg of steel (the Ecoinvent process used was Steel, low-alloyed {RER}| steel production, electric, low-alloyed | APOS, U). The main product of the process created was the recycling treatment of 1.105 kg of steel, while the co-product was 1 kg of secondary steel. The environmental loads were allocated to both the recycling process and the secondary recycled steel obtained with a contribution of 50 % each. The process comprised also the end of life of the remaining scraps (the Ecoinvent process used was Scrap steel {CH}| treatment of, inert material landfill | APOS, U).

Table S7. Contributions to the Life Cycle Inventory (LCI) for the production of 1 p of the press used to remove the liquid yet present in the fibers of the biomass. A generic plant composed of steel (75%), copper (5%) aluminium (10%) and HDPE (10%) for a total weight of 100 kg was considered. Its lifetime was assumed to be 30 years, during which it was assumed to be used for 8 h/day, for 5 days/week and for 48 weeks/year, thus for a total of 57600 h.

Description			Amount	Process data source
Input	Materials	Steel	75 kg	Ecoinvent v. 3.8[1]
		Copper	5 kg	Ecoinvent v. 3.8[2]
		Aluminium	10 kg	Ecoinvent v. 3.8[3]
		HDPE	10.03 kg	Ecoinvent v. 3.8[4]
	Processing	Forming of steel	75 kg	Ecoinvent v. 3.8[5]
		Forming of Cu	5 kg	Ecoinvent v. 3.8[6]
		Forming of Al	10	Ecoinvent v. 3.8[7]
		Forming of HDPE	10.03 kg	Ecoinvent v. 3.8[8]
	Transport	Transport of materials	10003.01 kgkm	Ecoinvent v. 3.8[9]
	End of life	Recycling of steel	92.025 kg	Ecoinvent v. 3.8[10]
		Recycling of Cu	5 kg	Ecoinvent v. 3.8[11]
		Recycling of Al	10 kg	Ecoinvent v. 3.8[12]
		Recycling of HDPE	10.03 kg	Ecoinvent v. 3.8[13]

[1]Steel used for the production of the general plant. The Ecoinvent process used was: Steel, chromium steel 18/8 {RoW}| steel production, electric, chromium steel 18/8 | APOS, U.
[2]Copper used for the production of the general plant. The Ecoinvent process used was: Copper, cathode {RER}| treatment of copper scrap by electrolytic refining | APOS, U.
[3]Aluminium used for the production of the general plant. The Ecoinvent process used was: Aluminium, primary, ingot {RoW}| production | APOS, U.
[4]HDPE used for the production of the general plant, comprising also the amount lost in the subsequent forming procedure. The Ecoinvent process used was: Polyethylene, high density, granulate {RER}| production | APOS, U.
[5]Steel processing. The Ecoinvent process used was: Metal working, average for chromium steel product manufacturing {RoW}| processing | APOS, U. This process comprises a further input of steel of 0.227 kg per functional unit.
[6]Copper processing. The Ecoinvent process used was: Wire drawing, copper {RER}| processing | APOS, U.
[7]Aluminium processing. The Ecoinvent process used was: Sheet rolling, aluminium {RER}| processing | APOS, U.
[8]HDPE processing. The Ecoinvent process used was: Blow moulding {RER}| blow moulding | APOS, U. 1 kg of this process leads to 0.997 kg of blow moulded plastic.
[9]Transport of the materials constituting the general plant. An average distance of 100 km was considered. The Ecoinvent process used was: Transport, freight, lorry 16-32 metric ton, EURO6 {RER}| transport, freight, lorry 16-32 metric ton, EURO6 | APOS, U.
[10]The recycling process for steel was modified by the authors. A new multi-output process was created with the input of 1 kg of steel (the Ecoinvent process used was Steel, low-alloyed {RER}| steel production, electric, low-alloyed | APOS, U). The main product of the process created was the recycling treatment of 1.105 kg of steel, while the co-product was 1

kg of secondary steel. The environmental loads were allocated to both the recycling process and the secondary recycled steel obtained with a contribution of 50 % each. The process comprised also the end of life of the remaining scraps (the Ecoinvent process used was Scrap steel {CH}| treatment of, inert material landfill | APOS, U).

[11]The recycling process for copper was modified by the authors. A new multi-output process was created with the input of 1 kg of copper (the Ecoinvent process used was Copper, cathode {GLO}| treatment of non-Fe-Co-metals, from used Li-ion battery, hydrometallurgical processing | APOS, U). The main product of the process created was the recycling treatment of 1 kg of copper, while the co-product was 1 kg of secondary copper. The environmental loads were allocated to both the recycling process and the secondary recycled copper obtained with a contribution of 50 % each.

[12]The recycling process for aluminium was modified by the authors. A new multi-output process was created with the input of 1 kg of aluminium (the Ecoinvent process used was Aluminium, wrought alloy {RER}| treatment of aluminium scrap, new, at remelter | APOS, U). The main product of the process created was the recycling treatment of 1 kg of aluminium, while the co-product was 1 kg of secondary aluminium. The environmental loads were allocated to both the recycling process and the secondary recycled aluminium obtained with a contribution of 50 % each.

[13]End of life of HDPE. The recycling process was modified by the authors. Particularly a new process was created with the inputs taken from the Ecoinvent database process of the Polypropylene recycling. However, the environmental loads were allocated to both the recycling process and the secondary recycled plastic with a contribution of 50 % each.

Table S8. Contributions to the Life Cycle Inventory (LCI) for the production of 1p of the aspiration system used during pre-treatment/steam explosion process. The aspiration system, with an air flow rate of 250 m^3/h was considered composed of an electric ventilator, a hood, a chimney and a channel. The lifetime of the electric ventilator was assumed to be 18000 h, while the one of the aspiration system was assumed to be 79750 h.

Description			Amount	Process data source
Input	Materials	Steel	25.237 kg	Ecoinvent v. 3.8[1]
		Copper	8.412 kg	Ecoinvent v. 3.8[2]
		Aluminum	4.206 kg	Ecoinvent v. 3.8[3]
		PVC	4.206 kg	Ecoinvent v. 3.8[4]
		Steel	20.932 kg	Ecoinvent v. 3.8[5]
		Steel	206.093 kg	Ecoinvent v. 3.8[6]
		Steel	115.927 kg	Ecoinvent v. 3.8[7]
	Processing	Forming steel	25.237 kg	Ecoinvent v. 3.8[8]
		Forming copper	8.412 kg	Ecoinvent v. 3.8[9]
		Forming aluminum	4.206 kg	Ecoinvent v. 3.8[10]
		Forming PVC	4.206 kg	Ecoinvent v. 3.8[11]
		Forming steel	342.952 kg	Ecoinvent v. 3.8[12]
	Transport	Transport of raw materials	38.502 tkm	Ecoinvent v. 3.8[13]
Output	End of life	Recycling of steel	25.237 kg	Ecoinvent v. 3.8[14]
		Recycling of copper	8.412 kg	Ecoinvent v. 3.8[15]
		Recycling of Aluminum	4.206 kg	Ecoinvent v. 3.8[16]

		Recycling of plastic	4.206 kg	Ecoinvent v. 3.8[17]
		Recycling of steel	342.952 kg	Ecoinvent v. 3.8[14]

[1]60% of the weight of the electric ventilator was supposed to be steel. The Ecoinvent process used was: Reinforcing steel {RoW}| production | APOS, U.
[2]20% of the weight of the electric ventilator was supposed to be copper. The Ecoinvent process used was: Copper, anode {RoW}| smelting of copper concentrate, sulfide ore | APOS, U.
[3]10% of the weight of the electric ventilator was supposed to be aluminum. The Ecoinvent process used was: Aluminium, cast alloy {RoW}| treatment of aluminium scrap, new, at refiner | APOS, U.
[4]10% of the weight of the electric ventilator was supposed to be polyvinylchloride. The Ecoinvent process used was: Polyvinylchloride, bulk polymerised {RoW}| polyvinylchloride production, bulk polymerisation | APOS, U.
[5]Steel of the hood. The Ecoinvent process used was: Reinforcing steel {RoW}| production | APOS, U.
[6]Steel of the channel connecting the electric ventilator to the hood. The Ecoinvent process used was: Reinforcing steel {RoW}| production | APOS, U.
[7]Steel of the channel connecting the electric ventilator to the exit. The Ecoinvent process used was: Reinforcing steel {RoW}| production | APOS, U.
[8]Forming of the steel composing the electric ventilator. The Ecoinvent process used was: Section bar rolling, steel {RoW}| processing | APOS, U.
[9]Forming of the copper composing the electric ventilator. The Ecoinvent process used was: Wire drawing, copper {RoW}| processing | APOS, U.
[10]Forming of the aluminum composing the electric ventilator. The Ecoinvent process used was: Section bar extrusion, aluminium {RoW}| processing | APOS, U.
[11]Forming of the polyvinylchloride composing the electric ventilator. The Ecoinvent process used was: Blow moulding {RoW}| blow moulding | APOS, U.
[12]Forming of the steel channel. The Ecoinvent process used was: Section bar rolling, steel {RoW}| processing | APOS, U.
[13]The Ecoinvent process used was: Transport, freight, lorry 16-32 metric ton, EURO6 {RoW}| transport, freight, lorry 16-32 metric ton, EURO6 | APOS, U. An average distance of 100 km was considered.
[14]The recycling process for steel was modified by the authors. Particularly a new multi-output process was created with the input of 1 kg of steel (the Ecoinvent process used was Steel, low-alloyed {RER}| steel production, electric, low-alloyed | APOS, U). The main product of the process created was the recycling treatment of 1.105 kg of steel, while the co-product was 1 kg of secondary steel. The environmental loads were allocated to both the recycling process and the secondary recycled steel obtained with a contribution of 50 % each. The process comprised also the end of life of the remaining scraps (the Ecoinvent process used was Scrap steel {CH}| treatment of, inert material landfill | APOS, U).
[15]The recycling process for copper was modified by the authors. Particularly a new process was created with the inputs taken from the Ecoinvent database process named Copper, cathode {RoW}| treatment of metaln part of electronic scrap, in copper, anode, by electrolytic refining | APOS, U. However, the environmental loads were allocated to both the recycling process and the secondary recycled copper with a contribution of 50 % each.
[16]The recycling process for aluminum was modified by the authors. Particularly a new process was created with the inputs taken from the Ecoinvent database process named Aluminium, wrought alloy {RER}| treatment of aluminium scrap, new, at remelter | APOS, U. However, the environmental loads were allocated to both the recycling process and the secondary recycled copper with a contribution of 50 % each.
[17]The recycling process for plastic was modified by the authors. Particularly a new process was created with the inputs taken from the Ecoinvent database process of the Polypropylene recycling. However, the environmental loads were allocated to both the recycling process and the secondary recycled plastic with a contribution of 50 % each.

Table S9. Contributions to the Life Cycle Inventory (LCI) for the production of 1p of the activated carbon air filter used pre-treatment/steam explosion process. The activated carbon air filter was considered for a chemical hood with an air flow rate of 250 m^3/h, and the velocity of filter crossing of the air was assumed to be 0.5 m/s. The lifetime of the filter was assumed to be 20000 h, while that of activated carbon was assumed to be 2400 h.

Description			Amount	Process data source
Input	Materials	Activated	54.398 kg	Ecoinvent v. 3.8[1]

		carbon		
		Steel	12.683 kg	Ecoinvent v. 3.8[2]
	Processing	Forming steel	12.683 kg	Ecoinvent v. 3.8[3]
	Transport	Transport of raw materials	6708.095 kgkm	Ecoinvent v. 3.8[4]
Output	End of life	Recycling	12.683 kg	Ecoinvent v. 3.8[5]
		Treatment of spent activated carbon	54.398 kg	Ecoinvent v. 3.8[6]

[1]A single load of activated carbon weights 6.528 kg. The reported amount accounts also for the activated carbon needed during the whole filter lifetime. The Ecoinvent process used was: Activated carbon, granular {RoW}| activated carbon production, granular from hard coal | APOS, U.
[2]Weight of the filter container. The Ecoinvent process used was: Reinforcing steel {RER}| production | APOS, U.
[3]The Ecoinvent process used was: Sheet rolling, steel {RER}| processing | APOS, U.
[4]The Ecoinvent process used was: Transport, freight, lorry 16-32 metric ton, EURO6 {RoW}| transport, freight, lorry 16-32 metric ton, EURO6 | APOS, U. An average distance of 100 km was considered.
[5]The recycling process for steel was modified by the authors. Particularly a new multi-output process was created with the input of 1 kg of steel (the Ecoinvent process used was Steel, low-alloyed {RER}| steel production, electric, low-alloyed | APOS, U). The main product of the process created was the recycling treatment of 1.105 kg of steel, while the co-product was 1 kg of secondary steel. The environmental loads were allocated to both the recycling process and the secondary recycled steel obtained with a contribution of 50 % each. The process comprised also the end of life of the remaining scraps (the Ecoinvent process used was Scrap steel {CH}| treatment of, inert material landfill | APOS, U).
[6]The Ecoinvent process used was: Spent activated carbon, granular {RoW}| treatment of spent activated carbon, granular from hard coal, reactivation | APOS, U.

Table S10. Contribution to the Life Cycle inventory (LCI) for the production of 1437.5 kg of exploded Biomass. This is the pre-treatment process of the Cynara Cardunculus biomass, starting with the chipping of the biomass, its overnight acid impregnation, filtration, steam explosion process, pressing and washing.

Description			Amount	Process Data Source
Input	Materials	Epigean residue	575 kg	Modelled from Ecoinvent v 3.8 database sub process as detailed in Table S3
		H_2SO_4	26.95 kg	Ecoinvent v. 3.8[1]
		Tap Water	1769.68 kg	Ecoinvent v. 3.8[2]
		Tap Water	2392.09 kg	Ecoinvent v. 3.8[3]
		Steam	513.82 kg	Ecoinvent v. 3.8[4]
	Equipment/plants	Steam Explosion reactor	$6.5*10^{-3}$ p	Modelled from Ecoinvent v 3.8 database sub process as detailed in Table S5[5]
		Impregnation tank	$1.4*10^{-4}$ p	Modelled from Ecoinvent v 3.8 database sub process as detailed in Table S4[6]

		Decantation tank	$7.09*10^{-6}$ p	Modelled from Ecoinvent v 3.8 database sub process as detailed in Table S6[7]
		Press 1	$6.16*10^{-6}$ p	Modelled from Ecoinvent v 3.8 database sub process as detailed in Table S7[8]
		Press 2	$2.49*10^{-5}$ p	Modelled from Ecoinvent v 3.8 database sub process as detailed in Table S7[9]
		Storage tank	$1.29*10^{-2}$ p	Modelled from Ecoinvent v 3.8 database sub process as detailed in Table S6[10]
		Aspiration system	$4.91*10^{-3}$ p	Modelled from Ecoinvent v 3.8 database sub process as detailed in Table S8[11]
		Activated carbon air filter	$1.96*10^{-6}$ p	Modelled from Ecoinvent v 3.8 database sub process as detailed in Table S9[12]
	Processing	Chipping	575 kg	Ecoinvent v. 3.8[13]
	Transports	Transport of the epigeal residue	57500 kgkm	Ecoinvent v. 3.8[14]
		Transport of H_2SO_4	2695 kgkm	Ecoinvent v. 3.8[15]
		Transport of large equipment	814.25 kgkm	Ecoinvent v. 3.8[16]
		Transport of large equipment	26.01 kgkm	Ecoinvent v. 3.8[17]
		Transport of large equipment	0.31 kgkm	Ecoinvent v. 3.8[18]
		Transport of large equipment	0.817 tkm	Ecoinvent v. 3.8[19]
	Energy	Electric energy	561.91 kWh	Ecoinvent v. 3.8[20]
		Electric energy	7.09 kWh	Ecoinvent v. 3.8[21]
		Electric energy	249973 kJ	Ecoinvent v. 3.8[22]
		Electric energy	28.70 kWh	Ecoinvent v. 3.8[23]
		Electric energy	32.81 kWh	Ecoinvent v. 3.8[24]
Output	Avoided products	H_2SO_4	18.86 kg	Ecoinvent v. 3.8[25]
		Tap Water	1238.73 kg	Ecoinvent v. 3.8[26]
	Emissions	H_2SO_4	$7.77*10^{-10}$ kg	SimaPro airborne emission substance list[27]
		H_2O	0.75 kg	SimaPro airborne

				emission substance list[28]
		H_2O	$4.07*10^{-4}$ kg	SimaPro airborne emission substance list[29]
		H_2O	$1.98*10^{-3}$ kg	SimaPro airborne emission substance list[30]
	End of life	Spent solvent mixture	560.53 kg	Ecoinvent v 3.8[31]
		Wastewater treatment	1.43 m^3	Ecoinvent v 3.8[32]

[1]Sulfuric acid used during the overnight acid impregnation. The Ecoinvent process used was: Sulfuric acid {RER}| production | APOS, U.

[2]Tap water used for the preparation of the sulfuric acid solution use during the overnight acid impregnation. The Ecoinvent process used was: Tap water {Europe without Switzerland} | tap water production, conventional treatment | APOS, U.

[3]Tap water used for washing the exploded biomass after the first pressing step. The Ecoinvent process used was: Tap water {Europe without Switzerland} | tap water production, conventional treatment | APOS, U.

[4]The Ecoinvent process used was: Steam, in chemical industry {RoW}| production | APOS, U.

[5]The reported value was calculated by the formula: 1/(57600 h*2.77 kg/h)*1038.95 kg, where 57600 is the lifetime of the reactor, 2.77 is the productivity of the reactor, while 1038.95 is the mass to be treated, excluding the emission of water and H_2SO_4, and including the amounts of water and H_2SO_4 used during filtration and impregnation and not recovered (i.e., 30%).

[6]The reported value was calculated by the formula: 1/7.2m^3*4.188m^3/57600h*14h, where 7.2 m^3 is the volume of the modeled impregnation tank, 4.188m^3 is the volume to be contained by the tank (including the biomass, the water and the H_2SO_4), 57600 is the lifetime of the tank, and 14h is the overnight impregnation time.

[7]The reported value was calculated by the formula: 1/7.2m^3*0.408m^3/57600h*1h, where 7.2 m^3 is the volume of the modeled impregnation tank, 0.408m^3 is the volume to be contained by the decantation tank after filtration (including the biomass, the water and the H_2SO_4), 57600 is the lifetime of the tank, and 1h is the decantation time.

[8]The reported value was calculated by the formula: 1/(57600h*2000kg/h)*709.224 kg, where 57600 h is the lifetime of the modeled press, 2000 kg/h is the pressing capacity of the modeled press and 709.224 kg is the mass to be pressed by press 1.

[9]The reported value was calculated by the formula: 1/(57600h*2000kg/h)*2870.31 kg, where 57600 h is the lifetime of the modeled press, 2000 kg/h is the pressing capacity of the modeled press and 2870.31 kg is the mass to be pressed by press 2. This latter value accounts also for the water used for washing after first pressing.

[10]The reported value was calculated by the formula: 1/57600h*24h/day*31day, where 57600 is the lifetime of the modeled tank, and the storage was considered to be performed for 1 month.

[11]The reported value was calculated by the formula 1/79750h*391.43h, where 79750 h is the lifetime of the modeled aspiration system and 391.43 h is the overall time necessary to the operations.

[12]The reported value was calculated by the formula 1/20000h*391.43h, where 20000 h is the lifetime of the modeled activated carbon air filter and 391.43 h is the overall time necessary to the operations.

[13]This is the first step of the pre-treatment process which includes the shredding of the biomass using a stationary electric chipper. It is assumed that the chipper has a capacity to process 1 ton of material per hour. The Ecoinvent process used was: Wood chipping, industrial residual wood, stationary electric chipper {RER}| processing | APOS, U.

[14]Transport of the epigeal residue of Cynara Cardunculus from the cultivation site to the laboratory. The average distance chosen is 100 km. The Ecoinvent process used was: Transport, freight, lorry 3.5-7.5 metric ton, EURO6 {RER}| transport, freight, lorry 3.5-7.5 metric ton, EURO6 | APOS, U.

[15]Transport of H_2SO_4 from the supplier to the laboratory. An average distance of 100 km was chosen. The Ecoivent process used was: Transport, freight, lorry 3.5-7.5 metric ton, EURO6 {RER}| transport, freight, lorry 3.5-7.5 metric ton, EURO6 | APOS, U.

[16]Transport for large equipment. An average distance of 100 km was chosen. The Ecoinvent process used was: Transport, freight, lorry 16-32 metric ton, EURO6 {RER}| transport, freight, lorry 16-32 metric ton, EURO6 | APOS, U.

[17]Transport of large equipment. An average distance of 100 km was chosen. The Ecoinvent process used was: Transport, freight, lorry 16-32 metric ton, EURO6 {RER}| transport, freight, lorry 16-32 metric ton, EURO6 | APOS, U.

[18]Transport of large equipment. An average distance of 100 km was chosen. The Ecoinvent process used was: Transport, freight, lorry 16-32 metric ton, EURO6 {RER}| transport, freight, lorry 16-32 metric ton, EURO6 | APOS, U.

[19]Transport of large equipment. An average distance of 100 km was chosen. The Ecoinvent process used was: Transport, freight, lorry 16-32 metric ton, EURO6 {RER}| transport, freight, lorry 16-32 metric ton, EURO6 | APOS, U.

[20]Electric energy used to heat up the steam explosion reactor at 165°C and for maintain this temperature for 10 minutes. The electric energy for the heating was calculated a power*mass/productivity, considering the total mass in the reactor, power of 1.5 kW and 2.77 kg/h as the reactor's productivity. The Ecoinvent process used was: Electricity, low voltage {IT}| electricity voltage transformation from medium to low voltage | APOS, U.
[21]Electric energy used during the first pressing of the exploded biomass. The Ecoinvent process used was: Electricity, low voltage {IT}| electricity voltage transformation from medium to low voltage | APOS, U.
[22]Electric energy used to heat up to 50°C the water used for biomass washing between the first and the second pressing. It is calculated using $m*Cp*\Delta T$, using 4.18 KJ/kg°C as water specifi heat. The Ecoinvent process used was: Electricity, low voltage {IT}| electricity voltage transformation from medium to low voltage | APOS, U.
[23]Electric energy used during the second pressing of the biomass. The Ecoinvet process used was: Electricity, low voltage {IT}| electricity voltage transformation from medium to low voltage | APOS, U.
[24]Electric energy necessary to the use of the aspiration system for the whole synthesis time, i.e. 391,42 h (considering the overnight acid impregnation and the following step of decantation, biomass pressing and washing). The power was calculated by considering the air flow rate of 250 m^3/h, a total load loss of 110.8076 kg/m^2, and an efficiency of 99%. The Ecoinvent process used was: Electricity, low voltage {IT}| electricity voltage transformation from medium to low voltage | APOS, U.
[25]It is estimated that the 70% of the sulfuric acid could be recovered. The Ecoinvent process used was: Sulfuric acid {RER}| production | APOS, U.
[26]Tap water which is recovered. The Ecoinvent process used was: Tap water {Europe without Switzerland}| tap water production, conventional treatment | APOS, U.
[27]Amount of Sulfuric Acid released into the atmosphere, as calculated by the formula reported in equation 1 of the main manuscript.
[28]Amount of water released into the atmosphere after the biomass is chipped and left to dry at room temperature. 15% of its moisture is considered to be emitted. Emission calculated using the formula reported in equation 1 of the main manuscript.
[29]Amount of water, used to impregnate the biomass, released into the atmosphere. It is calculated using the formula reported in equation 1 of the main manuscript.
[30]Amount of water, used after the first and the second press, released into the atmosphere. It is calculated using the formula reported in equation 1 of the main manuscript.
[31]End of life treatment for all the waste of the decantation and the first pressing process. The Ecoinvent process used was: Spent solvent mixture {RoW}| treatment of spent solvent mixture, hazardous waste incineration, with energy recovery | APOS, U.
[32]Wastewater, average {Europe without Switzerland}| treatment of wastewater, average, capacity 1E9l/year | APOS, U.

Table S11. Contribution to the Life Cycle inventory (LCI) for the production of 1 p of a 100% borosilicate glass round bottom flask, with a weight of 130 g. Its lifetime was assumed to be 15 years, during which it was assumed to be used for 8 h/day, for 5 days/week and for 48 weeks/year, thus for a total of 28800 h.

Description			Amount	Process data source
Input	Materials	Borosilicate glass	130 g	Ecoinvent v. 3.8[1]
	Transport	Transport of materials	13 kgkm	Ecoinvent v. 3.8[2]
Output	End of life	Landfilling	130 g	Ecoinvent v. 3.8[3]

[1]The Ecoinvent process used was: Glass tube, borosilicate {RoW}| production | APOS, U.
[2]Transport for the borosilicate glass. An average distance of 100 km was considered. The Ecoinvent process used was: Transport, freight, lorry 3.5-7.5 metric ton, EURO6 {RER}| transport, freight, lorry 3.5-7.5 metric ton, EURO6 | APOS, U.
[3]Borosilicate glass is not recycled, therefore for its end of life it is treated like and inert waste and delivered to landfill. The Ecoinvet process used was: Inert waste, for final disposal {RoW}| treatment of inert waste, inert material landfill | APOS, U.

Table S12. Contribution to the Life Cycle inventory (LCI) for the production of 1 p of a 100% borosilicate glass Vigreux Column with a weight of 300 g. Its lifetime was assumed to be 15 years, during which it was assumed to be used for 8 h/day, for 5 days/week and for 48 weeks/year, thus for a total of 28800 h.

Description			Amount	Process data source
Input	Materials	Borosilicate glass	130 g	Ecoinvent v. 3.8[1]
	Transport	Transport of materials	13 kgkm	Ecoinvent v. 3.8[2]
Output	End of life	Landfilling		Ecoinvent v. 3.8[3]

[1]The Ecoinvent process used was: Glass tube, borosilicate {RoW}| production | APOS, U.
[2]Transport for the borosilicate glass. An average distance of 100 km was considered. The Ecoinvent process used was: Transport, freight, lorry 3.5-7.5 metric ton, EURO6 {RER}| transport, freight, lorry 3.5-7.5 metric ton, EURO6 | APOS, U.
[3]Borosilicate glass is not recycled, therefore for its end of life it is treated like and inert waste and delivered to landfill. The Ecoinvet process used was: Inert waste, for final disposal {RoW}| treatment of inert waste, inert material landfill | APOS, U.

Table S13. Contributions to the Life Cycle Inventory (LCI) for the production of 1p of the silicone oil container. Its lifetime was assumed to be 30 years, during which it was assumed to be used for 8 h/day, for 5 days/week and for 48 weeks/year, thus for a total of 57600 h.

Description			Amount	Process data source
Input	Materials	Steel	0.8 kg	Ecoinvent v. 3.8[1]
	Processing	Forming	0.8 kg	Ecoinvent v. 3.8[2]
	Transport	Transport of raw materials	80 kgkm	Ecoinvent v. 3.8[3]
Output	End of life	Recycling	0.8 kg	Ecoinvent v. 3.8[4]

[1]The Ecoinvent process used was: Steel, chromium steel 18/8 {RoW}| steel production, electric, chromium steel 18/8 | APOS, U.
[2]The Ecoinvent process used was: Deep drawing, steel, 10000 kN press, automode {RoW}| deep drawing, steel, 10000 kN press, automode | APOS, U.
[3]The Ecoinvent process used was: Transport, freight, lorry 16-32 metric ton, EURO6 {RoW}| transport, freight, lorry 16-32 metric ton, EURO6 | APOS, U. An average distance of 100 km was considered.
[4]The recycling process for steel was modified by the authors. Particularly a new multi-output process was created with the input of 1 kg of steel (the Ecoinvent process used was Steel, low-alloyed {RER}| steel production, electric, low-alloyed | APOS, U). The main product of the process created was the recycling treatment of 1.105 kg of steel, while the co-product was 1 kg of secondary steel. The environmental loads were allocated to both the recycling process and the secondary recycled steel obtained with a contribution of 50 % each. The process comprised also the end of life of the remaining scraps (the Ecoinvent process used was Scrap steel {CH}| treatment of, inert material landfill | APOS, U).

Table S14. Contributions to the Life Cycle Inventory (LCI) for the production of 1p of stir bar with the weight of 1.806 g. Its lifetime was assumed to be 100000 h.

Description			Amount	Process data source
Input	Materials	Steel	1.7879 g	Ecoinvent v. 3.8[1]
		Teflon	0.01806 g	Ecoinvent v. 3.8[2]
	Processing	Forming	1.7879 g	Ecoinvent v. 3.8[3]
	Transport	Transport of raw materials	0.1806 kgkm	Ecoinvent v. 3.8[4]
		Transport of equipment	0.1806 kgkm	Ecoinvent v. 3.8[5]
	End of life	Recycling	1.806 g	Ecoinvent v. 3.8[6]

[1]The weight of steel was supposed to be the 90% of the weight of the whole stir bar. The Ecoinvent process used was: Steel, chromium steel 18/8, hot rolled {RER}| production | APOS, U.
[2]The weight of Teflon coating was supposed to be the 10% of the weight of the whole stir bar. The Ecoinvent process used was: Tetrafluoroethylene film, on glass {RER}| production | APOS, U.
[3]Forming of the steel constituting the stir bar. The Ecoinvent process used was: Impact extrusion of steel, warm, 3 strokes {RER}| processing | APOS, U.
[4]The Ecoinvent process used was: Transport, freight, lorry 16-32 metric ton, EURO6 {RER}| transport, freight, lorry 16-32 metric ton, EURO6 | APOS, U. An average distance of 100 km was considered.
[5]Transport of the stir bar to its end of life treatment. The Ecoinvent process used was: Transport, freight, lorry 16-32 metric ton, EURO6 {RER}| transport, freight, lorry 16-32 metric ton, EURO6 | APOS, U. An average distance of 100 km was considered.
[6]Recycling of the stir bar. The recycling process for steel was modified by the authors. Particularly a new multi-output process was created with the input of 1 kg of steel (the Ecoinvent process used was Steel, low-alloyed {RER}| steel production, electric, low-alloyed | APOS, U). The main product of the process created was the recycling treatment of 1.105 kg of steel, while the co-product was 1 kg of secondary steel. The environmental loads were allocated to both the recycling process and the secondary recycled steel obtained with a contribution of 50 % each. The process comprised also the end of life of the remaining scraps (the Ecoinvent process used was Scrap steel {CH}| treatment of, inert material landfill | APOS, U).

Table S15. Contributions to the Life Cycle Inventory (LCI) for the production of 1p of the magnetic stirrer, with a weight of 2.8 kg and a power of 500W. Its lifetime was assumed to be 50000 h.

Description			Amount	Process data source
Input	Materials	Reinforcing Steel	1123.07 g	Ecoinvent v. 3.8[1]
		HDPE	980 g	Ecoinvent v. 3.8[2]
		Steel	420 g	Ecoinvent v. 3.8[3]
		Copper	280 g	Ecoinvent v. 3.8[4]
	Processing	Hot Rolling	1123.07 g	Ecoinvent v. 3.8[5]
		Blow Moulding	980 g	Ecoinvent v. 3.8[6]
		Wire Drawing (steel)	420 g	Ecoinvent v. 3.8[7]
		Wire drawing (copper)	280 g	Ecoinvent v. 3.8[8]
	Transport	Transport of raw materials	280.31 kgkm	Ecoinvent v. 3.8[9]
Output	End of life	Waste treatment	2.8 kg	Ecoinvent v. 3.8[10]

[1]The Ecoinvent process used was: Reinforcing steel {Europe without Austria}| reinforcing steel production | APOS, U.
[2]The Ecoinvent process used was: Polyethylene, high density, granulate {RER}| production | APOS, U.
[3]The Ecoinvent process used was: Steel, chromium steel 18/8 {RER}| steel production, electric, chromium steel 18/8 | APOS, U.
[4]The Ecoinvent process used was: Copper {RER}| production, primary | APOS, U.
[5]The Ecoinvent process used was: Hot rolling, steel {RoW}| processing | APOS, U.
[6]The Ecoinvent process used was: Blow moulding {RER}| blow moulding | APOS, U.
[7]The Ecoinvent process used was: Wire drawing, steel {RER}| processing | APOS, U.
[8]The Ecoinvent process used was: Wire drawing, copper {RoW}| processing | APOS, U.
[9]Transport of raw materials. An average distance of 100 km was chosen. The Ecoinvent process used was: Transport, freight, lorry 16-32 metric ton, EURO6 {RER}| transport, freight, lorry 16-32 metric ton, EURO6 | APOS, U
[10]The Ecoinvent process used was: Waste electric and electronic equipment {GLO}| treatment of, shredding | APOS, U.

Table S16. Contributions to the Life Cycle Inventory (LCI) for the production of 1p of Vacuum Pump with a weight of 25 kg and a power of 750W. Its lifetime was assumed to be 10 years, during which it was assumed to be used for 8 h/day, for 5 days/week and for 48 weeks/year, thus for a total of 19200 h.

Description			Amount	Process data source
Input	Materials	Steel	15 kg	Ecoinvent v. 3.8[1]
		Aluminium	5 kg	Ecoinvent v. 3.8[2]
		HDPE	1.25 kg	Ecoinvent v. 3.8[3]
		Copper	3.75 kg	Ecoinvent v. 3.8[4]
		Lubricating Oil	34.8 kg	Ecoinvent v. 3.8[5]
	Processing	Impact Extrusion	5 kg	Ecoinvent v. 3.8[6]
		Blow Moulding	1.25 kg	Ecoinvent v. 3.8[7]
		Sheet Rolling	15 kg	Ecoinvent v. 3.8[8]
		Wire drawing (copper)	3.75 kg	Ecoinvent v. 3.8[9]
	Transport	Transport of raw materials	2500 kgkm	Ecoinvent v. 3.8[10]
Output	End of life	Steel Recycling	15 kg	Ecoinvent v. 3.8[11]
		Aluminium Recycling	5 kg	Ecoinvent v. 3.8[12]
		Copper Recycling	3.75 kg	Ecoinvent v. 3.8[13]
		Plastic Recycling	1.25 kg	Ecoinvent v. 3.8[14]
		Waste mineral oil	34.8 kg	Ecoinvent v. 3.8[15]

[1]The Ecoinvent process used was: Steel, chromium steel 18/8 {RER}| steel production, electric, chromium steel 18/8 | APOS, U.

[2]The Ecoinvent process used was: Aluminium, cast alloy {RER}| treatment of aluminium scrap, new, at refiner | APOS, U

[3]The Ecoinvent process used was: Polyethylene, high density, granulate {RER}| production | APOS, U

[4]The Ecoinvent process used was: Copper {RER}| production, primary | APOS, U.

[5]The Ecoinvent process used was: Lubricating oil {RER}| production | APOS, U.

[6]The Ecoinvent process used was: Impact extrusion of aluminium, 3 strokes {RER}| processing | APOS, U.

[7]The Ecoinvent process used was: Blow moulding {RER}| blow moulding | APOS, U.

[8]The Ecoinvent process used was: Sheet rolling, chromium steel {RER}| processing | APOS, U.

[9]The Ecoinvent process used was: Wire drawing, copper {RoW}| processing | APOS, U.

[10]Transport of raw materials. An average distance of 100 km was chosen. The Ecoinvent process used was: Transport, freight, lorry >32 metric ton, EURO3 {RER}| transport, freight, lorry >32 metric ton, EURO3 | APOS, U

[11] The recycling process for steel was modified by the authors. A new multi-output process was created with the input of 1 kg of steel (the Ecoinvent process used was Steel, low-alloyed {RER}| steel production, electric, low-alloyed | APOS, U). The main product of the process created was the recycling treatment of 1.105 kg of steel, while the co-product was 1 kg of secondary steel. The environmental loads were allocated to both the recycling process and the secondary recycled steel obtained with a contribution of 50 % each. The process comprised also the end of life of the remaining scraps (the Ecoinvent process used was Scrap steel {CH}| treatment of, inert material landfill | APOS, U).

[12]The recycling process for aluminium was modified by the authors. A new multi-output process was created with the input of 1 kg of aluminium (the Ecoinvent process used was Aluminium, wrought alloy {RER}| treatment of aluminium scrap, new, at remelter | APOS, U). The main product of the process created was the recycling treatment of 1 kg of steel, and the co-product was 1 kg of secondary aluminium. The environmental loads were allocated to both the recycling process and the secondary recycled aluminium obtained with a contribution of 50 % each

[13] The recycling process for copper was modified by the authors. A new multi-output process was created with the input of 1 kg of copper (the Ecoinvent process used was Copper, cathode {RoW}| treatment of metal part of electronics scrap, in copper, anode, by electrolytic refining | APOS, U). The main product of the process created was the recycling treatment of 1 kg of copper, and the co-product was 1 kg of secondary aluminium. The environmental loads were allocated to both the recycling process and the secondary recycled copper obtained with a contribution of 50 % each.

[14] The recycling process for plastic was modified by the authors. A new multi-output process was created with the input of 1 kg of plastics. The main product of the process created was the recycling treatment of 1 kg of plastics, and the co-product was 0.97 kg of secondary aluminium. The environmental loads were allocated to both the recycling process and the secondary recycled plastic obtained with a contribution of 50 % each.

[15]The Ecoinvent process used was: Waste mineral oil {Europe without Switzerland}| treatment of waste mineral oil, hazardous waste incineration, with energy recovery | APOS, U.

Table S17. Contributions to the Life Cycle Inventory (LCI) for the production of 1p of Reactor with a weight of 0.1 ton, used during the synthesis of 2-methyltetrahydrofuran. Its lifetime was assumed to be 30 years during which it was assumed to be used for 8 h/day, for 5 days/week and for 48 weeks/year, thus for a total of 57600 h. It has a power of 1.7 kW [6].

Description			Amount	Process data source
Input	Materials	Steel	75 kg	Ecoinvent v. 3.8[1]
		Aluminium	10 kg	Ecoinvent v. 3.8[2]
		HDPE	10.03 kg	Ecoinvent v. 3.8[3]
		Copper	5 kg	Ecoinvent v. 3.8[4]
	Processing	Metal working	75 kg	Ecoinvent v. 3.8[6]
		Blow Moulding	10.03 kg	Ecoinvent v. 3.8[7]
		Sheet Rolling	10 kg	Ecoinvent v. 3.8[8]
		Wire drawing (copper)	5 kg	Ecoinvent v. 3.8[9]
	Transport	Transport of raw materials	10003.01kgkm	Ecoinvent v. 3.8[10]
Output	End of life	Steel Recycling	92.05 kg	Ecoinvent v. 3.8[11]
		Aluminium Recycling	10 kg	Ecoinvent v. 3.8[12]
		Copper Recycling	5 kg	Ecoinvent v. 3.8[13]
		Plastic Recycling	10.03 kg	Ecoinvent v. 3.8[14]

[1]The Ecoinvent process used was: Steel, chromium steel 18/8 {RER}| steel production, electric, chromium steel 18/8 | APOS, U.
[2]The Ecoinvent process used was: Aluminium, primary, ingot {RoW}| production | APOS, U.
[3]The Ecoinvent process used was: Polyethylene, high density, granulate {RER}| production | APOS, U
[4]The Ecoinvent process used was: Copper, cathode {RER}| treatment of copper scrap by electrolytic refining | APOS, U.
[5]The Ecoinvent process used was: Lubricating oil {RER}| production | APOS, U.
[6]The Ecoinvent process used was: Metal working, average for chromium steel product manufacturing {RoW}| processing | APOS, U.
[7]The Ecoinvent process used was: Blow moulding {RER}| blow moulding | APOS, U.
[8]The Ecoinvent process used was: Sheet rolling, chromium steel {RER}| processing | APOS, U.
[9]The Ecoinvent process used was: Wire drawing, copper {RoW}| processing | APOS, U.
[10]Transport of raw materials. An average distance of 100 km was chosen. The Ecoinvent process used was: Transport, freight, lorry 16-32 metric ton, EURO6 {RER}| transport, freight, lorry 16-32 metric ton, EURO6 | APOS, U.
[11] The recycling process for steel was modified by the authors. A new multi-output process was created with the input of 1 kg of steel (the Ecoinvent process used was Steel, low-alloyed {RER}| steel production, electric, low-alloyed | APOS, U). The main product of the process created was the recycling treatment of 1.105 kg of steel, while the co-product was 1 kg of secondary steel. The environmental loads were allocated to both the recycling process and the secondary recycled steel obtained with a contribution of 50 % each. The process comprised also the end of life of the remaining scraps (the Ecoinvent process used was Scrap steel {CH}| treatment of, inert material landfill | APOS, U).
[12]The recycling process for aluminium was modified by the authors. A new multi-output process was created with the input of 1 kg of aluminium (the Ecoinvent process used was Aluminium, wrought alloy {RER}| treatment of aluminium scrap, new, at remelter | APOS, U). The main product of the process created was the recycling treatment of 1 kg of steel, and the co-product was 1 kg of secondary aluminium. The environmental loads were allocated to both the recycling process and the secondary recycled aluminium obtained with a contribution of 50 % each
[13] The recycling process for copper was modified by the authors. A new multi-output process was created with the input of 1 kg of copper (the Ecoinvent process used was Copper, cathode {RoW}| treatment of metal part of electronics scrap, in copper, anode, by electrolytic refining | APOS, U). The main product of the process created was the recycling treatment

of 1 kg of copper, and the co-product was 1 kg of secondary aluminium. The environmental loads were allocated to both the recycling process and the secondary recycled copper obtained with a contribution of 50 % each.

[14] The recycling process for plastic was modified by the authors. A new multi-output process was created with the input of 1 kg of plastics. The main product of the process created was the recycling treatment of 1 kg of plastics, and the co-product was 0.97 kg of secondary aluminium. The environmental loads were allocated to both the recycling process and the secondary recycled plastic obtained with a contribution of 50 % each.

[15]The Ecoinvent process used was: Waste mineral oil {Europe without Switzerland}| treatment of waste mineral oil, hazardous waste incineration, with energy recovery | APOS, U.

Table S18. Contribution to the Life Cycle Inventory (LCI) for the production of 0.63 kg of 2-methyltetrahydrofuran, according to [6, 7]. This solvent is used during the purification of levulinic acid.

Description			Amount	Process Data Source
Input	Materials	Levulinic Acid	1 kg	Modelled from Ecoinvent v 3.8 database sub process as detailed in Table S20
		H_2	0.1 kg	Ecoinvent v. 3.8[1]
		Palladium	$5*10^{-4}$ kg	Ecoinvent v. 3.8[2]
		Rhenium	$5*10^{-4}$ kg	Ecoinvent v. 3.8[3]
		Activated Carbon	$9*10^{-4}$ kg	Ecoinvent v. 3.8[4]
	Equipment/plants	Aspiration system	$7.52*10^{-5}$ p	Modelled from Ecoinvent v 3.8 database sub process as detailed in Table S8[5]
		Activated carbon air filter	$3*10^{-4}$ p	Modelled from Ecoinvent v 3.8 database sub process as detailed in Table S9[6]
		Reactor	$1.042*10^{-4}$ p	Modelled from Ecoinvent v 3.8 database sub process as detailed in Table S17[7]
	Transports	Transport of reagents	110.1 kgkm	Ecoinvent v. 3.8[8]
		Transport of large equipment	0.0136 tkm	Ecoinvent v. 3.8[9]
	Energy	Electric energy	10.2 kWh	Ecoinvent v. 3.8[10]
		Electric energy	65409.40 J	Ecoinvent v. 3.8[11]
		Electric energy	248.69 kJ	Ecoinvent v. 3.8[12]
		Electric energy	0.503 kWh	Ecoinvent v. 3.8[13]
Output	Avoided product	H_2	0.05 kg	Ecoinvent v. 3.8[14]
	End of life	Spent solvent mixture	0.419 kg	Ecoinvent v. 3.8[15]

[1]The Ecoinvent process used was: Hydrogen, gaseous {Europe without Switzerland}| hydrogen production, gaseous, petroleum refinery operation | APOS, U.

[2]The catalyst used for the reaction is a 5%Re-5%Pd catalyst. The amount of the two metals was calculated as 0.1% of the principal reagent (in this case Levulinic Acid) and then inserted as input from nature as suggested in [8]. The Ecoinvent process used was: Palladium in ground from nature. Palladium used as catalyst.

[3]The catalyst used for the reaction is a 5%Re-5%Pd catalyst. The amount of the two metals was calculated as 0.1% of the principal reagent (in this case Levulinic Acid) and then insert as input from nature as suggested in [8]. The Ecoinvent process used was: Rhenium in ground from nature. Rhenium used as catalyst.
[4]This is the support use for the 5%Pd-5%Re catalyst. It was considered as 90% of the total catalyst. The Ecoinvent process used was: Activated carbon, granular {RER}| activated carbon production, granular from hard coal | APOS, U
[5]The reported value was calculated by the following formula: 1/79750h*6h, where 79750 h is the lifetime of the modeled aspiration system, and 6 h is the reaction time.
[6]The reported value was calculated by the following formula: 1/20000h*6h, where 20000 h is the lifetime of the modeled activated carbon air filter, and 6 h is the reaction time.
[7]The reported value was calculated by the following formula: 1/57600h*6h, where 57600 h is the lifetime of the modeled reactor, and 6 h is the reaction time.
[8]Transport for all of the reagents. An average distance of 100 km was considered. The Ecoinvent process used was: Transport, freight, lorry 3.5-7.5 metric ton, EURO6 {RER}| transport, freight, lorry 3.5-7.5 metric ton, EURO6 | APOS, U.
[9]Transport foe the large equipment. The Ecoinvent process used was: Transport, freight, lorry 16-32 metric ton, EURO6 {RER}| transport, freight, lorry 16-32 metric ton, EURO6 | APOS, U.
[10]Electric energy consumed by the reactor during the six hours of the reaction. It was determined as power of the reactor per time (considering the six hours). The Ecoinvent process used was: Electricity, low voltage {IT}| electricity voltage transformation from medium to low voltage | APOS, U.
[11]Electric energy necessary to bring the 2-methyltetrahydrofuran to its boiling point. It was determined using the equation $m*Cp*\Delta T$, using 162 J/molK as specific heat for the 2-methyltretrahydrofuran. The Ecoinvent process used was: Electricity, low voltage {IT}| electricity voltage transformation from medium to low voltage | APOS, U.
[12]Electric energy necessary to evaporate the final product. It was determined using the equation $mol*\Delta Hvap$, using 34kJ/mol as the vaporization enthalpy of 2-methyltetrahydrofuran. The Ecoinvent process used was: Electricity, low voltage {IT}| electricity voltage transformation from medium to low voltage | APOS, U.
[13]Electric energy consumed by the aspiration system during the reaction (six hours). The Ecoinvent process used was: Electricity, low voltage {IT}| electricity voltage transformation from medium to low voltage | APOS, U.
[14]Part of the hydrogen used is recovered. The Ecoinvent process used was: Hydrogen, gaseous {Europe without Switzerland}| hydrogen production, gaseous, petroleum refinery operation | APOS, U.
[15]Spent solvent mixture {Europe without Switzerland}| treatment of spent solvent mixture, hazardous waste incineration, with energy recovery | APOS, U.

Table S19. Contribution to the Life Cycle Inventory for the production of 1p of a Microwave reactor CEM Discover used during the extraction of levulinic acid from the exploded biomass, with a weight of 17.3 kg and a power of 0.3 kW. The materials, processing, packaging and energies for the reactor have been taken from [9]. In order to refer those data to a different MW reactor (with a different weight) a mass based allocation was performed. Its lifetime was assumed to be 25 years.

Description			Amount	Process Data Source
Input	Materials	Steel	11.491 kg	Ecoinvent v. 3.8[1]
		Aluminium	1.009 kg	Ecoinvent v. 3.8[2]
		Brass	0.0326 kg	Ecoinvent v. 3.8[3]
		Ferrite	0.4571 kg	Ecoinvent v. 3.8[4]
		Borosilicate Glass	1.859 kg	Ecoinvent v. 3.8[5]
		Ceramic materials	0.1094 kg	Ecoinvent v. 3.8[6]
		Copper	1.053 kg	Ecoinvent v. 3.8[7]
		PVC	0.2644 kg	Ecoinvent v. 3.8[8]

		Nylon 6	0.0653 kg	Ecoinvent v. 3.8[9]
		PET	0.2726 kg	Ecoinvent v. 3.8[10]
		Polystyrene	0.03917 kg	Ecoinvent v. 3.8[11]
		Polyoxymethylene	0.01795 kg	Ecoinvent v. 3.8[12]
		ABS Copolymer	0.3934 kg	Ecoinvent v. 3.8[13]
		Tin	0.00294 kg	Ecoinvent v. 3.8[14]
		Lead	0.00196 kg	Ecoinvent v. 3.8[15]
		Gold	$2.45*10^{-5}$ kg	Ecoinvent v. 3.8[16]
		Nickel	0.000979 kg	Ecoinvent v. 3.8[17]
		Silver	$9.79*10^{-5}$ kg	Ecoinvent v. 3.8[18]
		Zinc	0.000979 kg	Ecoinvent v. 3.8[19]
		Palladium	$9.793*10^{-6}$ kg	Ecoinvent v. 3.8[20]
		Folding boxboard carton	1.464 kg	Ecoinvent v. 3.8[21]
		Polystyrene foam	0.2693 kg	Ecoinvent v. 3.8[22]
		LDPE	0.0196 kg	Ecoinvent v. 3.8[23]
		Tap water	32.91 kg	Ecoinvent v. 3.8[24]
	Transport	Transport	1882.21 kg	Ecoinvent v. 3.8[25]
		Transport	1905 kg	Ecoinvent v. 3.8[26]
	Energy	Electric energy	102.34 MJ	Ecoinvent v. 3.8[27]
		Electric energy	59.15 MJ	Ecoinvent v. 3.8[28]
		Heat	103.15 MJ	Ecoinvent v. 3.8[29]
Output	Recycling	Steel Recycling	4.47 kg	Ecoinvent v. 3.83[30]
		Aluminium Recycling	4.47 kg	Ecoinvent v. 3.8[31]
		Copper Recycling	4.47 kg	Ecoinvent v. 3.8[32]

	Plastic Recycling	0.332 kg	Ecoinvent v. 3.8[33]
	Plastic Recycling	0.613 kg	Ecoinvent v. 3.8[34]
	Paper Recycling	0.613 kg	Ecoinvent v. 3.8[35]
End of life	Inert material landfill	3.60 kg	Ecoinvent v. 3.8[36]
	Municipal waste incineration	0.3708 kg	Ecoinvent v. 3.8[37]
	Municipal waste incineration	0.10255 kg	Ecoinvent v. 3.8[38]

[1]The Ecoinvent process used was: Steel, chromium steel 18/8 {RER}| steel production, electric, chromium steel 18/8 | APOS, U.
[2] The Ecoinvent process used was: Aluminium, cast alloy {RER}| treatment of aluminium scrap, new, at refiner | APOS, U.
[3]The Ecoinvet process used was: Brass {RoW}| production | APOS, U.
[4]The Ecoinvent process used was: Ferrite {GLO}| production | APOS, U
[5]The Ecoinvent process used was: Glass tube, borosilicate {RoW}| production | APOS, U.
[6]The Ecoinvent process used was: Ceramics ETH U.
[7]The Ecoinvet process used was: Copper, cathode {RER}| treatment of copper scrap by electrolytic refining | APOS, U.
[8]The Ecoinvent process used was: Polyvinylchloride, bulk polymerised {RER}| polyvinylchloride production, bulk polymerisation | APOS, U.
[9]The Ecoinvent process used was: Nylon 6 {RER}| production | APOS, U.
[10]The Ecoinvent process used was: Polyethylene terephthalate, granulate, amorphous {RER}| production | APOS, U.
[11]The Ecoinvent process used was: Polystyrene, general purpose {RER}| production | APOS, U.
[12]The Ecoinvent process used was: Polyoxymethylene (POM)/EU-27.
[13]The Ecoinvent process used was: Acrylonitrile-butadiene-styrene copolymer {RER}| production | APOS, U.
[14]The Ecoinvent process used was: Tin {RoW}| production | APOS, U.
[15]The Ecoinvent process used was: Lead {RER}| treatment of scrap acid battery, remelting | APOS, U.
[16]The Ecoinvent process used was: Gold {RoW}| gold refinery operation | APOS, U.
[17]The Ecoinvent process used was: Nickel, class 1 {RoW}| treatment of metal part of electronics scrap, in copper, anode, by electrolytic refining | APOS, U.
[18]the Ecoinvent process used was: Silver {RoW}| primary zinc production from concentrate | APOS, U.
[19]The Ecoinvent process used was: Zinc {RoW}| primary production from concentrate | APOS, U.
[20]The Ecoinvent process used was: Palladium {RER}| treatment of automobile catalyst | APOS, U.
[21]Packaging material. The Ecoinvent process used was: Folding boxboard carton {RER}| folding boxboard carton production | APOS, U.
[22]Packaging material. The Ecoinvent process use was: Polystyrene foam slab {RER}| production | APOS, U.
[23]Packaging material. The Ecoinvent process used was: Packaging film, low density polyethylene {RER}| production | APOS, U.
[24]Tap water used for packaging and assembly. The Ecoinvent process used was: Tap water {Europe without Switzerland}| tap water production, conventional treatment | APOS, U.
[25]Transport of the materials used for the microwave reactor. An average distance of 100 km was considered. The Ecoinvent process used was: Transport, freight, lorry 16-32 metric ton, EURO6 {RER}| transport, freight, lorry 16-32 metric ton, EURO6 | APOS, U.
[26]Transports of the different packaging material. An average distance of 100 km was considered. The Ecoinvent process used was: Transport, freight, lorry 3.5-7.5 metric ton, EURO6 {RER}| transport, freight, lorry 3.5-7.5 metric ton, EURO6 | APOS, U.
[27]Electric energy necessary for the production of the Microwave CEM Discover Reactor. The Ecoinvent process used was: Electricity, low voltage {IT}| electricity voltage transformation from medium to low voltage | APOS, U.
[28]Electric energy necessary for the production of the packaging. The Ecoinvent process used was: Electricity, low voltage {IT}| electricity voltage transformation from medium to low voltage | APOS, U.
[29]Heat use for the production of the Microwave CEM Discover Reactor. The Ecoinvent process used was: Heat, central or small-scale, natural gas {Europe without Switzerland}| heat production, natural gas, at boiler atmospheric non-modulating <100kW | APOS, U.
[30] The recycling process for steel was modified by the authors. A new multi-output process was created with the input of 1 kg of steel (the Ecoinvent process used was Steel, low-alloyed {RER}| steel production, electric, low-alloyed | APOS, U). The main product of the process created was the recycling treatment of 1.105 kg of steel, while the co-product was 1

kg of secondary steel. The environmental loads were allocated to both the recycling process and the secondary recycled steel obtained with a contribution of 50 % each. The process comprised also the end of life of the remaining scraps (the Ecoinvent process used was Scrap steel {CH}| treatment of, inert material landfill | APOS, U).
[31]The recycling process for aluminium was modified by the authors. A new multi-output process was created with the input of 1 kg of aluminium (the Ecoinvent process used was Aluminium, wrought alloy {RER}| treatment of aluminium scrap, new, at remelter | APOS, U). The main product of the process created was the recycling treatment of 1 kg of steel, and the co-product was 1 kg of secondary aluminium. The environmental loads were allocated to both the recycling process and the secondary recycled aluminium obtained with a contribution of 50 % each.
[32]The recycling process for copper was modified by the authors. A new multi-output process was created with the input of 1 kg of copper (the Ecoinvent process used was Copper, cathode {RoW}| treatment of metal part of electronics scrap, in copper, anode, by electrolytic refining | APOS, U). The main product of the process created was the recycling treatment of 1 kg of copper, and the co-product was 1 kg of secondary aluminium. The environmental loads were allocated to both the recycling process and the secondary recycled copper obtained with a contribution of 50 % each.
[33] This is the recycling of the plastics used to produce the Microwave CEM Discover Reactor. The recycling process for plastic was modified by the authors. A new multi-output process was created with the input of 1 kg of plastics. The main product of the process created was the recycling treatment of 1 kg of plastics, and the co-product was 0.97 kg of secondary aluminium. The environmental loads were allocated to both the recycling process and the secondary recycled plastic obtained with a contribution of 50 % each.
[34]This is the recycling of the plastic used as packaging. The recycling process for plastic was modified by the authors. A new multi-output process was created with the input of 1 kg of plastics. The main product of the process created was the recycling treatment of 1 kg of plastics, and the co-product was 0.97 kg of secondary aluminium. The environmental loads were allocated to both the recycling process and the secondary recycled plastic obtained with a contribution of 50 % each.
[35]This is the recycling of paper used as packaging. The recycling process for paper was modified by the authors. A new multi-output process was created with the input of 1.9 kg of paper (the Ecoinvent process used was: Sulfate pulp {RER}| production, elementary chlorine free bleached | Alloc Def, U (senza legno, pulpwood e wood ash)). The main product of the process created was the recycling treatment of 1.9 kg of paper, and the co-product was 1 kg of secondary paper. The environmental loads were allocated to both the recycling process and the secondary recycled paper obtained with a contribution of 50 % each.
[36]The 18.92% of the microwave CEM Discover Reactor weight is disposed of in landfill. In the value reported above is considered also the weight of the packaging of the CEM Reactor. The Ecoinvent process used was: Inert waste, for final disposal {RoW}| treatment of inert waste, inert material landfill | APOS, U.
[37]The 36% of the plastic contained in the microwave CEM Discover reactor is incinerated. The Ecoinvent procecess used was: Residue from mechanical treatment, industrial device {RoW}| treatment of, municipal waste incineration | APOS, U.
[38]The 5.86% of plastic and paper contained in the packaging is incinerated. The Ecoinvent process used was: Residue from mechanical treatment, industrial device {RoW}| treatment of, municipal waste incineration | APOS, U.

Table S20. Contributions for the Life Cycle Inventory (LCI) for the extraction of 1.058 g of Levulinic Acid from the exploded biomass of *Cynara Cardunculus* L. The extraction was conducted using an acid-catalyzed hydrolysis assisted by microwave (MW) irradiation. After the extraction the purification of levulinic acid was performed using 2-methytetrahydrofuran in a continuous liquid-liquid extraction followed by distillation.

Description		Amount	Process Data Source
Input Materials			
	H_2O	19.57 g	Ecoinvent v 3.8[1]
	HCl 37%	0.35 g	Ecoinvent v 3.8[2]
	Exploded biomass	4.98 g	Modelled from Ecoinvent v 3.8 database sub process as detailed in Table S10
	2-Methyltetrahydrofuran	51.24 g	Modelled from Ecoinvent v 3.8 database sub process as detailed in Table S18
	Silicon Oil	0.0145 kg	Ecoinvent v 3.8[3]

	Equipment/ plants	Round bottom flask	1.74 *10^{-4} p	Modelled from Ecoinvent v 3.8 database sub process as detailed in Table S11[4]
		Magnetic stir bar	4 *10^{-5} p	Modelled from Ecoinvent v 3.8 database sub process as detailed in Table S14[5]
		Magnetic stirrer/heater	5*10^{-4} p	Modelled from Ecoinvent v 3.8 database sub process as detailed in Table S15[6]
		Vigreux Column	3.47*10^{-5} p	Modelled from Ecoinvent v 3.8 database sub process as detailed in Table S12[7]
		Silicon Oil container	1.74*10^{-5} p	Modelled from Ecoinvent v 3.8 database sub process as detailed in Table S13[8]
		Vacuum pump	5.21*10^{-5} p	Modelled from Ecoinvent v 3.8 database sub process as detailed in Table S16[9]
		Aspiration system	8.36*10^{-6} p	Modelled from Ecoinvent v 3.8 database sub process as detailed in Table S8[10]
		Activated carbon air filter	3.33*10^{-5} p	Modelled from Ecoinvent v 3.8 database sub process as detailed in Table S9[11]
		MW Reactor Single Mode CEM Discover	3.04*10^{-6} p	Modelled from Ecoinvent v 3.8 database sub process as detailed in Table S19[12]
	Transport	Transport for raw materials	0.656 kgkm	Ecoinvent v 3.8[13]
		Transport for small equipment	1.74 kgkm	Ecoinvent v 3.8[14]
		Transport for large equipment	0.0118 tkm	Ecoinvent v 3.8[15]
	Energy	Electric Energy	0.082 kWh	Ecoinvent v 3.8[16]
		Electric Energy	12 W	Ecoinvent v 3.8[17]
		Electric Energy	500 W	Ecoinvent v 3.8[18]
		Electric Energy	0.75 kWh	Ecoinvent v 3.8[19]
		Electric Energy	0.475 kWh	Ecoinvent v 3.8[20]
Output	Emissions to Air	Hydrogen Chloride	2.58*10^{-7}g	SimaPro airborne emission substance list[21]
		Water	3.46*10^{-7}g	SimaPro airborne emission substance list[22]
	End of life	Spent solvent mixture	56.80 g	Ecoinvent v 3.8[23]
		Spent solvent mixture	0.01455 g	Ecoinvent v 3.8[24]

[1]Water contained in the exploded biomass. The total mass treated in a single experiment is 24.9 g and the fraction of water contained in it is 78.5%. The Ecoinvent process used was: Water, deionised {Europe without Switzerland} | water production, deionised | APOS, U.

[2]Hydrochloric Acid 37% used as catalyst during the hydrolysis experiment in the MW reactor. The hydrochloric acid mass used was calculated by multiplying the hydrochloric acid fraction (1.4 wt%) with the total mass of a single experiment (24.9 g). The Ecoinvent process used was: The Hydrochloric acid, without water, in 30% solution state {RER}| allyl chloride production, reaction of propylene and chlorine | APOS, U.
[3]Silicon Oil used to heat up the reaction mixture during the phase of purification of levulinic acid. The Ecoinvent process used was: Silicone product {RER}| production | APOS, U.
[4]The reported value was calculated by the following formula: 1/28800h*5h, where 28800 h is the lifetime of the round bottomed flask and 5h is the time of usage of the round bottomed flask.
[5]The reported value was calculated by the following formula: 1/100000h*4h, where 100000 h is the lifetime of the stir bar and 4h is the time of usage of the stir bar.
[6]The reported value was calculated by the following formula: 1/50000h*5h, where 50000 h is the lifetime of the magnetic stirrer/heater and 5h is the time of usage of the stirrer/heater.
[7]The reported value was calculated by the following formula: 1/28800h*1h, where 28800 h is the lifetime of the Vigreux column and 1h is the distillation time.
[8]The reported value was calculated by the following formula: 1/57600h*1h, where 57600 h is the lifetime of the silicone oil container and 1h is its time of usage.
[9]The reported value was calculated by the following formula: 1/19200h*1h, where 19200 h is the lifetime of the vacuum pump and 1h is its time of usage.
[10]The reported value was calculated by the following formula: 1/79750h*5.67h, where 79750 h is the lifetime of the modeled aspiration system, and 5.67 h is its time of usage, considering 40 minutes for microwave hydrolysis, 4 h for extraction and 1 h for distillation.
[11]The reported value was calculated by the following formula: 1/20000h*5.67h, where 20000 h is the lifetime of the modeled activated carbon air filter, and 5.67 h is its time of usage, considering 40 minutes for microwave hydrolysis, 4 h for extraction and 1 h for distillation.
[12]The reported value was calculated by the following formula: 1/219000h*40/60 h, where 219000 h is the lifetime of the microwave reactor and 40/60 h is the hydrolysis time.
[13]Transport for reagents. An average distance of 100 km was considered. The Ecoinvent process used was: Transport, freight, lorry 3.5-7.5 metric ton, EURO6 {RER}| transport, freight, lorry 3.5-7.5 metric ton, EURO6 | APOS, U.
[14]Transports for the small equipment. An average distance of 100 km was considered. The Ecoinvet process used was: Transport, freight, lorry 3.5-7.5 metric ton, EURO6 {RER}| transport, freight, lorry 3.5-7.5 metric ton, EURO6 | APOS, U.
[15]Transport for large equipment. An average distance of 100 km was considered. The Ecoinvent process used was: Transport, freight, lorry 16-32 metric ton, EURO6 {RER}| transport, freight, lorry 16-32 metric ton, EURO6 | APOS, U.
[16]Eletric energy necessary for the functioning of the MW reactor for the extraction of levulinic acid. The Ecoinvent process used was: Electricity, low voltage {IT}| electricity voltage transformation from medium to low voltage | APOS, U.
[17]Electric energy necessary to heat up the reaction mixture for four hours (containing levulinic acid and 2-methyltetrahydrofuran) during the purification of levulinic acid using a magnetic stirrer (power of 500W). The Ecoinvent process used was: Electricity, low voltage {IT}| electricity voltage transformation from medium to low voltage | APOS, U
[18]Electric energy necessary during the distillation (last step of the purification phase of levulinic acid) to heat up the reaction mixture using a magnetic stirrer (power of 500W). The Ecoinvent process used was: Electricity, low voltage {IT}| electricity voltage transformation from medium to low voltage | APOS, U.
[19]Electric energy required for the functioning of the Vacuum Pump (power of 0.75kW) during the distillation step. The Ecoinvent process used was: Electricity, low voltage {IT}| electricity voltage transformation from medium to low voltage | APOS, U.
[20]Electric energy necessary for the operation of the aspiration system for the whole synthesis time, i.e. 0.666667 h. The power was calculated by considering the air flow rate of 250 m^3/h, a total load loss of 110.8076 kg/m^2, and an efficiency of 90%. The Ecoinvent process used was: Electricity, low voltage {IT}| electricity voltage transformation from medium to low voltage | APOS, U.
[21]Amount of Hydrogen Chloride released into the atmosphere, as calculated by the formula reported in equation 1 of the main manuscript.
[22]Amount of Water released into the atmosphere, as calculated by the formula reported in equation 1 of the main manuscript.
[23]End of life treatment for all the waste resulting from the extraction and that are not levulinic acid. The Ecoinvent process used was: Spent solvent mixture {Europe without Switzerland}| treatment of spent solvent mixture, hazardous waste incineration, with energy recovey | APOS, U.
[24]End of life treatment for the silicon oil. The Ecoinvent process used was: Spent solvent mixture {Europe without Switzerland}| treatment of spent solvent mixture, hazardous waste incineration, with energy recovery | APOS, U.

Table S21. Contribution to the Life Cycle inventory (LCI) for the production of 1 p of a 100% borosilicate glass two neck round bottom flask, with a weight of 150 g. Its lifetime was assumed to be 15 years, during which it was assumed to be used for 8 h/day, for 5 days/week and for 48 weeks/year, thus for a total of 28800 h.

Description			Amount	Process data source
Input	Materials	Borosilicate glass	150 g	Ecoinvent v. 3.8[1]
	Transport	Transport of materials	15 kgkm	Ecoinvent v. 3.8[2]
Output	End of life	Landfilling	150 g	Ecoinvent v. 3.8[3]

[1]The Ecoinvent process used was: Glass tube, borosilicate {RoW}| production | APOS, U.

[2]Transport for the borosilicate glass. An average distance of 100 km was considered. The Ecoinvent process used was: Transport, freight, lorry 3.5-7.5 metric ton, EURO6 {RER}| transport, freight, lorry 3.5-7.5 metric ton, EURO6 | APOS, U.

[3]Borosilicate glass is not recycled, therefore for its end of life it is treated like and inert waste and delivered to landfill. The Ecoinvent process used was: Inert waste, for final disposal {RoW}| treatment of inert waste, inert material landfill | APOS, U.

Table S22. Contribution to the Life Cycle inventory (LCI) for the production of 1 p of a 100% borosilicate glass tailed flask, with a weight of 150 g. Its lifetime was assumed to be 15 years, during which it was assumed to be used for 8 h/day, for 5 days/week and for 48 weeks/year, thus for a total of 28800 h.

Description			Amount	Process data source
Input	Materials	Borosilicate glass	150 g	Ecoinvent v. 3.8[1]
	Transport	Transport of materials	15 kgkm	Ecoinvent v. 3.8[2]
Output	End of life	Landfilling	150 g	Ecoinvent v. 3.8[3]

[1]The Ecoinvent process used was: Glass tube, borosilicate {RoW}| production | APOS, U.

[2]Transport for the borosilicate glass. An average distance of 100 km was considered. The Ecoinvent process used was: Transport, freight, lorry 3.5-7.5 metric ton, EURO6 {RER}| transport, freight, lorry 3.5-7.5 metric ton, EURO6 | APOS, U.

[3]Borosilicate glass is not recycled, therefore for its end of life it is treated like and inert waste and delivered to landfill. The Ecoinvent process used was: Inert waste, for final disposal {RoW}| treatment of inert waste, inert material landfill | APOS, U.

Table S23. Contribution to the Life Cycle inventory (LCI) for the production of 1 p of Buchner filter, with a weight of 150 g. Its lifetime was assumed to be 15 years, during which it was assumed to be used for 8 h/day, for 5 days/week and for 48 weeks/year, thus for a total of 28800 h.

Description			Amount	Process data source
Input	Materials	Ceramic material	300 g	Ecoinvent v. 3.8[1]
		Paper	5 g	Ecoinvent v. 3.8[2]
	Transport	Transport of materials	30.5 kgkm	Ecoinvent v. 3.8[3]
Output	End of life	Landfilling	300 g	Ecoinvent v. 3.8[4]
		Incineration	5 g	Ecoinvent v. 3.8[5]

[1]The Ecoinvent process used was: Ceramic tile {RoW}| production | APOS, U.

[2]The Ecoinvent process used was: Paper, newsprint {RER}| paper production, newsprint, virgin | APOS, U.

[3]Transport of raw materials. An average distance of 100 km was considered. The Ecoinvent process used was: Transport, freight, lorry 3.5-7.5 metric ton, EURO6 {RER}| transport, freight, lorry 3.5-7.5 metric ton, EURO6 | APOS, U.

[4]End of life of the ceramic material. The Ecoinvet process used was: Inert waste, for final disposal {RoW}| treatment of inert waste, inert material landfill | APOS, U.
[5]End of life of the paper filter. The Ecoinvent process used was: Hazardous waste, for incineration {RoW}| treatment of hazardous waste, hazardous waste incineration | APOS, U.

Table S24. Contribution to the Life Cycle Inventory (LCI) for the production of 1 p of a rubber tube used during the vacuum filtration in the synthesis of para-toluenesulfonic acid. Its lifetime was assumed to be 15 years, during which it was assumed to be used for 8 h/day, for 5 days/week and for 48 weeks/year, thus for a total of 28800 h.

Description			Amount	Process Data Source
Input	Materials	Synthetic rubber	30 g	Ecoinvent v 3.8[1]
	Transport	Transport of raw materials	3 kgkm	Ecoinvent v 3.8[2]
Output	End of Life	Landfilling	30 g	Ecoinvent v 3.8[3]

[1]The Ecoinvent process used was: Synthetic rubber {RER}| production | APOS, U.
[2]The Ecoinvent process used was: Transport, freight, lorry 3.5-7.5 metric ton, EURO6 {RER}| transport, freight, lorry 3.5-7.5 metric ton, EURO6 | APOS, U. an average distance of 100 km was considered.
[3]End of life treatment for the rubber material. The Ecoinvent process used was: Inert waste, for final disposal {RoW}| treatment of inert waste, inert material landfill | APOS, U.

Table S25. Synthesis of 172.9 g of para-toluensulfonic acid used first during the synthesis of the hydroxyalkyl esters **2a-e** precursors and, again, during the synthesis of the plasticizers **4a-e**. This synthesis was modelled using the procedure reported in [10].

Description			Amount	Process Data Source
Input	Materials	Toluene	250 g	Ecoinvent v. 3.8[1]
		H_2SO_4	100 g	Ecoinvent v. 3.8[2]
		Ethanol	5 g	Ecoinvent v. 3.8[3]
		H_2O	15 g	Ecoinvent v. 3.8[4]
		Silicon oil	$7.28*10^{-4}$ kg	Ecoinvent v. 3.8[5]
	Equipment/plants	Two neck round bottom flask	$1.74*10^{-4}$ p	Modelled from Ecoinvent v 3.8 database sub process as detailed in Table S21[6]
		Buchner Filter	$8.68*10^{-6}$ p	Modelled from Ecoinvent v 3.8 database sub process as detailed in Table S23[7]
		Tailed flask	$8.68*10^{-6}$ p	Modelled from Ecoinvent v 3.8 database sub process as detailed in Table S22[8]
		Vacuum pump	$5*10^{-6}$ p	Modelled from Ecoinvent v 3.8 database sub process as detailed in Table S16[9]
		Magnetic stirrer	$1*10^{-4}$ p	Modelled from Ecoinvent v 3.8 database sub process as detailed in Table S15[10]

		Rubber tube	$8.68*10^{-6}$ p	Modelled from Ecoinvent v 3.8 database sub process as detailed in Table S24[11]
		Silicon oil container	$8.68*10^{-5}$ p	Modelled from Ecoinvent v 3.8 database sub process as detailed in Table S13[12]
		Aspiration system	$7.52*10^{-5}$ p	Modelled from Ecoinvent v 3.8 database sub process as detailed in Table S8[13]
		Activated carbon air filter	$3*10^{-4}$ p	Modelled from Ecoinvent v 3.8 database sub process as detailed in Table S9[14]
	Transports	Transport of reagents	37 kgkm	Ecoinvent v. 3.8[15]
		Transport of small materials	0.117 kgkm	Ecoinvent v. 3.8[16]
		Tramsport of large materials	0.0125 tkm	Ecoinvent v. 3.8[17]
	Energy	Electric energy	2500 Wh	Ecoinvent v. 3.8[18]
		Electric energy	187.5 Wh	Ecoinvent v. 3.8[19]
		Electric energy	2622,9 J	Ecoinvent v. 3.8[20]
		Electric energy	0.503 kWh	Ecoinvent v. 3.8[21]
Output	Emissions	Ethanol	$9.04*10^{-6}$ g	SimaPro airborne emission substance list[22]
		Toluene	$4.05*10^{-4}$ g	SimaPro airborne emission substance list[23]
		H_2SO_4	$2.884*10^{-9}$ g	SimaPro airborne emission substance list[24]
		H_2O	$3.45*10^{-6}$ g	SimaPro airborne emission substance list[25]
	End of life	Spent solvent mixture	197.02 g	Ecoinvent v 3.8[26]
		Spent Solvent mixture	$7.28*10^{-4}$ kg	Ecoinvent v 3.8[27]

[1]The Ecoinvet process used was: Toluene, liquid {RER}| production | APOS, U.

[2]The Ecoinvent process used was: Sulfuric acid {RER}| production | APOS, U.
[3]The Ecoinvent process used was: Ethanol, without water, in 99.7% solution state, from ethylene {RER}| ethylene hydration | APOS, U.
[4] The Ecoinvent process used was: Water, deionised {Europe without Switzerland}| water production, deionised | APOS, U.
[5]The Ecoinvent process used was: Silicone product {RER}| production | APOS, U.
[6]The reported value was calculated by the following formula: 1/28800h*5h, where 28800 h is the lifetime of the round bottomed flask and 5h is the time of usage of the round bottomed flask.
[7]The reported value was calculated by the following formula: 1/28800h*0.25h, where 28800 h is the lifetime of the Buchner filter and 0.25h is its time of usage.
[8]The reported value was calculated by the following formula: 1/28800h*0.25h, where 28800 h is the lifetime of the tailed flask and 0.25h is its time of usage.
[9]The reported value was calculated by the following formula: 1/50000h*0.25h, where 50000 h is the lifetime of the vacuum pump and 0.25h is its time of usage.
[10]The reported value was calculated by the following formula: 1/50000h*5h, where 50000 h is the lifetime of the magnetic stirrer and 5h is the time of usage of the magnetic stirrer.
[11]The reported value was calculated by the following formula: 1/28800h*0.25h, where 28800 h is the lifetime of the rubber tube and 0.25h is its time of usage.
[12]The reported value was calculated by the following formula: 1/57600h*5h, where 57600 h is the lifetime of the silicone oil container and 5h is its time of usage.
[13]The reported value was calculated by the following formula: 1/79750h*6h, where 79750 h is the lifetime of the aspiration system and 6h is its time of usage. One additional hour of functioning was considered for the work-up procedure.
[14]The reported value was calculated by the following formula: 1/20000h*6h, where 20000 h is the lifetime of the activated carbon air filter and 6h is its time of usage. One additional hour of functioning was considered for the work-up procedure.
[15]Transport of reagents. An average distance of 100 km was considered. The Ecoinvent process used was: Transport, freight, lorry 3.5-7.5 metric ton, EURO6 {RER}| transport, freight, lorry 3.5-7.5 metric ton, EURO6 | APOS, U.
[16]Transport of small materials/equipment. An average distance of 100 km was considered. The Ecoinvent process used was: Transport, freight, lorry 3.5-7.5 metric ton, EURO6 {RER}| transport, freight, lorry 3.5-7.5 metric ton, EURO6 | APOS, U.
[17]Transport of large equipment (aspiration system and activated carbon air filter). An average distance of 100 km was considered. The Ecoinvent process used was: Transport, freight, lorry 16-32 metric ton, EURO6 {RER}| transport, freight, lorry 16-32 metric ton, EURO6 | APOS, U.
[18]Electric energy necessary for the heating of the reaction mixture for 5 hours. It was calculated as power* time, considering the power of thee magnetic stirrer of 500W. The Ecoinvent process used was: Electricity, low voltage {IT}| electricity voltage transformation from medium to low voltage | APOS, U.
[19]Electric energy required for the functioning of the vacuum pump for 15 minutes. It is calculated as power*time, considering 750W as the vacuum pump power.
[20]Electric energy necessary for heating the reaction mixture (H_2O and Ethanol), starting from room temperature up to the boiling point of toluene. It was calculated as $m/Cp*\Delta T$, considering as specific heat 157 J/molK and 83.68 J/molK respectively for toluene and sulfuric acid. Electricity, low voltage {IT}| electricity voltage transformation from medium to low voltage | APOS, U.
[21]Electric energy necessary to the use of the aspiration system for the whole synthesis time, i.e. 6 h. The power was calculated by considering the air flow rate of 250 m^3/h, a total load loss of 110.8076 kg/m^2, and an efficiency of 90%. The Ecoinvent process used was: Electricity, low voltage {IT}| electricity voltage transformation from medium to low voltage | APOS, U.
[22]Amount of ethanol released into the atmosphere, as calculated by the formula reported in equation 1 of the main manuscript.
[23]Amount of toluene released into the atmosphere, as calculated by the formula reported in equation 1 of the main manuscript.
[24]Amount of H_2OS_4 released into the atmosphere, as calculated by the formula reported in equation 1 of the main manuscript.
[25]Amount of Water released into the atmosphere, as calculated by the formula reported in equation 1 of the main manuscript.
[26]End of life treatment for anything other than the final product. The Ecoinvent process used was: Spent solvent mixture {Europe without Switzerland}| treatment of spent solvent mixture, hazardous waste incineration, with energy recovery | APOS, U.
[27]End of life treatment foe the silicon oil. The Ecoinvent process used was: Spent solvent mixture {Europe without Switzerland}| treatment of spent solvent mixture, hazardous waste incineration, with energy recovery | APOS, U.

Table S26. Contributions for the Life Cycle Inventory (LCI) for the production of 1.29 g of hydroxyalkyl ester **2a** according to the procedure detailed in [11].

Description			Amount	Process Data Source
Input	Materials			
		Myristic Acid	1.14 g	Ecoinvent v 3.8[1]
		Ethylene Glycol	6.21 g	Ecoinvent v 3.8[2]
		p-toluenesulfonic acid	0.0476 g	Modelled from Ecoinvent v 3.8 database sub process as detailed in Table S25
		K_2CO_3	10 g	Ecoinvent v 3.8[3]
		H_2O	90 g	Ecoinvent v 3.8[4]
		Diethyl ether	35.5 g	Ecoinvent v 3.8[5]
		H_2O	150 g	Ecoinvent v 3.8[6]
		NaCl	50 g	Ecoinvent v 3.8[7]
		Na_2SO_4	10 g	Ecoinvent v 3.8[8]
		Silicon Oil	0.026 kg	Ecoinvent v 3.8[9]
	Equipment/plants	Round bottom flask	$6.25*10^{-4}$ p	Modelled from Ecoinvent v 3.8 database sub process as detailed in Table S11[10]
		Silicon Oil container	$3.13*10^{-3}$ p	Modelled from Ecoinvent v 3.8 database sub process as detailed in Table S13[11]
		Magnetic stirrer	$3.6*10^{-4}$ p	Modelled from Ecoinvent v 3.8 database sub process as detailed in Table S15[12]
		Magnetic stirr bar	$1.8*10^{-4}$ p	Modelled from Ecoinvent v 3.8 database sub process as detailed in Table S14[13]
		Aspiration system	$2.38*10^{-4}$ p	Modelled from Ecoinvent v 3.8 database sub process as detailed in Table S8[14]
		Activated carbon air filter	$9.5*10^{-4}$ p	Modelled from Ecoinvent v 3.8 database sub process as detailed in Table S9[15]
	Transports	Transport of raw materials	35.29 kgkm	Ecoinvent v3.8[16]
		Transport of small equipment	0.395 kgkm	Ecoinvent v 3.8[17]
		Transport of large equipment/plants	$3.96*10^{-2}$ tkm	Ecoinvent v 3.8[18]
	Energy	Electric Energy	900 W	Ecoinvent v 3.8[19]
		Electric Energy	12.93 kJ	Ecoinvent v 3.8[20]
		Electric Energy	1.59 kWh	Ecoinvent v 3.8[21]

Output	Avoided Products	Diethyl ether	33.64 g	Ecoinvent v 3.8[22]
	Emission to air	Ethylene glycol	$1.72*10^{-8}$ g	SimaPro airborne emission substance list[23]
		Diethyl ether	$8.78*10^{-4}$ g	SimaPro airborne emission substance list[24]
		H_2O	$6.22*10^{-5}$ g	SimaPro airborne emission substance list[25]
	End of Life	Spent Solvent Mixture	317.78 g	Ecoinvent v 3.8[26]
		Spent Solvent Mixture	$2.62*10^{-3}$ g	Ecoinvent v 3.8[27]

[1]Carboxylic acid used to start the synthesis of the precursor 2A. The Myristic acid used here was modified by some of the authors. The Ecoinvent process "Fatty acid (RoW) fatty acid production. from coconut oil APOS, U" was used as a reference. This process allows to obtain all the fatty acids obtainable from coconut oil and it also takes into consideration the splitting step to produce single and specific acids [12]. A 21% of Myristic Acid is produced by coconut oil, according to Anneken et al [13], therefore all the inputs and outputs of the process mentioned above have been scaled to a 21% to model myristic acid production.

[2]The Ecoinvent process used was: Ethylene glycol {RER}| production | APOS, U.

[3]Sodium carbonate was used during the work up procedure. This material wasn't available on the Ecoinvent database, so Potassium Carbonate was chosen instead. The Ecoinvent process used was: Potassium carbonate {GLO}| production, from potassium hydroxide | APOS, U.

[4]Water necessary for the preparation of the solution of diethyl ether. The Ecoinvent process used was: Water, deionised {Europe without Switzerland} | water production, deionised | APOS, U.

[5]Diethyl ether used during the workup procedure for the extraction of the organic phase. The Ecoinvent process used was: Diethyl ether, without water, in 99.95% solution state {RoW}| ethylene hydration | APOS, U.

[6]Water used for the washing of the organic phase, after the extraction with diethyl ether, in order to remove the residual catalyst. The Ecoinvent process used was: Water, deionised {Europe without Switzerland} | water production, deionised | APOS, U.

[7]Sodium chloride used to wash the organic phase to remove unreacted reagents. The Ecoinvent process used was: Sodium chloride, brine solution {RER}| production | APOS, U.

[8]Sodium sulphate anhydrous used to remove the residual water in the organic phase. The Ecoinvent process used was: Sodium sulphate, anhydrite {RER}| Mannheim process | APOS, U.

[9]The Ecoinvent process used was: Silicone product {RER}| production | APOS, U.

[10]The reported value was calculated by the following formula: 1/28800h*18h, where 28800 h is the lifetime of the round bottomed flask and 18h is the time of usage of the round bottomed flask.

[11]The reported value was calculated by the following formula: 1/57600h*18h, where 57600 h is the lifetime of the silicone oil container and 18h is its time of usage.

[12]The reported value was calculated by the following formula: 1/50000h*18h, where 50000 h is the lifetime of the magnetic stirrer and 18h is the time of usage of the magnetic stirrer.

[13]The reported value was calculated by the following formula: 1/100000h*18h, where 100000 h is the lifetime of the stir bar and 18h is its time of usage.

[14]The reported value was calculated by the following formula: 1/79750h*19h, where 79750 h is the lifetime of the aspiration system and 19h is its time of usage. One additional hour of functioning was considered for the work-up procedure.

[15]The reported value was calculated by the following formula: 1/20000h*19h, where 20000 h is the lifetime of the activated carbon air filter and 19h is its time of usage. One additional hour of functioning was considered for the work-up procedure.

[16]Transport of raw materials (reagents). The Ecoinvent process used was: Transport, freight, lorry 3.5-7.5 metric ton, EURO6 {RER}| transport, freight, lorry 3.5-7.5 metric ton, EURO6 | APOS, U. An average distance of 100 km was considered.

[17]Transport of small equipment. The Ecoinvent process used was: Transport, freight, lorry 3.5-7.5 metric ton, EURO6 {RER}| transport, freight, lorry 3.5-7.5 metric ton, EURO6 | APOS, U. an average distance of 100 km was considered.

[18]Transport of large equipment (aspiration system and activated carbon air filter). The Ecoinvent process used was: Transport, freight, lorry 16-32 metric ton, euro6 {RER}| market for transport, freight, lorry 16-32 metric ton, EURO6 | APOS, U. an average distance of 100 km was considered.
[19]Electric energy necessary to heat up the reaction mixture at 75°C. It was calculated as power*time, considering the reaction time to be 18 hours and the power of heating/magnetic stirrer to be 500W. The Ecoinvent process used was: Electricity, low voltage {IT}| electricity voltage transformation from medium to low voltage | APOS, U.
[20]Electric energy necessary to evaporate diethyl ether, calculated using the vaporization enthalpy of the substance, 27 kJ/mol. The Ecoinvent process used was: Electricity, low voltage {IT}| electricity voltage transformation from medium to low voltage | APOS, U..
[21]Electric energy necessary to the use of the aspiration system for the whole synthesis time, i.e. 19 h. The power was calculated by considering the air flow rate of 250 m^3/h, a total load loss of 110.8076 kg/m^2, and an efficiency of 90%. The Ecoinvent process used was: Electricity, low voltage {IT}| electricity voltage transformation from medium to low voltage | APOS, U.
[22]Avoided Products, it is assumed that 95% of Diethyl ether can be recovered. The Ecoinvent process used was: Diethyl ether, without water, in 99.95% solution state {RoW}| ethylene hydration | APOS, U.
[23]Amount of ethylene glycol released into the atmosphere, as calculated by the formula reported in equation 1 of the main manuscript.
[24]Amount of Diethyl ether released into the atmosphere, as calculated by the formula reported in equation 1 of the main manuscript.
[25]Amount of Water released into the atmosphere, as calculated by the formula reported in equation 1 of the main manuscript.
[26]End of life treatment for what was used during the synthesis, work up procedure and isolation of the final product. The Ecoinvent process used was: Spent solvent mixture {Europe without Switzerland}| treatment of spent solvent mixture, hazardous waste incineration, with energy recovery | APOS, U.
[27]End of life for the silicon oil. Ecoinvent process used was: Spent solvent mixture {Europe without Switzerland}| treatment of spent solvent mixture, hazardous waste incineration, with energy recovery | APOS, U.

Table S27. Contributions for the Life Cycle Inventory (LCI) for the production of 1.31 g of hydroxyalkyl ester **2b** according to the procedure detailed in [11].

Description			Amount	Process Data Source
Input	Materials			
		Stearic Acid	1.42 g	Ecoinvent v 3.8[1]
		Ethylene Glycol	6.21 g	Ecoinvent v 3.8[2]
		p-toluenesulfonic acid	0.0476 g	Modelled from Ecoinvent v 3.8 database sub process as detailed in Table S25
		K_2CO_3	10 g	Ecoinvent v 3.8[3]
		H_2O	90 g	Ecoinvent v 3.8[4]
		Diethyl ether	35.5 g	Ecoinvent v 3.8[5]
		H_2O	150 g	Ecoinvent v 3.8[6]
		NaCl	50 g	Ecoinvent v 3.8[7]
		Na_2SO_4	10 g	Ecoinvent v 3.8[8]
		Silicon Oil	0.026 kg	Ecoinvent v 3.8[9]
	Equipment/plants	Round bottom flask	$6.25*10^{-4}$ p	Modelled from Ecoinvent v 3.8 database sub process as detailed in Table S11[10]
		Silicon Oil container	$3.13*10^{-3}$ p	Modelled from Ecoinvent v 3.8 database sub process as detailed in Table S13[11]

		Magnetic stirrer	$3.6*10^{-4}$ p	Modelled from Ecoinvent v 3.8 database sub process as detailed in Table S15[12]
		Magnetic stirr bar	$1.8*10^{-4}$ p	Modelled from Ecoinvent v 3.8 database sub process as detailed in Table S14[13]
		Aspiration system	$2.38*10^{-4}$ p	Modelled from Ecoinvent v 3.8 database sub process as detailed in Table S8[14]
		Activated carbon air filter	$9.5*10^{-4}$ p	Modelled from Ecoinvent v 3.8 database sub process as detailed in Table S9[15]
	Transports	Transport of raw materials	35.29 kgkm	Ecoinvent v3.8[16]
		Transport of small equipment	0.395 kgkm	Ecoinvent v 3.8[17]
		Transport of large equipment/plants	$3.96*10^{-2}$ tkm	Ecoinvent v 3.8[18]
	Energy	Electric Energy	900 W	Ecoinvent v 3.8[19]
		Electric Energy	12.93 kJ	Ecoinvent v 3.8[20]
		Electric Energy	1.59 kWh	Ecoinvent v 3.8[21]
Output	Avoided Products	Diethyl ether	33.64 g	Ecoinvent v 3.8[22]
	Emission to air	Ethylene glycol	$1.72*10^{-8}$ g	SimaPro airborne emission substance list[23]
		Diethyl ether	$8.78*10^{-4}$ g	SimaPro airborne emission substance list[24]
		H_2O	$6.22*10^{-5}$ g	SimaPro airborne emission substance list[25]
	End of Life	Spent Solvent Mixture	318.04 g	Ecoinvent v 3.8[26]
		Spent Solvent Mixture	$2.62*10^{-3}$ g	Ecoinvent v 3.8[27]

[1]Carboxylic acid used to start the synthesis of the precursor 2B. The Ecoinvent process used was: Stearic acid {GLO}| stearic acid production | APOS, U.
[2]The Ecoinvent process used was: Ethylene glycol {RER}| production | APOS, U.
[3]Sodium carbonate was used during the work up procedure. This material wasn't available on the Ecoinvent database, so Potassium Carbonate was chosen instead. The Ecoinvent process used was: Potassium carbonate {GLO}| production, from potassium hydroxide | APOS, U.
[4]Water necessary for the preparation of the solution of diethyl ether. The Ecoinvent process used was: Water, deionised {Europe without Switzerland} | water production, deionised | APOS, U.
[5]Diethyl ether used during the workup procedure for the extraction of the organic phase. The Ecoinvent process used was: Diethyl ether, without water, in 99.95% solution state {RoW}| ethylene hydration | APOS, U.
[6]Water used for the washing of the organic phase, after the extraction with diethyl ether, in order to remove the residual catalyst. The Ecoinvent process used was: Water, deionised {Europe without Switzerland} | water production, deionised | APOS, U.
[7]Sodium chloride used to wash the organic phase to remove unreacted reagents. The Ecoinvent process used was: Sodium chloride, brine solution {RER}| production | APOS, U.

[8]Sodium sulphate anhydrous used to remove the residual water in the organic phase. The Ecoinvent process used was: Sodium sulphate, anhydrite {RER}| Mannheim process | APOS, U.
[9]The Ecoinvent process used was: Silicone product {RER}| production | APOS, U.
[10]The reported value was calculated by the following formula: 1/28800h*18h, where 28800 h is the lifetime of the round bottomed flask and 18h is the time of usage of the round bottomed flask.
[11]The reported value was calculated by the following formula: 1/57600h*18h, where 57600 h is the lifetime of the silicone oil container and 18h is its time of usage.
[12]The reported value was calculated by the following formula: 1/50000h*18h, where 50000 h is the lifetime of the magnetic stirrer and 18h is the time of usage of the magnetic stirrer.
[13]The reported value was calculated by the following formula: 1/100000h*18h, where 100000 h is the lifetime of the stir bar and 18h is its time of usage.
[14]The reported value was calculated by the following formula: 1/79750h*19h, where 79750 h is the lifetime of the aspiration system and 19h is its time of usage. One additional hour of functioning was considered for the work-up procedure.
[15]The reported value was calculated by the following formula: 1/20000h*19h, where 20000 h is the lifetime of the activated carbon air filter and 19h is its time of usage. One additional hour of functioning was considered for the work-up procedure.
[16]Transport of raw materials (reagents). The Ecoinvent process used was: Transport, freight, lorry 3.5-7.5 metric ton, EURO6 {RER}| transport, freight, lorry 3.5-7.5 metric ton, EURO6 | APOS, U. An average distance of 100 km was considered.
[17]Transport of small equipment. The Ecoinvent process used was: Transport, freight, lorry 3.5-7.5 metric ton, EURO6 {RER}| transport, freight, lorry 3.5-7.5 metric ton, EURO6 | APOS, U. an average distance of 100 km was considered.
[18]Transport of large equipment (aspiration system and activated carbon air filter). The Ecoinvent process used was: Transport, freight, lorry 16-32 metric ton, euro6 {RER}| market for transport, freight, lorry 16-32 metric ton, EURO6 | APOS, U. an average distance of 100 km was considered.
[19]Electric energy necessary to heat up the reaction mixture at 75°C. It was calculated as power*time, considering the reaction time to be 18 hours and the power of heating/magnetic stirrer to be 500W. The Ecoinvent process used was: Electricity, low voltage {IT}| electricity voltage transformation from medium to low voltage | APOS, U.
[20]Electric energy necessary to evaporate diethyl ether, calculated using the vaporization enthalpy of the substance, 27 kJ/mol. The Ecoinvent process used was: Electricity, low voltage {IT}| electricity voltage transformation from medium to low voltage | APOS, U..
[21]Electric energy necessary to the use of the aspiration system for the whole synthesis time, i.e. 19 h. The power was calculated by considering the air flow rate of 250 m^3/h, a total load loss of 110.8076 kg/m^2, and an efficiency of 90%. The Ecoinvent process used was: Electricity, low voltage {IT}| electricity voltage transformation from medium to low voltage | APOS, U.
[22]Avoided Products, it is assumed that 95% of Diethyl ether can be recovered. The Ecoinvent process used was: Diethyl ether, without water, in 99.95% solution state {RoW}| ethylene hydration | APOS, U.
[23]Amount of ethylene glycol released into the atmosphere, as calculated by the formula reported in equation 1 of the main manuscript.
[24]Amount of Diethyl ether released into the atmosphere, as calculated by the formula reported in equation 1 of the main manuscript.
[25]Amount of Water released into the atmosphere, as calculated by the formula reported in equation 1 of the main manuscript.
[26]End of life treatment for what was used during the synthesis, work up procedure and isolation of the final product. The Ecoinvent process used was: Spent solvent mixture {Europe without Switzerland}| treatment of spent solvent mixture, hazardous waste incineration, with energy recovery | APOS, U.
[27]End of life for the silicon oil. Ecoinvent process used was: Spent solvent mixture {Europe without Switzerland}| treatment of spent solvent mixture, hazardous waste incineration, with energy recovery | APOS, U.

Table S28. Contributions for the Life Cycle Inventory (LCI) for the production of 0.62 g of hydroxyalkyl ester **2c** according to the procedure detailed in [11].

		Description	Amount	Process Data Source
Input	Materials			
		Propionic Acid	0.51 g	Ecoinvent v 3.8[1]
		Ethylene Glycol	6.21 g	Ecoinvent v 3.8[2]

		p-toluenesulfonic acid	0.0476 g	Modelled from Ecoinvent v 3.8 database sub process as detailed in Table S25
		K_2CO_3	10 g	Ecoinvent v 3.8[3]
		H_2O	90 g	Ecoinvent v 3.8[4]
		Diethyl ether	35.5 g	Ecoinvent v 3.8[5]
		H_2O	150 g	Ecoinvent v 3.8[6]
		NaCl	50 g	Ecoinvent v 3.8[7]
		Na_2SO_4	10 g	Ecoinvent v 3.8[8]
		Silicon Oil	0.026 kg	Ecoinvent v 3.8[9]
	Equipment/plants	Round bottom flask	$6.25*10^{-4}$ p	Modelled from Ecoinvent v 3.8 database sub process as detailed in Table S11[10]
		Silicon Oil container	$3.13*10^{-3}$ p	Modelled from Ecoinvent v 3.8 database sub process as detailed in Table S13[11]
		Magnetic stirrer	$3.6*10^{-4}$ p	Modelled from Ecoinvent v 3.8 database sub process as detailed in Table S15[12]
		Magnetic stirr bar	$1.8*10^{-4}$ p	Modelled from Ecoinvent v 3.8 database sub process as detailed in Table S14[13]
		Aspiration system	$2.38*10^{-4}$ p	Modelled from Ecoinvent v 3.8 database sub process as detailed in Table S8[14]
		Activated carbon air filter	$9.5*10^{-4}$ p	Modelled from Ecoinvent v 3.8 database sub process as detailed in Table S9[15]
	Transports	Transport of raw materials	35.29 kgkm	Ecoinvent v3.8[16]
		Transport of small equipment	0.395 kgkm	Ecoinvent v 3.8[17]
		Transport of large equipment/plants	$3.96*10^{-2}$ tkm	Ecoinvent v 3.8[18]
	Energy	Electric Energy	900 W	Ecoinvent v 3.8[19]
		Electric Energy	12.93 kJ	Ecoinvent v 3.8[20]
		Electric Energy	1.59 kWh	Ecoinvent v 3.8[21]
Output	Avoided Products	Diethyl ether	33.64 g	Ecoinvent v 3.8[22]
	Emission to air	Ethylene glycol	$1.72*10^{-8}$ g	SimaPro airborne emission substance list[23]
		Diethyl ether	$8.78*10^{-4}$ g	SimaPro airborne emission substance list[24]

	H_2O	$6.22*10^{-5}$ g	SimaPro airborne emission substance list[25]
End of Life	Spent Solvent Mixture	317.83 g	Ecoinvent v 3.8[26]
	Spent Solvent Mixture	$2.62*10^{-3}$ g	Ecoinvent v 3.8[27]

[1]Carboxylic acid used to start the synthesis of the precursor 2C. The Ecoinvent process used was: Propionic acid {RER}| production | APOS, U. Experimentally Isovaleric Acid was used but is was not present in the database, so Propionic Acid was used instead.
[2]The Ecoinvent process used was: Ethylene glycol {RER}| production | APOS, U.
[3]Sodium carbonate was used during the work up procedure. This material wasn't available on the Ecoinvent database, so Potassium Carbonate was chosen instead. The Ecoinvent process used was: Potassium carbonate {GLO}| production, from potassium hydroxide | APOS, U.
[4]Water necessary for the preparation of the solution of diethyl ether. The Ecoinvent process used was: Water, deionised {Europe without Switzerland} | water production, deionised | APOS, U.
[5]Diethyl ether used during the workup procedure for the extraction of the organic phase. The Ecoinvent process used was: Diethyl ether, without water, in 99.95% solution state {RoW}| ethylene hydration | APOS, U.
[6]Water used for the washing of the organic phase, after the extraction with diethyl ether, in order to remove the residual catalyst. The Ecoinvent process used was: Water, deionised {Europe without Switzerland} | water production, deionised | APOS, U.
[7]Sodium chloride used to wash the organic phase to remove unreacted reagents. The Ecoinvent process used was: Sodium chloride, brine solution {RER}| production | APOS, U.
[8]Sodium sulphate anhydrous used to remove the residual water in the organic phase. The Ecoinvent process used was: Sodium sulphate, anhydrite {RER}| Mannheim process | APOS, U.
[9]The Ecoinvet process used was: Silicone product {RER}| production | APOS, U.
[10]The reported value was calculated by the following formula: 1/28800h*18h, where 28800 h is the lifetime of the round bottomed flask and 18h is the time of usage of the round bottomed flask.
[11]The reported value was calculated by the following formula: 1/57600h*18h, where 57600 h is the lifetime of the silicone oil container and 18h is its time of usage.
[12]The reported value was calculated by the following formula: 1/50000h*18h, where 50000 h is the lifetime of the magnetic stirrer and 18h is the time of usage of the magnetic stirrer.
[13]The reported value was calculated by the following formula: 1/100000h*18h, where 100000 h is the lifetime of the stir bar and 18h is its time of usage.
[14]The reported value was calculated by the following formula: 1/79750h*19h, where 79750 h is the lifetime of the aspiration system and 19h is its time of usage. One additional hour of functioning was considered for the work-up procedure.
[15]The reported value was calculated by the following formula: 1/20000h*19h, where 20000 h is the lifetime of the activated carbon air filter and 19h is its time of usage. One additional hour of functioning was considered for the work-up procedure.
[16]Transport of raw materials (reagents). The Ecoinvent process used was: Transport, freight, lorry 3.5-7.5 metric ton, EURO6 {RER}| transport, freight, lorry 3.5-7.5 metric ton, EURO6 | APOS, U. An average distance of 100 km was considered.
[17]Transport of small equipment. The Ecoinvent process used was: Transport, freight, lorry 3.5-7.5 metric ton, EURO6 {RER}| transport, freight, lorry 3.5-7.5 metric ton, EURO6 | APOS, U. an average distance of 100 km was considered.
[18]Transport of large equipment (aspiration system and activated carbon air filter). The Ecoinvent process used was: Transport, freight, lorry 16-32 metric ton, euro6 {RER}| market for transport, freight, lorry 16-32 metric ton, EURO6 | APOS, U. an average distance of 100 km was considered.
[19]Electric energy necessary to heat up the reaction mixture at 75°C. It was calculated as power*time, considering the reaction time to be 18 hours and the power of heating/magnetic stirrer to be 500W. The Ecoinvent process used was: Electricity, low voltage {IT}| electricity voltage transformation from medium to low voltage | APOS, U.
[20]Electric energy necessary to evaporate diethyl ether, calculated using the vaporization enthalpy of the substance, 27 kJ/mol. The Ecoinvent process used was: Electricity, low voltage {IT}| electricity voltage transformation from medium to low voltage | APOS, U.
[21]Electric energy necessary to the use of the aspiration system for the whole synthesis time, i.e. 19 h. The power was calculated by considering the air flow rate of 250 m^3/h, a total load loss of 110.8076 kg/m^2, and an efficiency of 90%. The Ecoinvent process used was: Electricity, low voltage {IT}| electricity voltage transformation from medium to low voltage | APOS, U.
[22]Avoided Products, it is assumed that 95% of Diethyl ether can be recovered. The Ecoinvent process used was: Diethyl ether, without water, in 99.95% solution state {RoW}| ethylene hydration | APOS, U.

[23]Amount of ethylene glycol released into the atmosphere, as calculated by the formula reported in equation 1 of the main manuscript.
[24]Amount of Diethyl ether released into the atmosphere, as calculated by the formula reported in equation 1 of the main manuscript.
[25]Amount of Water released into the atmosphere, as calculated by the formula reported in equation 1 of the main manuscript.
[26]End of life treatment for what was used during the synthesis, work up procedure and isolation of the final product. The Ecoinvent process used was: Spent solvent mixture {Europe without Switzerland}| treatment of spent solvent mixture, hazardous waste incineration, with energy recovery | APOS, U.
[27]End of life for the silicon oil. Ecoinvent process used was: Spent solvent mixture {Europe without Switzerland}| treatment of spent solvent mixture, hazardous waste incineration, with energy recovery | APOS, U.

Table S29. Contributions for the Life Cycle Inventory (LCI) for the production of 0.69 g of hydroxyalkyl ester **2d** according to the procedure detailed in [11].

Description			Amount	Process Data Source
Input	Materials			
		Benzoic Acid	0.56 g	Ecoinvent v 3.8[1]
		Ethylene Glycol	6.21 g	Ecoinvent v 3.8[2]
		p-toluenesulfonic acid	0.0476 g	Modelled from Ecoinvent v 3.8 database sub process as detailed in Table S25
		K_2CO_3	10 g	Ecoinvent v 3.8[3]
		H_2O	90 g	Ecoinvent v 3.8[4]
		Diethyl ether	35.5 g	Ecoinvent v 3.8[5]
		H_2O	150 g	Ecoinvent v 3.8[6]
		NaCl	50 g	Ecoinvent v 3.8[7]
		Na_2SO_4	10 g	Ecoinvent v 3.8[8]
		Silicon Oil	0.026 kg	Ecoinvent v 3.8[9]
	Equipment/plants	Round bottom flask	$6.25*10^{-4}$ p	Modelled from Ecoinvent v 3.8 database sub process as detailed in Table S11[10]
		Silicon Oil container	$3.13*10^{-3}$ p	Modelled from Ecoinvent v 3.8 database sub process as detailed in Table S13[11]
		Magnetic stirrer	$3.6*10^{-4}$ p	Modelled from Ecoinvent v 3.8 database sub process as detailed in Table S15[12]
		Magnetic stirr bar	$1.8*10^{-4}$ p	Modelled from Ecoinvent v 3.8 database sub process as detailed in Table S14[13]
		Aspiration system	$2.38*10^{-4}$ p	Modelled from Ecoinvent v 3.8 database sub process as detailed in Table S8[14]
		Activated carbon air filter	$9.5*10^{-4}$ p	Modelled from Ecoinvent v 3.8 database sub process as detailed in Table S9[15]

	Transports	Transport of raw materials	35.29 kgkm	Ecoinvent v3.8[16]
		Transport of small equipment	0.395 kgkm	Ecoinvent v 3.8[17]
		Transport of large equipment/plants	3.96*10^{-2} tkm	Ecoinvent v 3.8[18]
	Energy	Electric Energy	900 W	Ecoinvent v 3.8[19]
		Electric Energy	12.93 kJ	Ecoinvent v 3.8[20]
		Electric Energy	1.59 kWh	Ecoinvent v 3.8[21]
Output	Avoided Products	Diethyl ether	33.64 g	Ecoinvent v 3.8[22]
	Emission to air	Ethylene glycol	1.72*10^{-8} g	SimaPro airborne emission substance list[23]
		Diethyl ether	8.78*10^{-4} g	SimaPro airborne emission substance list[24]
		H_2O	6.22*10^{-5} g	SimaPro airborne emission substance list[25]
	End of Life	Spent Solvent Mixture	317.80 g	Ecoinvent v 3.8[26]
		Spent Solvent Mixture	2.62*10^{-3} g	Ecoinvent v 3.8[27]

[1]Carboxylic acid used to start the synthesis of the precursor 2D. The Ecoinvent process used was: Benzoic acid {RER}| toluene oxidation | APOS, U.

[2]The Ecoinvent process used was: Ethylene glycol {RER}| production | APOS, U.

[3]Sodium carbonate was used during the work up procedure. This material wasn't available on the Ecoinvent database, so Potassium Carbonate was chosen instead. The Ecoinvent process used was: Potassium carbonate {GLO}| production, from potassium hydroxide | APOS, U.

[4]Water necessary for the preparation of the solution of diethyl ether. The Ecoinvent process used was: Water, deionised {Europe without Switzerland} | water production, deionised | APOS, U.

[5]Diethyl ether used during the workup procedure for the extraction of the organic phase. The Ecoinvent process used was: Diethyl ether, without water, in 99.95% solution state {RoW}| ethylene hydration | APOS, U.

[6]Water used for the washing of the organic phase, after the extraction with diethyl ether, in order to remove the residual catalyst. The Ecoinvent process used was: Water, deionised {Europe without Switzerland} | water production, deionised | APOS, U.

[7]Sodium chloride used to wash the organic phase to remove unreacted reagents. The Ecoinvent process used was: Sodium chloride, brine solution {RER}| production | APOS, U.

[8]Sodium sulphate anhydrous used to remove the residual water in the organic phase. The Ecoinvent process used was: Sodium sulphate, anhydrite {RER}| Mannheim process | APOS, U.

[9]The Ecoinvent process used was: Silicone product {RER}| production | APOS, U.

[10]The reported value was calculated by the following formula: 1/28800h*18h, where 28800 h is the lifetime of the round bottomed flask and 18h is the time of usage of the round bottomed flask.

[11]The reported value was calculated by the following formula: 1/57600h*18h, where 57600 h is the lifetime of the silicone oil container and 18h is its time of usage.

[12]The reported value was calculated by the following formula: 1/50000h*18h, where 50000 h is the lifetime of the magnetic stirrer and 18h is the time of usage of the magnetic stirrer.

[13]The reported value was calculated by the following formula: 1/100000h*18h, where 100000 h is the lifetime of the stir bar and 18h is its time of usage.

[14]The reported value was calculated by the following formula: 1/79750h*19h, where 79750 h is the lifetime of the aspiration system and 19h is its time of usage. One additional hour of functioning was considered for the work-up procedure.

[15]The reported value was calculated by the following formula: 1/20000h*19h, where 20000 h is the lifetime of the activated carbon air filter and 19h is its time of usage. One additional hour of functioning was considered for the work-up procedure.
[16]Transport of raw materials (reagents). The Ecoinvent process used was: Transport, freight, lorry 3.5-7.5 metric ton, EURO6 {RER}| transport, freight, lorry 3.5-7.5 metric ton, EURO6 | APOS, U. An average distance of 100 km was considered.
[17]Transport of small equipment. The Ecoinvent process used was: Transport, freight, lorry 3.5-7.5 metric ton, EURO6 {RER}| transport, freight, lorry 3.5-7.5 metric ton, EURO6 | APOS, U. an average distance of 100 km was considered.
[18]Transport of large equipment (aspiration system and activated carbon air filter). The Ecoinvent process used was: Transport, freight, lorry 16-32 metric ton, euro6 {RER}| market for transport, freight, lorry 16-32 metric ton, EURO6 | APOS, U. an average distance of 100 km was considered.
[19]Electric energy necessary to heat up the reaction mixture at 75°C. It was calculated as power*time, considering the reaction time to be 18 hours and the power of heating/magnetic stirrer to be 500W. The Ecoinvent process used was: Electricity, low voltage {IT}| electricity voltage transformation from medium to low voltage | APOS, U.
[20]Electric energy necessary to evaporate diethyl ether, calculated using the vaporization enthalpy of the substance, 27 kJ/mol. The Ecoinvent process used was: Electricity, low voltage {IT}| electricity voltage transformation from medium to low voltage | APOS, U..
[21]Electric energy necessary to the use of the aspiration system for the whole synthesis time, i.e. 19 h. The power was calculated by considering the air flow rate of 250 m^3/h, a total load loss of 110.8076 kg/m^2, and an efficiency of 90%. The Ecoinvent process used was: Electricity, low voltage {IT}| electricity voltage transformation from medium to low voltage | APOS, U.
[22]Avoided Products, it is assumed that 95% of Diethyl ether can be recovered. The Ecoinvent process used was: Diethyl ether, without water, in 99.95% solution state {RoW}| ethylene hydration | APOS, U.
[23]Amount of ethylene glycol released into the atmosphere, as calculated by the formula reported in equation 1 of the main manuscript.
[24]Amount of Diethyl ether released into the atmosphere, as calculated by the formula reported in equation 1 of the main manuscript.
[25]Amount of Water released into the atmosphere, as calculated by the formula reported in equation 1 of the main manuscript.
[26]End of life treatment for what was used during the synthesis, work up procedure and isolation of the final product. The Ecoinvent process used was: Spent solvent mixture {Europe without Switzerland}| treatment of spent solvent mixture, hazardous waste incineration, with energy recovery | APOS, U.
[27]End of life for the silicon oil. Ecoinvent process used was: Spent solvent mixture {Europe without Switzerland}| treatment of spent solvent mixture, hazardous waste incineration, with energy recovery | APOS, U.

Table S30. Contributions for the Life Cycle Inventory (LCI) for the production of 0.77 g of hydroxyalkyl ester **2e** according to the procedure detailed in [11].

		Description	Amount	Process Data Source
Input	Materials			
		Phenil Acetic Acid	0.68 g	Ecoinvent v 3.8[1]
		Ethylene Glycol	6.21 g	Ecoinvent v 3.8[2]
		p-toluenesulfonic acid	0.0476 g	Modelled from Ecoinvent v 3.8 database sub process as detailed in Table S25
		K_2CO_3	10 g	Ecoinvent v 3.8[3]
		H_2O	90 g	Ecoinvent v 3.8[4]
		Diethyl ether	35.5 g	Ecoinvent v 3.8[5]
		H_2O	150 g	Ecoinvent v 3.8[6]
		NaCl	50 g	Ecoinvent v 3.8[7]
		Na_2SO_4	10 g	Ecoinvent v 3.8[8]

		Silicon Oil	0.026 kg	Ecoinvent v 3.8[9]
	Equipment/plants	Round bottom flask	$6.25*10^{-4}$ p	Modelled from Ecoinvent v 3.8 database sub process as detailed in Table S11[10]
		Silicon Oil container	$3.13*10^{-3}$ p	Modelled from Ecoinvent v 3.8 database sub process as detailed in Table S13[11]
		Magnetic stirrer	$3.6*10^{-4}$ p	Modelled from Ecoinvent v 3.8 database sub process as detailed in Table S15[12]
		Magnetic stirr bar	$1.8*10^{-4}$ p	Modelled from Ecoinvent v 3.8 database sub process as detailed in Table S14[13]
		Aspiration system	$2.38*10^{-4}$ p	Modelled from Ecoinvent v 3.8 database sub process as detailed in Table S8[14]
		Activated carbon air filter	$9.5*10^{-4}$ p	Modelled from Ecoinvent v 3.8 database sub process as detailed in Table S9[15]
	Transports	Transport of raw materials	35.29 kgkm	Ecoinvent v3.8[16]
		Transport of small equipment	0.395 kgkm	Ecoinvent v 3.8[17]
		Transport of large equipment/plants	$3.96*10^{-2}$ tkm	Ecoinvent v 3.8[18]
	Energy	Electric Energy	900 W	Ecoinvent v 3.8[19]
		Electric Energy	12.93 kJ	Ecoinvent v 3.8[20]
		Electric Energy	1.59 kWh	Ecoinvent v 3.8[21]
Output	Avoided Products	Diethyl ether	33.64 g	Ecoinvent v 3.8[22]
	Emission to air	Ethylene glycol	$1.72*10^{-8}$ g	SimaPro airborne emission substance list[23]
		Diethyl ether	$8.78*10^{-4}$ g	SimaPro airborne emission substance list[24]
		H_2O	$6.22*10^{-5}$ g	SimaPro airborne emission substance list[25]
	End of Life	Spent Solvent Mixture	317.84 g	Ecoinvent v 3.8[26]
		Spent Solvent Mixture	$2.62*10^{-3}$ g	Ecoinvent v 3.8[27]

[1]Carboxylic acid used to start the synthesis of the precursor 2E. The Ecoinvent process used was: Phenyl acetic acid {RER}| production | APOS, U.

[2]The Ecoinvent process used was: Ethylene glycol {RER}| production | APOS, U.

[3]Sodium carbonate was used during the work up procedure. This material wasn't available on the Ecoinvent database, so Potassium Carbonate was chosen instead. The Ecoinvent process used was: Potassium carbonate {GLO}| production, from potassium hydroxide | APOS, U.

[4]Water necessary for the preparation of the solution of diethyl ether. The Ecoinvent process used was: Water, deionised {Europe without Switzerland} | water production, deionised | APOS, U.
[5]Diethyl ether used during the workup procedure for the extraction of the organic phase. The Ecoinvent process used was: Diethyl ether, without water, in 99.95% solution state {RoW}| ethylene hydration | APOS, U.
[6]Water used for the washing of the organic phase, after the extraction with diethyl ether, in order to remove the residual catalyst. The Ecoinvent process used was: Water, deionised {Europe without Switzerland} | water production, deionised | APOS, U.
[7]Sodium chloride used to wash the organic phase to remove unreacted reagents. The Ecoinvent process used was: Sodium chloride, brine solution {RER}| production | APOS, U.
[8]Sodium sulphate anhydrous used to remove the residual water in the organic phase. The Ecoinvent process used was: Sodium sulphate, anhydrite {RER}| Mannheim process | APOS, U.
[9]The Ecoinvet process used was: Silicone product {RER}| production | APOS, U.
[10]The reported value was calculated by the following formula: 1/28800h*18h, where 28800 h is the lifetime of the round bottomed flask and 18h is the time of usage of the round bottomed flask.
[11]The reported value was calculated by the following formula: 1/57600h*18h, where 57600 h is the lifetime of the silicone oil container and 18h is its time of usage.
[12]The reported value was calculated by the following formula: 1/50000h*18h, where 50000 h is the lifetime of the magnetic stirrer and 18h is the time of usage of the magnetic stirrer.
[13]The reported value was calculated by the following formula: 1/100000h*18h, where 100000 h is the lifetime of the stir bar and 18h is its time of usage.
[14]The reported value was calculated by the following formula: 1/79750h*19h, where 79750 h is the lifetime of the aspiration system and 19h is its time of usage. One additional hour of functioning was considered for the work-up procedure.
[15]The reported value was calculated by the following formula: 1/20000h*19h, where 20000 h is the lifetime of the activated carbon air filter and 19h is its time of usage. One additional hour of functioning was considered for the work-up procedure.
[16]Transport of raw materials (reagents). The Ecoinvent process used was: Transport, freight, lorry 3.5-7.5 metric ton, EURO6 {RER}| transport, freight, lorry 3.5-7.5 metric ton, EURO6 | APOS, U. An average distance of 100 km was considered.
[17]Transport of small equipment. The Ecoinvent process used was: Transport, freight, lorry 3.5-7.5 metric ton, EURO6 {RER}| transport, freight, lorry 3.5-7.5 metric ton, EURO6 | APOS, U. an average distance of 100 km was considered.
[18]Transport of large equipment (aspiration system and activated carbon air filter). The Ecoinvent process used was: Transport, freight, lorry 16-32 metric ton, euro6 {RER}| market for transport, freight, lorry 16-32 metric ton, EURO6 | APOS, U. an average distance of 100 km was considered.
[19]Electric energy necessary to heat up the reaction mixture at 75°C. It was calculated as power*time, considering the reaction time to be 18 hours and the power of heating/magnetic stirrer to be 500W. The Ecoinvent process used was: Electricity, low voltage {IT}| electricity voltage transformation from medium to low voltage | APOS, U.
[20]Electric energy necessary to evaporate diethyl ether, calculated using the vaporization enthalpy of the substance, 27 kJ/mol. The Ecoinvent process used was: Electricity, low voltage {IT}| electricity voltage transformation from medium to low voltage | APOS, U..
[21]Electric energy necessary to the use of the aspiration system for the whole synthesis time, i.e. 19 h. The power was calculated by considering the air flow rate of 250 m^3/h, a total load loss of 110.8076 kg/m^2, and an efficiency of 90%. The Ecoinvent process used was: Electricity, low voltage {IT}| electricity voltage transformation from medium to low voltage | APOS, U.
[22]Avoided Products, it is assumed that 95% of Diethyl ether can be recovered. The Ecoinvent process used was: Diethyl ether, without water, in 99.95% solution state {RoW}| ethylene hydration | APOS, U.
[23]Amount of ethylene glycol released into the atmosphere, as calculated by the formula reported in equation 1 of the main manuscript.
[24]Amount of Diethyl ether released into the atmosphere, as calculated by the formula reported in equation 1 of the main manuscript.
[25]Amount of Water released into the atmosphere, as calculated by the formula reported in equation 1 of the main manuscript.
[26]End of life treatment for what was used during the synthesis, work up procedure and isolation of the final product. The Ecoinvent process used was: Spent solvent mixture {Europe without Switzerland}| treatment of spent solvent mixture, hazardous waste incineration, with energy recovery | APOS, U.
[27]End of life for the silicon oil. Ecoinvent process used was: Spent solvent mixture {Europe without Switzerland}| treatment of spent solvent mixture, hazardous waste incineration, with energy recovery | APOS, U.

Table S31. Contributions for the Life Cycle Inventory (LCI) for the production of 0.83 g of diester derivative **3a** according to the procedure detailed in [11].

Description			Amount	Process Data Source
Input	Materials			
		hydroxyalkyl ester 2a	0.82 g	Modelled from Ecoinvent v 3.8 database sub process as detailed in Table S26
		Levulinic Acid	1.04 g	Modelled from Ecoinvent v 3.8 database sub process as detailed in Table S20
		Toluene	17.4 g	Ecoinvent v 3.8[1]
		H_2SO_4	0.092g	Ecoinvent v 3.8[2]
		K_2CO_3	10 g	Ecoinvent v 3.8[3]
		H_2O	90 g	Ecoinvent v 3.8[4]
		Diethyl ether	35.5 g	Ecoinvent v 3.8[5]
		H_2O	150 g	Ecoinvent v 3.8[6]
		NaCl	50 g	Ecoinvent v 3.8[7]
		Na_2SO_4	10 g	Ecoinvent v 3.8[8]
		Silicon Oil	$1.02*10^{-3}$ kg	Ecoinvent v 3.8[9]
	Equipment/plants	Two neck round bottom flask	$2.43*10^{-4}$ p	Modelled from Ecoinvent v 3.8 database sub process as detailed in Table S21[10]
		Silicon oil container	$1.22*10^{-4}$ p	Modelled from Ecoinvent v 3.8 database sub process as detailed in Table S13[11]
		Magnetic stirrer	$1.4*10^{-4}$ p	Modelled from Ecoinvent v 3.8 database sub process as detailed in Table S15[12]
		Magnetic stirr bar	$7*10^{-5}$ p	Modelled from Ecoinvent v 3.8 database sub process as detailed in Table S14[13]
		Aspiration system	$1*10^{-4}$ p	Modelled from Ecoinvent v 3.8 database sub process as detailed in Table S8[14]
		Activated carbon air filter	$4*10^{-4}$ p	Modelled from Ecoinvent v 3.8 database sub process as detailed in Table S9[15]
	Transports	Transport of raw materials	36.40 kgkm	Ecoinvent v3.8[16]
		Transport of small equipment	0.154 kgkm	Ecoinvent v 3.8[17]
		Transport of large equipment	$1.67*10^{-2}$ tkm	Ecoinvent v 3.8[18]
	Energy	Electric Energy	3500 Wh	Ecoinvent v 3.8[19]
		Electric Energy	20.11 kJ	Ecoinvent v 3.8[20]
		Electric Energy	0.67 kW	Ecoinvent v 3.8[21]

Output	Avoided Products	Diethyl ether	33.64 g	Ecoinvent v 3.8[22]
		Toluene	13.92 g	Ecoinvent v 3.8[23]
	Emission to air	Toluene	$2.82*10^{-5}$ g	SimaPro airborne emission substance list[24]
		Diethyl ether	$8.78*10^{-4}$ g	SimaPro airborne emission substance list[25]
		Sulfuric Acid	$2.64*10^{-12}$ g	SimaPro airborne emission substance list[26]
		Water	$5.53*10^{-5}$ g	SimaPro airborne emission substance list[27]
	End of Life	Spent Solvent Mixture	316.28 g	Ecoinvent v 3.8[28]
		Spent Solvent Mixture	0.0010 kg	Ecoinvent v 3.8[29]

[1]The Ecoinvent process used was: Toluene, liquid {RER}| production | APOS, U.
2Drop of Sulfuric Acid (96%) used. The Ecoinvent process used was: Sulfuric acid {RER}| production | APOS, U.
[3]Sodium carbonate was used during the work up procedure but this material wasn't available on the Ecoinvent database, so Potassium Carbonate was chosen instead. The Ecoinvent process used was: Potassium carbonate {GLO}| production, from potassium hydroxide | APOS, U.
[4]Water necessary for the preparation of the solution of diethyl ether. The Ecoinvent process used was: Water, deionised {Europe without Switzerland} | water production, deionised | APOS, U.
[5]Diethyl ether used during the workup procedure for the extraction of the organic phase. The Ecoinvent process used was: Diethyl ether, without water, in 99.95% solution state {RoW}| ethylene hydration | APOS, U.
[6]Water used for the washing of the organic phase, after the extraction with diethyl ether, in order to remove the residual catalyst. The Ecoinvent process used was: Water, deionised {Europe without Switzerland} | water production, deionised | APOS, U.
[7]Sodium chloride used to wash the organic phase to remove unreacted reagents. The Ecoinvent process used was: Sodium chloride, brine solution {RER}| production | APOS, U.
[8]Sodium sulphate anhydrous used to remove the residual water in the organic phase. The Ecoinvent process used was: Sodium sulphate, anhydrite {RER}| Mannheim process | APOS, U.
[9]The Ecoinvent process used was: Silicone product {RER}| production | APOS, U
[10]The reported value was calculated by the following formula: 1/28800h*7h, where 28800 h is the lifetime of the two neck round bottomed flask and 7h is its time of usage.
[11]The reported value was calculated by the following formula: 1/57600h*7h, where 57600 h is the lifetime of the silicone oil container and 7h is its time of usage.
[12]The reported value was calculated by the following formula: 1/50000h*7h, where 50000 h is the lifetime of the magnetic stirrer and 7h is the time of usage of the magnetic stirrer.
[13]The reported value was calculated by the following formula: 1/100000h*7h, where 100000 h is the lifetime of the stir bar and 7h is its time of usage.
[14]The reported value was calculated by the following formula: 1/79750h*8h, where 79750 h is the lifetime of the aspiration system and 8h is its time of usage. One additional hour of functioning was considered for the work-up procedure.
[15]The reported value was calculated by the following formula: 1/20000h*8h, where 20000 h is the lifetime of the activated carbon air filter and 8h is its time of usage. One additional hour of functioning was considered for the work-up procedure.
[16]Transport of raw materials (reagents). The Ecoinvent process used was: Transport, freight, lorry 3.5-7.5 metric ton, EURO6 {RER}| transport, freight, lorry 3.5-7.5 metric ton, EURO6 | APOS, U. An average distance of 100 km was considered.
[17]Transport of small equipment. The Ecoinvent process used was: Transport, freight, lorry 3.5-7.5 metric ton, EURO6 {RER}| transport, freight, lorry 3.5-7.5 metric ton, EURO6 | APOS, U. an average distance of 100 km was considered.
[18]Transport of large equipment (aspiration system and activated carbon air filter). The Ecoinvent process used was: Transport, freight, lorry 16-32 metric ton, euro6 {RER}| market for transport, freight, lorry 16-32 metric ton, EURO6 | APOS, U. an average distance of 100 km was considered.

[19]Electric energy necessary to heat the reaction mixture at 140°C. It was calculated using power*time, considering a power of 500W for the heating/magnetic stirrer and a reaction time of seven hours. The Ecoinvent process used was: Electricity, low voltage {IT}| electricity voltage transformation from medium to low voltage | APOS, U.
[20]Electric energy necessary to evaporate diethyl ether and toluene, calculated using the vaporization enthalpy for each substance, which are respectively 27 kJ/mol and 38 kJ/mol. The Ecoinvent process used was: Electricity, low voltage {IT}| electricity voltage transformation from medium to low voltage | APOS, U.
[21]Electric energy necessary to the use of the aspiration system for the whole synthesis time, i.e. 9 h. The power was calculated by considering the air flow rate of 250 m^3/h, a total load loss of 110.8076 kg/m^2, and an efficiency of 90%. The Ecoinvent process used was: Electricity, low voltage {IT}| electricity voltage transformation from medium to low voltage | APOS, U.
[22]Avoided Products, it is assumed that 95% of Diethyl ether can be recovered. The Ecoinvent process used was: Diethyl ether, without water, in 99.95% solution state {RoW}| ethylene hydration | APOS, U.
[23]Avoided Products, it is assumed that 80% of Toluene can be recovered. The Ecoinvent process used was: Toluene, liquid {RER}| production | APOS, U.
[24]Amount of toluene released into the atmosphere, as calculated by the formula reported in equation 1 of the main manuscript.
[25]Amount of Diethyl ether released into the atmosphere, as calculated by the formula reported in equation 1 of the main manuscript.
[26]Amount of Sulfuric Acid released into the atmosphere, as calculated by the formula reported in equation 1 of the main manuscript.
[27]Amount of Water released into the atmosphere, as calculated by the formula reported in equation 1 of the main manuscript.
[28]End of life treatment for what used during the synthesis, work up procedure and isolation of the final product. The Ecoinvent process used was: Spent solvent mixture {Europe without Switzerland}| treatment of spent solvent mixture, hazardous waste incineration, with energy recovery | APOS, U.
[29]End of life treatment for the Silicon oil. The Ecoinvent process used was: Spent solvent mixture {Europe without Switzerland}| treatment of spent solvent mixture, hazardous waste incineration, with energy recovery | APOS, U.

Table S32. Contributions for the Life Cycle Inventory (LCI) for the production of 1.16 g of diester derivative **3b** according to the procedure detailed in [11].

Description			Amount	Process Data Source
Input	Materials			
		hydroxyalkyl ester 2b	0.99 g	Modelled from Ecoinvent v 3.8 database sub process as detailed in Table S27
		Levulinic Acid	1.04 g	Modelled from Ecoinvent v 3.8 database sub process as detailed in Table S20
		Toluene	17.4 g	Ecoinvent v 3.8[1]
		H_2SO_4	0.092g	Ecoinvent v 3.8[2]
		K_2CO_3	10 g	Ecoinvent v 3.8[3]
		H_2O	90 g	Ecoinvent v 3.8[4]
		Diethyl ether	35.5 g	Ecoinvent v 3.8[5]
		H_2O	150 g	Ecoinvent v 3.8[6]
		NaCl	50 g	Ecoinvent v 3.8[7]
		Na_2SO_4	10 g	Ecoinvent v 3.8[8]
		Silicon Oil	$1.02*10^{-3}$ kg	Ecoinvent v 3.8[9]

	Equipment/plants	Two neck round bottom flask	$2.43*10^{-4}$ p	Modelled from Ecoinvent v 3.8 database sub process as detailed in Table S21[10]
		Silicon oil container	$1.22*10^{-4}$ p	Modelled from Ecoinvent v 3.8 database sub process as detailed in Table S13[11]
		Magnetic stirrer	$1.4*10^{-4}$ p	Modelled from Ecoinvent v 3.8 database sub process as detailed in Table S15[12]
		Magnetic stirr bar	$7*10^{-5}$ p	Modelled from Ecoinvent v 3.8 database sub process as detailed in Table S14[13]
		Aspiration system	$1*10^{-4}$ p	Modelled from Ecoinvent v 3.8 database sub process as detailed in Table S8[14]
		Activated carbon air filter	$4*10^{-4}$ p	Modelled from Ecoinvent v 3.8 database sub process as detailed in Table S9[15]
	Transports	Transport of raw materials	36.40 kgkm	Ecoinvent v3.8[16]
		Transport of small equipment	0.154 kgkm	Ecoinvent v 3.8[17]
		Transport of large equipment	$1.67*10^{-2}$ tkm	Ecoinvent v 3.8[18]
	Energy	Electric Energy	3500 Wh	Ecoinvent v 3.8[19]
		Electric Energy	20.11 kJ	Ecoinvent v 3.8[20]
		Electric Energy	0.67 kW	Ecoinvent v 3.8[21]
Output	Avoided Products	Diethyl ether	33.64 g	Ecoinvent v 3.8[22]
		Toluene	13.92 g	Ecoinvent v 3.8[23]
	Emission to air	Toluene	$2.82*10^{-5}$ g	SimaPro airborne emission substance list[24]
		Diethyl ether	$8.78*10^{-4}$ g	SimaPro airborne emission substance list[25]
		Sulfuric Acid	$2.64*10^{-12}$ g	SimaPro airborne emission substance list[26]
		Water	$5.53*10^{-5}$ g	SimaPro airborne emission substance list[27]
	End of Life	Spent Solvent Mixture	316.12 g	Ecoinvent v 3.8[28]
		Spent Solvent Mixture	0.0010 kg	Ecoinvent v 3.8[29]

[1]The Ecoinvent process used was: Toluene, liquid {RER}| production | APOS, U.
2Drop of Sulfuric Acid (96%) used. The Ecoinvent process used was: Sulfuric acid {RER}| production | APOS, U.

[3]Sodium carbonate was used during the work up procedure but this material wasn't available on the Ecoinvent database, so Potassium Carbonate was chosen instead. The Ecoinvent process used was: Potassium carbonate {GLO}| production, from potassium hydroxide | APOS, U.
[4]Water necessary for the preparation of the solution of diethyl ether. The Ecoinvent process used was: Water, deionised {Europe without Switzerland} | water production, deionised | APOS, U.
[5]Diethyl ether used during the workup procedure for the extraction of the organic phase. The Ecoinvent process used was: Diethyl ether, without water, in 99.95% solution state {RoW}| ethylene hydration | APOS, U.
[6]Water used for the washing of the organic phase, after the extraction with diethyl ether, in order to remove the residual catalyst. The Ecoinvent process used was: Water, deionised {Europe without Switzerland} | water production, deionised | APOS, U.
[7]Sodium chloride used to wash the organic phase to remove unreacted reagents. The Ecoinvent process used was: Sodium chloride, brine solution {RER}| production | APOS, U.
[8]Sodium sulphate anhydrous used to remove the residual water in the organic phase. The Ecoinvent process used was: Sodium sulphate, anhydrite {RER}| Mannheim process | APOS, U.
[9]The Ecoinvent process used was: Silicone product {RER}| production | APOS, U
[10]The reported value was calculated by the following formula: 1/28800h*7h, where 28800 h is the lifetime of the two neck round bottomed flask and 7h is its time of usage.
[11]The reported value was calculated by the following formula: 1/57600h*7h, where 57600 h is the lifetime of the silicone oil container and 7h is its time of usage.
[12]The reported value was calculated by the following formula: 1/50000h*7h, where 50000 h is the lifetime of the magnetic stirrer and 7h is the time of usage of the magnetic stirrer.
[13]The reported value was calculated by the following formula: 1/100000h*7h, where 100000 h is the lifetime of the stir bar and 7h is its time of usage.
[14]The reported value was calculated by the following formula: 1/79750h*8h, where 79750 h is the lifetime of the aspiration system and 8h is its time of usage. One additional hour of functioning was considered for the work-up procedure.
[15]The reported value was calculated by the following formula: 1/20000h*8h, where 20000 h is the lifetime of the activated carbon air filter and 8h is its time of usage. One additional hour of functioning was considered for the work-up procedure.
[16]Transport of raw materials (reagents). The Ecoinvent process used was: Transport, freight, lorry 3.5-7.5 metric ton, EURO6 {RER}| transport, freight, lorry 3.5-7.5 metric ton, EURO6 | APOS, U. An average distance of 100 km was considered.
[17]Transport of small equipment. The Ecoinvent process used was: Transport, freight, lorry 3.5-7.5 metric ton, EURO6 {RER}| transport, freight, lorry 3.5-7.5 metric ton, EURO6 | APOS, U. an average distance of 100 km was considered.
[18]Transport of large equipment (aspiration system and activated carbon air filter). The Ecoinvent process used was: Transport, freight, lorry 16-32 metric ton, euro6 {RER}| market for transport, freight, lorry 16-32 metric ton, EURO6 | APOS, U. an average distance of 100 km was considered.
[19]Electric energy necessary to heat the reaction mixture at 140°C. It was calculated using power*time, considering a power of 500W for the heating/magnetic stirrer and a reaction time of seven hours. The Ecoinvent process used was: Electricity, low voltage {IT}| electricity voltage transformation from medium to low voltage | APOS, U.
[20]Electric energy necessary to evaporate diethyl ether and toluene, calculated using the vaporization enthalpy for each substance, which are respectively 27 kJ/mol and 38 kJ/mol. The Ecoinvent process used was: Electricity, low voltage {IT}| electricity voltage transformation from medium to low voltage | APOS, U.
[21]Electric energy necessary to the use of the aspiration system for the whole synthesis time, i.e. 9 h. The power was calculated by considering the air flow rate of 250 m^3/h, a total load loss of 110.8076 kg/m^2, and an efficiency of 90%. The Ecoinvent process used was: Electricity, low voltage {IT}| electricity voltage transformation from medium to low voltage | APOS, U.
[22]Avoided Products, it is assumed that 95% of Diethyl ether can be recovered. The Ecoinvent process used was: Diethyl ether, without water, in 99.95% solution state {RoW}| ethylene hydration | APOS, U.
[23]Avoided Products, it is assumed that 80% of Toluene can be recovered. The Ecoinvent process used was: Toluene, liquid {RER}| production | APOS, U.
[24]Amount of toluene released into the atmosphere, as calculated by the formula reported in equation 1 of the main manuscript.
[25]Amount of Diethyl ether released into the atmosphere, as calculated by the formula reported in equation 1 of the main manuscript.
[26]Amount of Sulfuric Acid released into the atmosphere, as calculated by the formula reported in equation 1 of the main manuscript.
[27]Amount of Water released into the atmosphere, as calculated by the formula reported in equation 1 of the main manuscript.
[28]End of life treatment for what used during the synthesis, work up procedure and isolation of the final product. The Ecoinvent process used was: Spent solvent mixture {Europe without Switzerland}| treatment of spent solvent mixture, hazardous waste incineration, with energy recovery | APOS, U.

[29]End of life treatment for the Silicon oil. The Ecoinvent process used was: Spent solvent mixture {Europe without Switzerland}| treatment of spent solvent mixture, hazardous waste incineration, with energy recovery | APOS, U.

Table S33. Contributions for the Life Cycle Inventory (LCI) for the production of 0.69 g of diester derivative **3c** according to the procedure detailed in [11].

Description			Amount	Process Data Source
Input	Materials			
		hydroxyalkyl ester 2c	0.44 g	Modelled from Ecoinvent v 3.8 database sub process as detailed in Table S28
		Levulinic Acid	1.04 g	Modelled from Ecoinvent v 3.8 database sub process as detailed in Table S20
		Toluene	17.4 g	Ecoinvent v 3.8[1]
		H_2SO_4	0.092g	Ecoinvent v 3.8[2]
		K_2CO_3	10 g	Ecoinvent v 3.8[3]
		H_2O	90 g	Ecoinvent v 3.8[4]
		Diethyl ether	35.5 g	Ecoinvent v 3.8[5]
		H_2O	150 g	Ecoinvent v 3.8[6]
		NaCl	50 g	Ecoinvent v 3.8[7]
		Na_2SO_4	10 g	Ecoinvent v 3.8[8]
		Silicon Oil	$1.02*10^{-3}$ kg	Ecoinvent v 3.8[9]
	Equipment/plants	Two neck round bottom flask	$2.43*10^{-4}$ p	Modelled from Ecoinvent v 3.8 database sub process as detailed in Table S21
		Silicon oil container	$1.22*10^{-4}$ p	Modelled from Ecoinvent v 3.8 database sub process as detailed in Table S13
		Magnetic stirrer	$1.4*10^{-4}$ p	Modelled from Ecoinvent v 3.8 database sub process as detailed in Table S15
		Magnetic stirr bar	$7*10^{-5}$ p	Modelled from Ecoinvent v 3.8 database sub process as detailed in Table S14
		Aspiration system	$1*10^{-4}$ p	Modelled from Ecoinvent v 3.8 database sub process as detailed in Table S8
		Activated carbon air filter	$4*10^{-4}$ p	Modelled from Ecoinvent v 3.8 database sub process as detailed in Table S9
	Transports	Transport of raw materials	36.40 kgkm	Ecoinvent v3.8[10]
		Transport of small equipment	0.154 kgkm	Ecoinvent v 3.8[11]
		Transport of large equipment	$1.67*10^{-2}$ tkm	Ecoinvent v 3.8[12]

	Energy	Electric Energy	3500 Wh	Ecoinvent v 3.8[13]
		Electric Energy	20.11 kJ	Ecoinvent v 3.8[14]
		Electric Energy	0.67 kW	Ecoinvent v 3.8[15]
Output	Avoided Products	Diethyl ether	33.64 g	Ecoinvent v 3.8[16]
		Toluene	13.92 g	Ecoinvent v 3.8[17]
	Emission to air	Toluene	$2.82*10^{-5}$ g	SimaPro airborne emission substance list[18]
		Diethyl ether	$8.78*10^{-4}$ g	SimaPro airborne emission substance list[19]
		Sulfuric Acid	$2.64*10^{-12}$ g	SimaPro airborne emission substance list[20]
		Water	$5.53*10^{-5}$ g	SimaPro airborne emission substance list[21]
	End of Life	Spent Solvent Mixture	316.04 g	Ecoinvent v 3.8[22]
		Spent Solvent Mixture	0.0010 kg	Ecoinvent v 3.8[23]

[1]The Ecoinvent process used was: Toluene, liquid {RER}| production | APOS, U.
2Drop of Sulfuric Acid (96%) used. The Ecoinvent process used was: Sulfuric acid {RER}| production | APOS, U.
[3]Sodium carbonate was used during the work up procedure but this material wasn't available on the Ecoinvent database, so Potassium Carbonate was chosen instead. The Ecoinvent process used was: Potassium carbonate {GLO}| production, from potassium hydroxide | APOS, U.
[4]Water necessary for the preparation of the solution of diethyl ether. The Ecoinvent process used was: Water, deionised {Europe without Switzerland} | water production, deionised | APOS, U.
[5]Diethyl ether used during the workup procedure for the extraction of the organic phase. The Ecoinvent process used was: Diethyl ether, without water, in 99.95% solution state {RoW}| ethylene hydration | APOS, U.
[6]Water used for the washing of the organic phase, after the extraction with diethyl ether, in order to remove the residual catalyst. The Ecoinvent process used was: Water, deionised {Europe without Switzerland} | water production, deionised | APOS, U.
[7]Sodium chloride used to wash the organic phase to remove unreacted reagents. The Ecoinvent process used was: Sodium chloride, brine solution {RER}| production | APOS, U.
[8]Sodium sulphate anhydrous used to remove the residual water in the organic phase. The Ecoinvent process used was: Sodium sulphate, anhydrite {RER}| Mannheim process | APOS, U.
[9]The Ecoinvent process used was: Silicone product {RER}| production | APOS, U
[10]The reported value was calculated by the following formula: 1/28800h*7h, where 28800 h is the lifetime of the two neck round bottomed flask and 7h is its time of usage.
[11]The reported value was calculated by the following formula: 1/57600h*7h, where 57600 h is the lifetime of the silicone oil container and 7h is its time of usage.
[12]The reported value was calculated by the following formula: 1/50000h*7h, where 50000 h is the lifetime of the magnetic stirrer and 7h is the time of usage of the magnetic stirrer.
[13]The reported value was calculated by the following formula: 1/100000h*7h, where 100000 h is the lifetime of the stir bar and 7h is its time of usage.
[14]The reported value was calculated by the following formula: 1/79750h*8h, where 79750 h is the lifetime of the aspiration system and 8h is its time of usage. One additional hour of functioning was considered for the work-up procedure.
[15]The reported value was calculated by the following formula: 1/20000h*8h, where 20000 h is the lifetime of the activated carbon air filter and 8h is its time of usage. One additional hour of functioning was considered for the work-up procedure.
[16]Transport of raw materials (reagents). The Ecoinvent process used was: Transport, freight, lorry 3.5-7.5 metric ton, EURO6 {RER}| transport, freight, lorry 3.5-7.5 metric ton, EURO6 | APOS, U. An average distance of 100 km was considered.

[17]Transport of small equipment. The Ecoinvent process used was: Transport, freight, lorry 3.5-7.5 metric ton, EURO6 {RER}| transport, freight, lorry 3.5-7.5 metric ton, EURO6 | APOS, U. an average distance of 100 km was considered.
[18]Transport of large equipment (aspiration system and activated carbon air filter). The Ecoinvent process used was: Transport, freight, lorry 16-32 metric ton, euro6 {RER}| market for transport, freight, lorry 16-32 metric ton, EURO6 | APOS, U. an average distance of 100 km was considered.
[19]Electric energy necessary to heat the reaction mixture at 140°C. It was calculated using power*time, considering a power of 500W for the heating/magnetic stirrer and a reaction time of seven hours. The Ecoinvent process used was: Electricity, low voltage {IT}| electricity voltage transformation from medium to low voltage | APOS, U.
[20]Electric energy necessary to evaporate diethyl ether and toluene, calculated using the vaporization enthalpy for each substance, which are respectively 27 kJ/mol and 38 kJ/mol. The Ecoinvent process used was: Electricity, low voltage {IT}| electricity voltage transformation from medium to low voltage | APOS, U.
[21]Electric energy necessary to the use of the aspiration system for the whole synthesis time, i.e. 9 h. The power was calculated by considering the air flow rate of 250 m^3/h, a total load loss of 110.8076 kg/m^2, and an efficiency of 90%. The Ecoinvent process used was: Electricity, low voltage {IT}| electricity voltage transformation from medium to low voltage | APOS, U.
[22]Avoided Products, it is assumed that 95% of Diethyl ether can be recovered. The Ecoinvent process used was: Diethyl ether, without water, in 99.95% solution state {RoW}| ethylene hydration | APOS, U.
[23]Avoided Products, it is assumed that 80% of Toluene can be recovered. The Ecoinvent process used was: Toluene, liquid {RER}| production | APOS, U.
[24]Amount of toluene released into the atmosphere, as calculated by the formula reported in equation 1 of the main manuscript.
[25]Amount of Diethyl ether released into the atmosphere, as calculated by the formula reported in equation 1 of the main manuscript.
[26]Amount of Sulfuric Acid released into the atmosphere, as calculated by the formula reported in equation 1 of the main manuscript.
[27]Amount of Water released into the atmosphere, as calculated by the formula reported in equation 1 of the main manuscript.
[28]End of life treatment for what used during the synthesis, work up procedure and isolation of the final product. The Ecoinvent process used was: Spent solvent mixture {Europe without Switzerland}| treatment of spent solvent mixture, hazardous waste incineration, with energy recovery | APOS, U.
[29]End of life treatment for the Silicon oil. The Ecoinvent process used was: Spent solvent mixture {Europe without Switzerland}| treatment of spent solvent mixture, hazardous waste incineration, with energy recovery | APOS, U.

Table S34. Contributions for the Life Cycle Inventory (LCI) for the production of 0.78 g of diester derivative **3d** according to the procedure detailed in [11].

		Description	Amount	Process Data Source
Input	Materials			
		hydroxyalkyl ester 2d	0.47 g	Modelled from Ecoinvent v 3.8 database sub process as detailed in Table S29
		Levulinic Acid	1.04 g	Modelled from Ecoinvent v 3.8 database sub process as detailed in Table S20
		Toluene	17.4 g	Ecoinvent v 3.8[1]
		H_2SO_4	0.092g	Ecoinvent v 3.8[2]
		K_2CO_3	10 g	Ecoinvent v 3.8[3]
		H_2O	90 g	Ecoinvent v 3.8[4]
		Diethyl ether	35.5 g	Ecoinvent v 3.8[5]
		H_2O	150 g	Ecoinvent v 3.8[6]
		NaCl	50 g	Ecoinvent v 3.8[7]
		Na_2SO_4	10 g	Ecoinvent v 3.8[8]

		Silicon Oil	$1.02*10^{-3}$ kg	Ecoinvent v 3.8[9]
	Equipment/plants	Two neck round bottom flask	$2.43*10^{-4}$ p	Modelled from Ecoinvent v 3.8 database sub process as detailed in Table S21[10]
		Silicon oil container	$1.22*10^{-4}$ p	Modelled from Ecoinvent v 3.8 database sub process as detailed in Table S13[11]
		Magnetic stirrer	$1.4*10^{-4}$ p	Modelled from Ecoinvent v 3.8 database sub process as detailed in Table S15[12]
		Magnetic stirr bar	$7*10^{-5}$ p	Modelled from Ecoinvent v 3.8 database sub process as detailed in Table S14[13]
		Aspiration system	$1*10^{-4}$ p	Modelled from Ecoinvent v 3.8 database sub process as detailed in Table S8[14]
		Activated carbon air filter	$4*10^{-4}$ p	Modelled from Ecoinvent v 3.8 database sub process as detailed in Table S9[15]
	Transports	Transport of raw materials	36.40 kgkm	Ecoinvent v3.8[16]
		Transport of small equipment	0.154 kgkm	Ecoinvent v 3.8[17]
		Transport of large equipment	$1.67*10^{-2}$ tkm	Ecoinvent v 3.8[18]
	Energy	Electric Energy	3500 Wh	Ecoinvent v 3.8[19]
		Electric Energy	20.11 kJ	Ecoinvent v 3.8[20]
		Electric Energy	0.67 kW	Ecoinvent v 3.8[21]
Output	Avoided Products	Diethyl ether	33.64 g	Ecoinvent v 3.8[22]
		Toluene	13.92 g	Ecoinvent v 3.8[23]
	Emission to air	Toluene	$2.82*10^{-5}$ g	SimaPro airborne emission substance list[24]
		Diethyl ether	$8.78*10^{-4}$ g	SimaPro airborne emission substance list[25]
		Sulfuric Acid	$2.64*10^{-12}$ g	SimaPro airborne emission substance list[26]
		Water	$5.53*10^{-5}$ g	SimaPro airborne emission substance list[27]
	End of Life	Spent Solvent Mixture	315.99 g	Ecoinvent v 3.8[28]
		Spent Solvent Mixture	0.0010 kg	Ecoinvent v 3.8[29]

[1]The Ecoinvent process used was: Toluene, liquid {RER}| production | APOS, U.
2Drop of Sulfuric Acid (96%) used. The Ecoinvent process used was: Sulfuric acid {RER}| production | APOS, U.

[3]Sodium carbonate was used during the work up procedure but this material wasn't available on the Ecoinvent database, so Potassium Carbonate was chosen instead. The Ecoinvent process used was: Potassium carbonate {GLO}| production, from potassium hydroxide | APOS, U.
[4]Water necessary for the preparation of the solution of diethyl ether. The Ecoinvent process used was: Water, deionised {Europe without Switzerland} | water production, deionised | APOS, U.
[5]Diethyl ether used during the workup procedure for the extraction of the organic phase. The Ecoinvent process used was: Diethyl ether, without water, in 99.95% solution state {RoW}| ethylene hydration | APOS, U.
[6]Water used for the washing of the organic phase, after the extraction with diethyl ether, in order to remove the residual catalyst. The Ecoinvent process used was: Water, deionised {Europe without Switzerland} | water production, deionised | APOS, U.
[7]Sodium chloride used to wash the organic phase to remove unreacted reagents. The Ecoinvent process used was: Sodium chloride, brine solution {RER}| production | APOS, U.
[8]Sodium sulphate anhydrous used to remove the residual water in the organic phase. The Ecoinvent process used was: Sodium sulphate, anhydrite {RER}| Mannheim process | APOS, U.
[9]The Ecoinvent process used was: Silicone product {RER}| production | APOS, U
[10]The reported value was calculated by the following formula: 1/28800h*7h, where 28800 h is the lifetime of the two neck round bottomed flask and 7h is its time of usage.
[11]The reported value was calculated by the following formula: 1/57600h*7h, where 57600 h is the lifetime of the silicone oil container and 7h is its time of usage.
[12]The reported value was calculated by the following formula: 1/50000h*7h, where 50000 h is the lifetime of the magnetic stirrer and 7h is the time of usage of the magnetic stirrer.
[13]The reported value was calculated by the following formula: 1/100000h*7h, where 100000 h is the lifetime of the stir bar and 7h is its time of usage.
[14]The reported value was calculated by the following formula: 1/79750h*8h, where 79750 h is the lifetime of the aspiration system and 8h is its time of usage. One additional hour of functioning was considered for the work-up procedure.
[15]The reported value was calculated by the following formula: 1/20000h*8h, where 20000 h is the lifetime of the activated carbon air filter and 8h is its time of usage. One additional hour of functioning was considered for the work-up procedure.
[16]Transport of raw materials (reagents). The Ecoinvent process used was: Transport, freight, lorry 3.5-7.5 metric ton, EURO6 {RER}| transport, freight, lorry 3.5-7.5 metric ton, EURO6 | APOS, U. An average distance of 100 km was considered.
[17]Transport of small equipment. The Ecoinvent process used was: Transport, freight, lorry 3.5-7.5 metric ton, EURO6 {RER}| transport, freight, lorry 3.5-7.5 metric ton, EURO6 | APOS, U. an average distance of 100 km was considered.
[18]Transport of large equipment (aspiration system and activated carbon air filter). The Ecoinvent process used was: Transport, freight, lorry 16-32 metric ton, euro6 {RER}| market for transport, freight, lorry 16-32 metric ton, EURO6 | APOS, U. an average distance of 100 km was considered.
[19]Electric energy necessary to heat the reaction mixture at 140°C. It was calculated using power*time, considering a power of 500W for the heating/magnetic stirrer and a reaction time of seven hours. The Ecoinvent process used was: Electricity, low voltage {IT}| electricity voltage transformation from medium to low voltage | APOS, U.
[20]Electric energy necessary to evaporate diethyl ether and toluene, calculated using the vaporization enthalpy for each substance, which are respectively 27 kJ/mol and 38 kJ/mol. The Ecoinvent process used was: Electricity, low voltage {IT}| electricity voltage transformation from medium to low voltage | APOS, U.
[21]Electric energy necessary to the use of the aspiration system for the whole synthesis time, i.e. 9 h. The power was calculated by considering the air flow rate of 250 m^3/h, a total load loss of 110.8076 kg/m^2, and an efficiency of 90%. The Ecoinvent process used was: Electricity, low voltage {IT}| electricity voltage transformation from medium to low voltage | APOS, U.
[22]Avoided Products, it is assumed that 95% of Diethyl ether can be recovered. The Ecoinvent process used was: Diethyl ether, without water, in 99.95% solution state {RoW}| ethylene hydration | APOS, U.
[23]Avoided Products, it is assumed that 80% of Toluene can be recovered. The Ecoinvent process used was: Toluene, liquid {RER}| production | APOS, U.
[24]Amount of toluene released into the atmosphere, as calculated by the formula reported in equation 1 of the main manuscript.
[25]Amount of Diethyl ether released into the atmosphere, as calculated by the formula reported in equation 1 of the main manuscript.
[26]Amount of Sulfuric Acid released into the atmosphere, as calculated by the formula reported in equation 1 of the main manuscript.
[27]Amount of Water released into the atmosphere, as calculated by the formula reported in equation 1 of the main manuscript.
[28]End of life treatment for what used during the synthesis, work up procedure and isolation of the final product. The Ecoinvent process used was: Spent solvent mixture {Europe without Switzerland}| treatment of spent solvent mixture, hazardous waste incineration, with energy recovery | APOS, U.

[29]End of life treatment for the Silicon oil. The Ecoinvent process used was: Spent solvent mixture {Europe without Switzerland}| treatment of spent solvent mixture, hazardous waste incineration, with energy recovery | APOS, U.

Table S35. Contributions for the Life Cycle Inventory (LCI) for the production of 0.80 g of diester derivative **3e** according to the procedure detailed in [11].

Description			Amount	Process Data Source
Input	Materials			
		hydroxyalkyl esters 2e	0.54 g	Modelled from Ecoinvent v 3.8 database sub process as detailed in Table S30
		Levulinic Acid	1.04 g	Modelled from Ecoinvent v 3.8 database sub process as detailed in Table S20
		Toluene	17.4 g	Ecoinvent v 3.8[1]
		H_2SO_4	0.092g	Ecoinvent v 3.8[2]
		K_2CO_3	10 g	Ecoinvent v 3.8[3]
		H_2O	90 g	Ecoinvent v 3.8[4]
		Diethyl ether	35.5 g	Ecoinvent v 3.8[5]
		H_2O	150 g	Ecoinvent v 3.8[6]
		NaCl	50 g	Ecoinvent v 3.8[7]
		Na_2SO_4	10 g	Ecoinvent v 3.8[8]
		Silicon Oil	$1.02*10^{-3}$ kg	Ecoinvent v 3.8[9]
	Equipment/plants	Two neck round bottom flask	$2.43*10^{-4}$ p	Modelled from Ecoinvent v 3.8 database sub process as detailed in Table S21[10]
		Silicon oil container	$1.22*10^{-4}$ p	Modelled from Ecoinvent v 3.8 database sub process as detailed in Table S13[11]
		Magnetic stirrer	$1.4*10^{-4}$ p	Modelled from Ecoinvent v 3.8 database sub process as detailed in Table S15[12]
		Magnetic stirr bar	$7*10^{-5}$ p	Modelled from Ecoinvent v 3.8 database sub process as detailed in Table S14[13]
		Aspiration system	$1*10^{-4}$ p	Modelled from Ecoinvent v 3.8 database sub process as detailed in Table S8[14]
		Activated carbon air filter	$4*10^{-4}$ p	Modelled from Ecoinvent v 3.8 database sub process as detailed in Table S9[15]
	Transports	Transport of raw materials	36.40 kgkm	Ecoinvent v3.8[16]
		Transport of small equipment	0.154 kgkm	Ecoinvent v 3.8[17]

		Transport of large equipment	$1.67*10^{-2}$ tkm	Ecoinvent v 3.8[18]
	Energy	Electric Energy	3500 Wh	Ecoinvent v 3.8[19]
		Electric Energy	20.11 kJ	Ecoinvent v 3.8[20]
		Electric Energy	0.67 kW	Ecoinvent v 3.8[21]
Output	Avoided Products	Diethyl ether	33.64 g	Ecoinvent v 3.8[22]
		Toluene	13.92 g	Ecoinvent v 3.8[23]
	Emission to air	Toluene	$2.82*10^{-5}$ g	SimaPro airborne emission substance list[24]
		Diethyl ether	$8.78*10^{-4}$ g	SimaPro airborne emission substance list[25]
		Sulfuric Acid	$2.64*10^{-12}$ g	SimaPro airborne emission substance list[26]
		Water	$5.53*10^{-5}$ g	SimaPro airborne emission substance list[27]
	End of Life	Spent Solvent Mixture	316.04 g	Ecoinvent v 3.8[28]
		Spent Solvent Mixture	0.0010 kg	Ecoinvent v 3.8[29]

[1]The Ecoinvent process used was: Toluene, liquid {RER}| production | APOS, U.

2Drop of Sulfuric Acid (96%) used. The Ecoinvent process used was: Sulfuric acid {RER}| production | APOS, U.

[3]Sodium carbonate was used during the work up procedure but this material wasn't available on the Ecoinvent database, so Potassium Carbonate was chosen instead. The Ecoinvent process used was: Potassium carbonate {GLO}| production, from potassium hydroxide | APOS, U.

[4]Water necessary for the preparation of the solution of diethyl ether. The Ecoinvent process used was: Water, deionised {Europe without Switzerland} | water production, deionised | APOS, U.

[5]Diethyl ether used during the workup procedure for the extraction of the organic phase. The Ecoinvent process used was: Diethyl ether, without water, in 99.95% solution state {RoW}| ethylene hydration | APOS, U.

[6]Water used for the washing of the organic phase, after the extraction with diethyl ether, in order to remove the residual catalyst. The Ecoinvent process used was: Water, deionised {Europe without Switzerland} | water production, deionised | APOS, U.

[7]Sodium chloride used to wash the organic phase to remove unreacted reagents. The Ecoinvent process used was: Sodium chloride, brine solution {RER}| production | APOS, U.

[8]Sodium sulphate anhydrous used to remove the residual water in the organic phase. The Ecoinvent process used was: Sodium sulphate, anhydrite {RER}| Mannheim process | APOS, U.

[9]The Ecoinvent process used was: Silicone product {RER}| production | APOS, U

[10]The reported value was calculated by the following formula: 1/28800h*7h, where 28800 h is the lifetime of the two neck round bottomed flask and 7h is its time of usage.

[11]The reported value was calculated by the following formula: 1/57600h*7h, where 57600 h is the lifetime of the silicone oil container and 7h is its time of usage.

[12]The reported value was calculated by the following formula: 1/50000h*7h, where 50000 h is the lifetime of the magnetic stirrer and 7h is the time of usage of the magnetic stirrer.

[13]The reported value was calculated by the following formula: 1/100000h*7h, where 100000 h is the lifetime of the stir bar and 7h is its time of usage.

[14]The reported value was calculated by the following formula: 1/79750h*8h, where 79750 h is the lifetime of the aspiration system and 8h is its time of usage. One additional hour of functioning was considered for the work-up procedure.

[15]The reported value was calculated by the following formula: 1/20000h*8h, where 20000 h is the lifetime of the activated carbon air filter and 8h is its time of usage. One additional hour of functioning was considered for the work-up procedure.

[16]Transport of raw materials (reagents). The Ecoinvent process used was: Transport, freight, lorry 3.5-7.5 metric ton, EURO6 {RER}| transport, freight, lorry 3.5-7.5 metric ton, EURO6 | APOS, U. An average distance of 100 km was considered.
[17]Transport of small equipment. The Ecoinvent process used was: Transport, freight, lorry 3.5-7.5 metric ton, EURO6 {RER}| transport, freight, lorry 3.5-7.5 metric ton, EURO6 | APOS, U. an average distance of 100 km was considered.
[18]Transport of large equipment (aspiration system and activated carbon air filter). The Ecoinvent process used was: Transport, freight, lorry 16-32 metric ton, euro6 {RER}| market for transport, freight, lorry 16-32 metric ton, EURO6 | APOS, U. an average distance of 100 km was considered.
[19]Electric energy necessary to heat the reaction mixture at 140°C. It was calculated using power*time, considering a power of 500W for the heating/magnetic stirrer and a reaction time of seven hours. The Ecoinvent process used was: Electricity, low voltage {IT}| electricity voltage transformation from medium to low voltage | APOS, U.
[20]Electric energy necessary to evaporate diethyl ether and toluene, calculated using the vaporization enthalpy for each substance, which are respectively 27 kJ/mol and 38 kJ/mol. The Ecoinvent process used was: Electricity, low voltage {IT}| electricity voltage transformation from medium to low voltage | APOS, U.
[21]Electric energy necessary to the use of the aspiration system for the whole synthesis time, i.e. 9 h. The power was calculated by considering the air flow rate of 250 m^3/h, a total load loss of 110.8076 kg/m^2, and an efficiency of 90%. The Ecoinvent process used was: Electricity, low voltage {IT}| electricity voltage transformation from medium to low voltage | APOS, U.
[22]Avoided Products, it is assumed that 95% of Diethyl ether can be recovered. The Ecoinvent process used was: Diethyl ether, without water, in 99.95% solution state {RoW}| ethylene hydration | APOS, U.
[23]Avoided Products, it is assumed that 80% of Toluene can be recovered. The Ecoinvent process used was: Toluene, liquid {RER}| production | APOS, U.
[24]Amount of toluene released into the atmosphere, as calculated by the formula reported in equation 1 of the main manuscript.
[25]Amount of Diethyl ether released into the atmosphere, as calculated by the formula reported in equation 1 of the main manuscript.
[26]Amount of Sulfuric Acid released into the atmosphere, as calculated by the formula reported in equation 1 of the main manuscript.
[27]Amount of Water released into the atmosphere, as calculated by the formula reported in equation 1 of the main manuscript.
[28]End of life treatment for what used during the synthesis, work up procedure and isolation of the final product. The Ecoinvent process used was: Spent solvent mixture {Europe without Switzerland}| treatment of spent solvent mixture, hazardous waste incineration, with energy recovery | APOS, U.
[29]End of life treatment for the Silicon oil. The Ecoinvent process used was: Spent solvent mixture {Europe without Switzerland}| treatment of spent solvent mixture, hazardous waste incineration, with energy recovery | APOS, U.

Table S36. Contributions for the Life Cycle Inventory (LCI) for the production of 0.24 g of the plasticizer **4a** according to the procedure detailed in [11].

Description			Amount	Process Data Source
Input	Materials			
		Diester derivative 3a	0.37 g	Modelled from Ecoinvent v 3.8 database sub process as detailed in Table S31
		Propylene glycol	0.23 g	Ecoinvent v 3.8[1]
		Toluene	8.7 g	Ecoinvent v 3.8[2]
		P-toluenesulfonic acid	0.019 g	Modelled from Ecoinvent v 3.8 database sub process as detailed in Table S25
		K_2CO_3	10 g	Ecoinvent v 3.8[3]
		H_2O	90 g	Ecoinvent v 3.8[4]
		Diethyl ether	35.5 g	Ecoinvent v 3.8[5]
		H_2O	150 g	Ecoinvent v 3.8[6]

		NaCl	50 g	Ecoinvent v 3.8[7]
		$NaSO_3$	10 g	Ecoinvent v 3.8[8]
		Activated silica	85 g	Ecoinvent v 3.8[9]
		Hexane	275 g	Ecoinvent v 3.8[10]
		Ethyl acetate	75 g	Ecoinvent v 3.8[11]
		Silicon Oil	$1.02*10^{-3}$ kg	Ecoinvent v 3.8[12]
	Equipment/plants	Round-bottom two neck flask	$2.43*10^{-4}$ p	Modelled from Ecoinvent v 3.8 database sub process as detailed in Table S21[13]
		Magnetic stirrer	$1.4*10^{-4}$ p	Modelled from Ecoinvent v 3.8 database sub process as detailed in Table S15[14]
		Magnetic stirr bar	$7*10^{-5}$ p	Modelled from Ecoinvent v 3.8 database sub process as detailed in Table S14[15]
		Silicone oil container	$1.22*10^{-4}$ p	Modelled from Ecoinvent v 3.8 database sub process as detailed in Table S13[16]
		Aspiration system	$1*10^{-4}$ p	Modelled from Ecoinvent v 3.8 database sub process as detailed in Table S8[17]
		Activated carbon air filter	$4*10^{-4}$ p	Modelled from Ecoinvent v 3.8 database sub process as detailed in Table S9[18]
	Transports	Transport of raw materials	70.44 kgkm	Ecoinvent v3.8[19]
		Transport of small equipment	0.15 kgkm	Ecoinvent v 3.8[20]
		Transport of large equipment/plants	0.0167 tkm	Ecoinvent v 3.8[21]
	Energy	Electric Energy	3500 Wh	Ecoinvent v 3.8[22]
		Electric Energy	131.05 kJ	Ecoinvent v 3.8[23]
		Electric Energy	16.52 kJ	Ecoinvent v 3.8[24]
		Electric Energy	0.67 kWh	Ecoinvent v 3.8[25]
Output	Avoided Products	Diethyl ether	33.64 g	Ecoinvent v 3.8[26]
		Toluene	6.96 g	Ecoinvent v 3.8[27]
		Ethyl acetate	59.96 g	Ecoinvent v 3.8[28]
		Hexane	219.77 g	Ecoinvent v 3.8[29]
	Emission to air	Toluene	$1.41*10^{-5}$ g	SimaPro airborne emission substance lis[30]

	1,3-Propanediol	$7.05*10^{-9}$ g	SimaPro airborne emission substance list[31]
	Diethyl ether	$8.78*10^{-4}$ g	SimaPro airborne emission substance list[32]
	Hexane	$2.35*10^{-3}$ g	SimaPro airborne emission substance list[33]
	Ethyl acetate	$3.59*10^{-4}$ g	SimaPro airborne emission substance list[34]
	Water	$5.53 *10^{-5}$ g	SimaPro airborne emission substance list[35]
End of Life	Spent Solvent Mixture	383.99 g	Ecoinvent v 3.8[36]
	Spent Solvent Mixture	$1.02*10^{-3}$ kg	Ecoinvent v 3.8[37]
	Waste treatment	85 g	Ecoinvent v 3.8[38]

[1]The Ecoinvent process used was: Propylene glycol, liquid {RER}| production | APOS, U.
[2]Toluene, liquid {RER}| production | APOS, U.
[3]Sodium carbonate was used during the work up procedure but this material wasn't available on the Ecoinvent database, so Potassium Carbonate was used. The Ecoinvent process used was: Potassium carbonate {GLO}| production, from potassium hydroxide | APOS, U.
[4]Water necessary for the preparation of the solution of diethyl ether. The Ecoinvent process used was: Water, deionised {Europe without Switzerland} | water production, deionised | APOS, U.
[5]Diethyl ether used during thw workup procedure for the extraction of the organic phase. The Ecoinvent process used was: Diethyl ether, without water, in 99.95% solution state {RoW}| ethylene hydration | APOS, U.
[6]Water used for the washing of the organic phase, after the extraction with diethyl ether, in order to remove the residual catalyst. The Ecoinvent process used was: Water, deionised {Europe without Switzerland} | water production, deionised | APOS, U.
[7]Sodium chloride used to wash the organic phase to remove unreacted reagents. The Ecoinvent process used was: Sodium chloride, brine solution {RER}| production | APOS, U.
[8]Sodium sulphate anhydrous used to remove the residual water in the organic phase. The Ecoinvent process used was: Sodium sulphate, anhydrite {RER}| Mannheim process | APOS, U.
[9]Silica used for the column chromatography to purify the residue. The Ecoinvent process used was: Activated silica {GLO}| production | APOS, U.
[10]Hexane used as liquid phase during the column chromatography together with ethyl acetate (n-hexane/EtOAc 5:1). The Ecoinvent process used was: Hexane {RER}| molecular sieve separation of naphtha | APOS, U.
[11]Ethyl acetate used as liquid phase during the column chromatography with hexane (n-hexane/EtOAc 5:1). The Ecoinvent process used was: Ethyl acetate {RER}| production | APOS, U.
[12]The Ecoinvent process used was: Silicone product {RER}| production | APOS, U.
[13]The reported value was calculated by the following formula: 1/28800h*7h, where 28800 h is the lifetime of the two neck round bottomed flask and 7h is its time of usage.
[14]The reported value was calculated by the following formula: 1/50000h*7h, where 50000 h is the lifetime of the magnetic stirrer and 7h is the time of usage of the magnetic stirrer.
[15]The reported value was calculated by the following formula: 1/100000h*7h, where 100000 h is the lifetime of the stir bar and 7h is its time of usage.
[16]The reported value was calculated by the following formula: 1/57600h*7h, where 57600 h is the lifetime of the silicone oil container and 7h is its time of usage.
[17]The reported value was calculated by the following formula: 1/79750h*8h, where 79750 h is the lifetime of the aspiration system and 8h is its time of usage. One additional hour of functioning was considered for the work-up procedure.
[18]The reported value was calculated by the following formula: 1/20000h*8h, where 20000 h is the lifetime of the activated carbon air filter and 8h is its time of usage. One additional hour of functioning was considered for the work-up procedure.

[19]Transport of raw materials. The Ecoinvent process used was: Transport, freight, lorry 3.5-7.5 metric ton, EURO6 {RER}| transport, freight, lorry 3.5-7.5 metric ton, EURO6 | APOS, U. An average distance of 100 km was considered.
[20]Transport of small equipment. The Ecoinvent process used was: Transport, freight, lorry 3.5-7.5 metric ton, EURO6 {RER}| transport, freight, lorry 3.5-7.5 metric ton, EURO6 | APOS, U. an average distance of 100 km was considered.
[21]Transport for large equipment. The Ecoinvent process used was: Transport, freight, lorry 16-32 metric ton, euro6 {RER}| market for transport, freight, lorry 16-32 metric ton, EURO6 | APOS, U. an average distance of 100 km was considered.
[22]Electric energy necessary to heat the reaction mixture at 140°C. It was calculated as power*time, considering a power of 500W and a reaction time of seven hours. The Ecoinvent process used was: Electricity, low voltage {IT}| electricity voltage transformation from medium to low voltage | APOS, U.
[23]Electric energy necessary to evaporate n-hexane and ethyl acetate, calculated using the vaporization enthalpy for each substance, which are respectively 32 kJ/mol and 34 kJ/mol. The Ecoinvent process used was: Electricity, low voltage {IT}| electricity voltage transformation from medium to low voltage | APOS, U.
[24]Electric energy necessary to evaporate diethyl ether and toluene, calculated using the vaporization enthalpy for each substance which are respectively 27 kJ/mol and 38 kJ/mol. The Ecoinvent process used was: Electricity, low voltage {IT}| electricity voltage transformation from medium to low voltage | APOS, U.
[25]Electric energy necessary to the use of the aspiration system for the whole synthesis time, i.e. 8 h. The power was calculated by considering the air flow rate of 250 m^3/h, a total load loss of 110.8076 kg/m^2, and an efficiency of 90%. The Ecoinvent process used was: Electricity, low voltage {IT}| electricity voltage transformation from medium to low voltage | APOS, U.
[26]Avoided Products, it is assumed that 95% of Diethyl ether can be recovered. The Ecoinvent process used was: Diethyl ether, without water, in 99.95% solution state {RoW}| ethylene hydration | APOS, U.
[27]Avoided Products, it is assumed that 80% of Toluene can be recovered. The Ecoinvent process used was: Toluene, liquid {RER}| production | APOS, U.
[28]Avoided Products, it is assumed that 80% of Ethyl Acetate can be recovered. The Ecoinvent process used was: Ethyl acetate {RER}| production | APOS, U.
[29]Avoided Products, it is assumed that 80% of Hexane can be recovered. The Ecoinvet process used was: Hexane {RER}| molecular sieve separation of naphtha | APOS, U.
[30]Amount of toluene released into the atmosphere, as calculated by the formula reported in equation 1 of the main manuscript.
[31]Amount of 1,3-Propanediol released into the atmosphere, as calculated by the formula reported in equation 1 of the main manuscript.
[32]Amount of Diethyl ether released into the atmosphere, as calculated by the formula reported in equation 1 of the main manuscript.
[33]Amount of Hexane released into the atmosphere, as calculated by the formula reported in equation 1 of the main manuscript.
[34]Amount of Ethyl Acetate released into the atmosphere, as calculated by the formula reported in equation 1 of the main manuscript.
[35]Amount of Water released into the atmosphere, as calculated by the formula reported in equation 1 of the main manuscript.
[36]End of life treatment for what used during the synthesis, work up procedure and isolation of the final product. The Ecoinvent process used was: Spent solvent mixture {Europe without Switzerland}| treatment of spent solvent mixture, hazardous waste incineration, with recovery energy | APOS, U.
[37]End of life treatment for the silicon oil The Ecoinvent process used was: Spent solvent mixture {Europe without Switzerland}| treatment of spent solvent mixture, hazardous waste incineration, with recovery energy | APOS, U.
[38]End of life chosen for the Activated Silica used for the column chromatography, which is not recoverable. The Ecoinvent process used was: Hazardous waste, for incineration {Europe without Switzerland}| treatment of hazardous waste, hazardous waste incineration, with energy recovery | APOS, U.

Table S37. Contributions for the Life Cycle Inventory (LCI) for the production of 0.29 g of the plasticizer **4b** according to the procedure detailed in [11].

		Description	Amount	Process Data Source
Input	Materials			
		Diester derivative 3b	0.33 g	Modelled from Ecoinvent v 3.8 database sub process as detailed in Table S32
		Propylene glycol	0.23 g	Ecoinvent v 3.8[1]

	Toluene	8.7 g	Ecoinvent v 3.8[2]
	p-toluenesulfonic acid	0.019 g	Modelled from Ecoinvent v 3.8 database sub process as detailed in Table S25
	K_2CO_3	10 g	Ecoinvent v 3.8[3]
	H_2O	90 g	Ecoinvent v 3.8[4]
	Diethyl ether	35.5 g	Ecoinvent v 3.8[5]
	H_2O	150 g	Ecoinvent v 3.8[6]
	NaCl	50 g	Ecoinvent v 3.8[7]
	$NaSO_3$	10 g	Ecoinvent v 3.8[8]
	Activated silica	85 g	Ecoinvent v 3.8[9]
	Hexane	275 g	Ecoinvent v 3.8[10]
	Ethyl acetate	75 g	Ecoinvent v 3.8[11]
	Silicon Oil	$1.02*10^{-3}$ kg	Ecoinvent v 3.8[12]
Equipment/plants	Round-bottom two neck flask	$2.43*10^{-4}$ p	Modelled from Ecoinvent v 3.8 database sub process as detailed in Table S21[13]
	Magnetic stirrer	$1.4*10^{-4}$ p	Modelled from Ecoinvent v 3.8 database sub process as detailed in Table S15[14]
	Magnetic stirr bar	$7*10^{-5}$ p	Modelled from Ecoinvent v 3.8 database sub process as detailed in Table S14[15]
	Silicone oil container	$1.22*10^{-4}$ p	Modelled from Ecoinvent v 3.8 database sub process as detailed in Table S13[16]
	Aspiration system	$1*10^{-4}$ p	Modelled from Ecoinvent v 3.8 database sub process as detailed in Table S8[17]
	Activated carbon air filter	$4*10^{-4}$ p	Modelled from Ecoinvent v 3.8 database sub process as detailed in Table S9[18]
Transports	Transport of raw materials	70.44 kgkm	Ecoinvent v3.8[19]
	Transport of small equipment	0.15 kgkm	Ecoinvent v 3.8[20]
	Transport of large equipment/plants	0.0167 tkm	Ecoinvent v 3.8[21]
Energy	Electric Energy	3500 Wh	Ecoinvent v 3.8[22]
	Electric Energy	131.05 kJ	Ecoinvent v 3.8[23]
	Electric Energy	16.52 kJ	Ecoinvent v 3.8[24]
	Electric Energy	0.67 kWh	Ecoinvent v 3.8[25]

Output	Avoided Products	Diethyl ether	33.64 g	Ecoinvent v 3.8[26]
		Toluene	6.96 g	Ecoinvent v 3.8[27]
		Ethyl acetate	59.96 g	Ecoinvent v 3.8[28]
		Hexane	219.77 g	Ecoinvent v 3.8[29]
	Emission to air	Toluene	$1.41*10^{-5}$ g	SimaPro airborne emission substance lis[30]
		1,3-Propanediol	$7.05*10^{-9}$ g	SimaPro airborne emission substance list[31]
		Diethyl ether	$8.78*10^{-4}$ g	SimaPro airborne emission substance list[32]
		Hexane	$2.35*10^{-3}$ g	SimaPro airborne emission substance list[33]
		Ethyl acetate	$3.59*10^{-4}$ g	SimaPro airborne emission substance list[34]
		Water	$5.53 *10^{-5}$ g	SimaPro airborne emission substance list[35]
	End of Life	Spent Solvent Mixture	384.27 g	Ecoinvent v 3.8[36]
		Spent Solvent Mixture	$1.02*10^{-3}$ kg	Ecoinvent v 3.8[37]
		Waste treatment	85 g	Ecoinvent v 3.8[38]

[1]The Ecoinvent process used was: Propylene glycol, liquid {RER}| production | APOS, U.
[2]Toluene, liquid {RER}| production | APOS, U.
[3]Sodium carbonate was used during the work up procedure but this material wasn't available on the Ecoinvent database, so Potassium Carbonate was used. The Ecoinvent process used was: Potassium carbonate {GLO}| production, from potassium hydroxide | APOS, U.
[4]Water necessary for the preparation of the solution of diethyl ether. The Ecoinvent process used was: Water, deionised {Europe without Switzerland} | water production, deionised | APOS, U.
[5]Diethyl ether used during thw workup procedure for the extraction of the organic phase. The Ecoinvent process used was: Diethyl ether, without water, in 99.95% solution state {RoW}| ethylene hydration | APOS, U.
[6]Water used for the washing of the organic phase, after the extraction with diethyl ether, in order to remove the residual catalyst. The Ecoinvent process used was: Water, deionised {Europe without Switzerland} | water production, deionised | APOS, U.
[7]Sodium chloride used to wash the organic phase to remove unreacted reagents. The Ecoinvent process used was: Sodium chloride, brine solution {RER}| production | APOS, U.
[8]Sodium sulphate anhydrous used to remove the residual water in the organic phase. The Ecoinvent process used was: Sodium sulphate, anhydrite {RER}| Mannheim process | APOS, U.
[9]Silica used for the column chromatography to purify the residue. The Ecoinvent process used was: Activated silica {GLO}| production | APOS, U.
[10]Hexane used as liquid phase during the column chromatography together with ethyl acetate (n-hexane/EtOAc 5:1). The Ecoinvent process used was: Hexane {RER}| molecular sieve separation of naphtha | APOS, U.
[11]Ethyl acetate used as liquid phase during the column chromatography with hexane (n-hexane/EtOAc 5:1). The Ecoinvent process used was: Ethyl acetate {RER}| production | APOS, U.
[12]The Ecoinvent process used was: Silicone product {RER}| production | APOS, U.

[13]The reported value was calculated by the following formula: 1/28800h*7h, where 28800 h is the lifetime of the two neck round bottomed flask and 7h is its time of usage.
[14]The reported value was calculated by the following formula: 1/50000h*7h, where 50000 h is the lifetime of the magnetic stirrer and 7h is the time of usage of the magnetic stirrer.
[15]The reported value was calculated by the following formula: 1/100000h*7h, where 100000 h is the lifetime of the stir bar and 7h is its time of usage.
[16]The reported value was calculated by the following formula: 1/57600h*7h, where 57600 h is the lifetime of the silicone oil container and 7h is its time of usage.
[17]The reported value was calculated by the following formula: 1/79750h*8h, where 79750 h is the lifetime of the aspiration system and 8h is its time of usage. One additional hour of functioning was considered for the work-up procedure.
[18]The reported value was calculated by the following formula: 1/20000h*8h, where 20000 h is the lifetime of the activated carbon air filter and 8h is its time of usage. One additional hour of functioning was considered for the work-up procedure.
[19]Transport of raw materials. The Ecoinvent process used was: Transport, freight, lorry 3.5-7.5 metric ton, EURO6 {RER}| transport, freight, lorry 3.5-7.5 metric ton, EURO6 | APOS, U. An average distance of 100 km was considered.
[20]Transport of small equipment. The Ecoinvent process used was: Transport, freight, lorry 3.5-7.5 metric ton, EURO6 {RER}| transport, freight, lorry 3.5-7.5 metric ton, EURO6 | APOS, U. an average distance of 100 km was considered.
[21]Transport for large equipment. The Ecoinvent process used was: Transport, freight, lorry 16-32 metric ton, euro6 {RER}| market for transport, freight, lorry 16-32 metric ton, EURO6 | APOS, U. an average distance of 100 km was considered.
[22]Electric energy necessary to heat the reaction mixture at 140°C. It was calculated as power*time, considering a power of 500W and a reaction time of seven hours. The Ecoinvent process used was: Electricity, low voltage {IT}| electricity voltage transformation from medium to low voltage | APOS, U.
[23]Electric energy necessary to evaporate n-hexane and ethyl acetate, calculated using the vaporization enthalpy for each substance, which are respectively 32 kJ/mol and 34 kJ/mol. The Ecoinvent process used was: Electricity, low voltage {IT}| electricity voltage transformation from medium to low voltage | APOS, U.
[24]Electric energy necessary to evaporate diethyl ether and toluene, calculated using the vaporization enthalpy for each substance which are respectively 27 kJ/mol and 38 kJ/mol. The Ecoinvent process used was: Electricity, low voltage {IT}| electricity voltage transformation from medium to low voltage | APOS, U.
[25]Electric energy necessary to the use of the aspiration system for the whole synthesis time, i.e. 8 h. The power was calculated by considering the air flow rate of 250 m^3/h, a total load loss of 110.8076 kg/m^2, and an efficiency of 90%. The Ecoinvent process used was: Electricity, low voltage {IT}| electricity voltage transformation from medium to low voltage | APOS, U.
[26]Avoided Products, it is assumed that 95% of Diethyl ether can be recovered. The Ecoinvent process used was: Diethyl ether, without water, in 99.95% solution state {RoW}| ethylene hydration | APOS, U.
[27]Avoided Products, it is assumed that 80% of Toluene can be recovered. The Ecoinvent process used was: Toluene, liquid {RER}| production | APOS, U.
[28]Avoided Products, it is assumed that 80% of Ethyl Acetate can be recovered. The Ecoinvent process used was: Ethyl acetate {RER}| production | APOS, U.
[29]Avoided Products, it is assumed that 80% of Hexane can be recovered. The Ecoinvent process used was: Hexane {RER}| molecular sieve separation of naphtha | APOS, U.
[30]Amount of toluene released into the atmosphere, as calculated by the formula reported in equation 1 of the main manuscript.
[31]Amount of 1,3-Propanediol released into the atmosphere, as calculated by the formula reported in equation 1 of the main manuscript.
[32]Amount of Diethyl ether released into the atmosphere, as calculated by the formula reported in equation 1 of the main manuscript.
[33]Amount of Hexane released into the atmosphere, as calculated by the formula reported in equation 1 of the main manuscript.
[34]Amount of Ethyl Acetate released into the atmosphere, as calculated by the formula reported in equation 1 of the main manuscript.
[35]Amount of Water released into the atmosphere, as calculated by the formula reported in equation 1 of the main manuscript.
[36]End of life treatment for what used during the synthesis, work up procedure and isolation of the final product. The Ecoinvent process used was: Spent solvent mixture {Europe without Switzerland}| treatment of spent solvent mixture, hazardous waste incineration, with recovery energy | APOS, U.
[37]End of life treatment for the silicon oil The Ecoinvent process used was: Spent solvent mixture {Europe without Switzerland}| treatment of spent solvent mixture, hazardous waste incineration, with recovery energy | APOS, U.
[38]End of life chosen for the Activated Silica used for the column chromatography, which is not recoverable. The Ecoinvent process used was: Hazardous waste, for incineration {Europe without Switzerland}| treatment of hazardous waste, hazardous waste incineration, with energy recovery | APOS, U.

Table S38. Contributions for the Life Cycle Inventory (LCI) for the production of 0.151 g of the plasticizer **4c** according to the procedure detailed in [11].

Description			Amount	Process Data Source
Input	Materials			
		Diester derivative 3c	0.24 g	Modelled from Ecoinvent v 3.8 database sub process as detailed in Table S33
		Propylene glycol	0.23 g	Ecoinvent v 3.8[1]
		Toluene	8.7 g	Ecoinvent v 3.8[2]
		P-toluenesulfonic acid	0.019 g	Modelled from Ecoinvent v 3.8 database sub process as detailed in Table S25
		K_2CO_3	10 g	Ecoinvent v 3.8[3]
		H_2O	90 g	Ecoinvent v 3.8[4]
		Diethyl ether	35.5 g	Ecoinvent v 3.8[5]
		H_2O	150 g	Ecoinvent v 3.8[6]
		NaCl	50 g	Ecoinvent v 3.8[7]
		$NaSO_3$	10 g	Ecoinvent v 3.8[8]
		Activated silica	85 g	Ecoinvent v 3.8[9]
		Hexane	275 g	Ecoinvent v 3.8[10]
		Ethyl acetate	75 g	Ecoinvent v 3.8[11]
		Silicon Oil	$1.02*10^{-3}$ kg	Ecoinvent v 3.8[12]
	Equipment/plants	Round-bottom two neck flask	$2.43*10^{-4}$ p	Modelled from Ecoinvent v 3.8 database sub process as detailed in Table S21[13]
		Magnetic stirrer	$1.4*10^{-4}$ p	Modelled from Ecoinvent v 3.8 database sub process as detailed in Table S15[14]
		Magnetic stirr bar	$7*10^{-5}$ p	Modelled from Ecoinvent v 3.8 database sub process as detailed in Table S14[15]
		Silicone oil container	$1.22*10^{-4}$ p	Modelled from Ecoinvent v 3.8 database sub process as detailed in Table S13[16]
		Aspiration system	$1*10^{-4}$ p	Modelled from Ecoinvent v 3.8 database sub process as detailed in Table S8[17]
		Activated carbon air filter	$4*10^{-4}$ p	Modelled from Ecoinvent v 3.8 database sub process as detailed in Table S9[18]
	Transports	Transport of raw materials	70.44 kgkm	Ecoinvent v3.8[19]

		Transport of small equipment	0.15 kgkm	Ecoinvent v 3.8[20]
		Transport of large equipment/plants	0.0167 tkm	Ecoinvent v 3.8[21]
	Energy	Electric Energy	3500 Wh	Ecoinvent v 3.8[22]
		Electric Energy	131.05 kJ	Ecoinvent v 3.8[23]
		Electric Energy	16.52 kJ	Ecoinvent v 3.8[24]
		Electric Energy	0.67 kWh	Ecoinvent v 3.8[25]
Output	Avoided Products	Diethyl ether	33.64 g	Ecoinvent v 3.8[26]
		Toluene	6.96 g	Ecoinvent v 3.8[27]
		Ethyl acetate	59.96 g	Ecoinvent v 3.8[28]
		Hexane	219.77 g	Ecoinvent v 3.8[29]
	Emission to air	Toluene	$1.41*10^{-5}$ g	SimaPro airborne emission substance lis[30]
		1,3-Propanediol	$7.05*10^{-9}$ g	SimaPro airborne emission substance list[31]
		Diethyl ether	$8.78*10^{-4}$ g	SimaPro airborne emission substance list[32]
		Hexane	$2.35*10^{-3}$ g	SimaPro airborne emission substance list[33]
		Ethyl acetate	$3.59*10^{-4}$ g	SimaPro airborne emission substance list[34]
		Water	$5.53 *10^{-5}$ g	SimaPro airborne emission substance list[35]
	End of Life	Spent Solvent Mixture	383.94 g	Ecoinvent v 3.8[36]
		Spent Solvent Mixture	$1.02*10^{-3}$ kg	Ecoinvent v 3.8[37]
		Waste treatment	85 g	Ecoinvent v 3.8[38]

[1]The Ecoinvent process used was: Propylene glycol, liquid {RER}| production | APOS, U.
[2]Toluene, liquid {RER}| production | APOS, U.
[3]Sodium carbonate was used during the work up procedure but this material wasn't available on the Ecoinvent database, so Potassium Carbonate was used. The Ecoinvent process used was: Potassium carbonate {GLO}| production, from potassium hydroxide | APOS, U.
[4]Water necessary for the preparation of the solution of diethyl ether. The Ecoinvent process used was: Water, deionised {Europe without Switzerland} | water production, deionised | APOS, U.
[5]Diethyl ether used during thw workup procedure for the extraction of the organic phase. The Ecoinvent process used was: Diethyl ether, without water, in 99.95% solution state {RoW}| ethylene hydration | APOS, U.

[6]Water used for the washing of the organic phase, after the extraction with diethyl ether, in order to remove the residual catalyst. The Ecoinvent process used was: Water, deionised {Europe without Switzerland} | water production, deionised | APOS, U.
[7]Sodium chloride used to wash the organic phase to remove unreacted reagents. The Ecoinvent process used was: Sodium chloride, brine solution {RER}| production | APOS, U.
[8]Sodium sulphate anhydrous used to remove the residual water in the organic phase. The Ecoinvent process used was: Sodium sulphate, anhydrite {RER}| Mannheim process | APOS, U.
[9]Silica used for the column chromatography to purify the residue. The Ecoinvent process used was: Activated silica {GLO}| production | APOS, U.
[10]Hexane used as liquid phase during the column chromatography together with ethyl acetate (n-hexane/EtOAc 5:1). The Ecoinvent process used was: Hexane {RER}| molecular sieve separation of naphtha | APOS, U.
[11]Ethyl acetate used as liquid phase during the column chromatography with hexane (n-hexane/EtOAc 5:1). The Ecoinvent process used was: Ethyl acetate {RER}| production | APOS, U.
[12]The Ecoinvent process used was: Silicone product {RER}| production | APOS, U.
[13]The reported value was calculated by the following formula: 1/28800h*7h, where 28800 h is the lifetime of the two neck round bottomed flask and 7h is its time of usage.
[14]The reported value was calculated by the following formula: 1/50000h*7h, where 50000 h is the lifetime of the magnetic stirrer and 7h is the time of usage of the magnetic stirrer.
[15]The reported value was calculated by the following formula: 1/100000h*7h, where 100000 h is the lifetime of the stir bar and 7h is its time of usage.
[16]The reported value was calculated by the following formula: 1/57600h*7h, where 57600 h is the lifetime of the silicone oil container and 7h is its time of usage.
[17]The reported value was calculated by the following formula: 1/79750h*8h, where 79750 h is the lifetime of the aspiration system and 8h is its time of usage. One additional hour of functioning was considered for the work-up procedure.
[18]The reported value was calculated by the following formula: 1/20000h*8h, where 20000 h is the lifetime of the activated carbon air filter and 8h is its time of usage. One additional hour of functioning was considered for the work-up procedure.
[19]Transport of raw materials. The Ecoinvent process used was: Transport, freight, lorry 3.5-7.5 metric ton, EURO6 {RER}| transport, freight, lorry 3.5-7.5 metric ton, EURO6 | APOS, U. An average distance of 100 km was considered.
[20]Transport of small equipment. The Ecoinvent process used was: Transport, freight, lorry 3.5-7.5 metric ton, EURO6 {RER}| transport, freight, lorry 3.5-7.5 metric ton, EURO6 | APOS, U. an average distance of 100 km was considered.
[21]Transport for large equipment. The Ecoinvent process used was: Transport, freight, lorry 16-32 metric ton, euro6 {RER}| market for transport, freight, lorry 16-32 metric ton, EURO6 | APOS, U. an average distance of 100 km was considered.
[22]Electric energy necessary to heat the reaction mixture at 140°C. It was calculated as power*time, considering a power of 500W and a reaction time of seven hours. The Ecoinvent process used was: Electricity, low voltage {IT}| electricity voltage transformation from medium to low voltage | APOS, U.
[23]Electric energy necessary to evaporate n-hexane and ethyl acetate, calculated using the vaporization enthalpy for each substance, which are respectively 32 kJ/mol and 34 kJ/mol. The Ecoinvent process used was: Electricity, low voltage {IT}| electricity voltage transformation from medium to low voltage | APOS, U.
[24]Electric energy necessary to evaporate diethyl ether and toluene, calculated using the vaporization enthalpy for each substance which are respectively 27 kJ/mol and 38 kJ/mol. The Ecoinvent process used was: Electricity, low voltage {IT}| electricity voltage transformation from medium to low voltage | APOS, U.
[25]Electric energy necessary to the use of the aspiration system for the whole synthesis time, i.e. 8 h. The power was calculated by considering the air flow rate of 250 m^3/h, a total load loss of 110.8076 kg/m^2, and an efficiency of 90%. The Ecoinvent process used was: Electricity, low voltage {IT}| electricity voltage transformation from medium to low voltage | APOS, U.
[26]Avoided Products, it is assumed that 95% of Diethyl ether can be recovered. The Ecoinvent process used was: Diethyl ether, without water, in 99.95% solution state {RoW}| ethylene hydration | APOS, U.
[27]Avoided Products, it is assumed that 80% of Toluene can be recovered. The Ecoinvent process used was: Toluene, liquid {RER}| production | APOS, U.
[28]Avoided Products, it is assumed that 80% of Ethyl Acetate can be recovered. The Ecoinvent process used was: Ethyl acetate {RER}| production | APOS, U.
[29]Avoided Products, it is assumed that 80% of Hexane can be recovered. The Ecoinvet process used was: Hexane {RER}| molecular sieve separation of naphtha | APOS, U.
[30]Amount of toluene released into the atmosphere, as calculated by the formula reported in equation 1 of the main manuscript.
[31]Amount of 1,3-Propanediol released into the atmosphere, as calculated by the formula reported in equation 1 of the main manuscript.
[32]Amount of Diethyl ether released into the atmosphere, as calculated by the formula reported in equation 1 of the main manuscript.
[33]Amount of Hexane released into the atmosphere, as calculated by the formula reported in equation 1 of the main

manuscript.
[34]Amount of Ethyl Acetate released into the atmosphere, as calculated by the formula reported in equation 1 of the main manuscript.
[35]Amount of Water released into the atmosphere, as calculated by the formula reported in equation 1 of the main manuscript.
[36]End of life treatment for what used during the synthesis, work up procedure and isolation of the final product. The Ecoinvent process used was: Spent solvent mixture {Europe without Switzerland}| treatment of spent solvent mixture, hazardous waste incineration, with recovery energy | APOS, U.
[37]End of life treatment for the silicon oil The Ecoinvent process used was: Spent solvent mixture {Europe without Switzerland}| treatment of spent solvent mixture, hazardous waste incineration, with recovery energy | APOS, U.
[38]End of life chosen for the Activated Silica used for the column chromatography, which is not recoverable. The Ecoinvent process used was: Hazardous waste, for incineration {Europe without Switzerland}| treatment of hazardous waste, hazardous waste incineration, with energy recovery | APOS, U.

Table S39. Contributions for the Life Cycle Inventory (LCI) for the production of 0.161 g of the plasticizer **4d** according to the procedure detailed in [11].

Description			Amount	Process Data Source
Input	Materials			
		Diester derivative 3d	0.26 g	Modelled from Ecoinvent v 3.8 database sub process as detailed in Table S34
		Propylene glycol	0.23 g	Ecoinvent v 3.8[1]
		Toluene	8.7 g	Ecoinvent v 3.8[2]
		p-toluenesulfonic acid	0.019 g	Modelled from Ecoinvent v 3.8 database sub process as detailed in Table S25
		K_2CO_3	10 g	Ecoinvent v 3.8[3]
		H_2O	90 g	Ecoinvent v 3.8[4]
		Diethyl ether	35.5 g	Ecoinvent v 3.8[5]
		H_2O	150 g	Ecoinvent v 3.8[6]
		NaCl	50 g	Ecoinvent v 3.8[7]
		$NaSO_3$	10 g	Ecoinvent v 3.8[8]
		Activated silica	85 g	Ecoinvent v 3.8[9]
		Hexane	275 g	Ecoinvent v 3.8[10]
		Ethyl acetate	75 g	Ecoinvent v 3.8[11]
		Silicon Oil	$1.02*10^{-3}$ kg	Ecoinvent v 3.8[12]
	Equipment/plants	Round-bottom two neck flask	$2.43*10^{-4}$ p	Modelled from Ecoinvent v 3.8 database sub process as detailed in Table S21[13]
		Magnetic stirrer	$1.4*10^{-4}$ p	Modelled from Ecoinvent v 3.8 database sub process as detailed in Table S15[14]
		Magnetic stirr bar	$7*10^{-5}$ p	Modelled from Ecoinvent v 3.8 database sub process as detailed in Table S14[15]

		Silicone oil container	$1.22*10^{-4}$ p	Modelled from Ecoinvent v 3.8 database sub process as detailed in Table S13[16]
		Aspiration system	$1*10^{-4}$ p	Modelled from Ecoinvent v 3.8 database sub process as detailed in Table S8[17]
		Activated carbon air filter	$4*10^{-4}$ p	Modelled from Ecoinvent v 3.8 database sub process as detailed in Table S9[18]
	Transports	Transport of raw materials	70.44 kgkm	Ecoinvent v3.8[19]
		Transport of small equipment	0.15 kgkm	Ecoinvent v 3.8[20]
		Transport of large equipment/plants	0.0167 tkm	Ecoinvent v 3.8[21]
	Energy	Electric Energy	3500 Wh	Ecoinvent v 3.8[22]
		Electric Energy	131.05 kJ	Ecoinvent v 3.8[23]
		Electric Energy	16.52 kJ	Ecoinvent v 3.8[24]
		Electric Energy	0.67 kWh	Ecoinvent v 3.8[25]
Output	Avoided Products	Diethyl ether	33.64 g	Ecoinvent v 3.8[26]
		Toluene	6.96 g	Ecoinvent v 3.8[27]
		Ethyl acetate	59.96 g	Ecoinvent v 3.8[28]
		Hexane	219.77 g	Ecoinvent v 3.8[29]
	Emission to air	Toluene	$1.41*10^{-5}$ g	SimaPro airborne emission substance list[30]
		1,3-Propanediol	$7.05*10^{-9}$ g	SimaPro airborne emission substance list[31]
		Diethyl ether	$8.78*10^{-4}$ g	SimaPro airborne emission substance list[32]
		Hexane	$2.35*10^{-3}$ g	SimaPro airborne emission substance list[33]
		Ethyl acetate	$3.59*10^{-4}$ g	SimaPro airborne emission substance list[34]
		Water	$5.53 *10^{-5}$ g	SimaPro airborne emission substance list[35]
	End of Life	Spent Solvent Mixture	384.01 g	Ecoinvent v 3.8[36]

	Spent Solvent Mixture	$1.02*10^{-3}$ kg	Ecoinvent v 3.8[37]
	Waste treatment	85 g	Ecoinvent v 3.8[38]

[1]The Ecoinvent process used was: Propylene glycol, liquid {RER}| production | APOS, U.
[2]Toluene, liquid {RER}| production | APOS, U.
[3]Sodium carbonate was used during the work up procedure but this material wasn't available on the Ecoinvent database, so Potassium Carbonate was used. The Ecoinvent process used was: Potassium carbonate {GLO}| production, from potassium hydroxide | APOS, U.
[4]Water necessary for the preparation of the solution of diethyl ether. The Ecoinvent process used was: Water, deionised {Europe without Switzerland} | water production, deionised | APOS, U.
[5]Diethyl ether used during thw workup procedure for the extraction of the organic phase. The Ecoinvent process used was: Diethyl ether, without water, in 99.95% solution state {RoW}| ethylene hydration | APOS, U.
[6]Water used for the washing of the organic phase, after the extraction with diethyl ether, in order to remove the residual catalyst. The Ecoinvent process used was: Water, deionised {Europe without Switzerland} | water production, deionised | APOS, U.
[7]Sodium chloride used to wash the organic phase to remove unreacted reagents. The Ecoinvent process used was: Sodium chloride, brine solution {RER}| production | APOS, U.
[8]Sodium sulphate anhydrous used to remove the residual water in the organic phase. The Ecoinvent process used was: Sodium sulphate, anhydrite {RER}| Mannheim process | APOS, U.
[9]Silica used for the column chromatography to purify the residue. The Ecoinvent process used was: Activated silica {GLO}| production | APOS, U.
[10]Hexane used as liquid phase during the column chromatography together with ethyl acetate (n-hexane/EtOAc 5:1). The Ecoinvent process used was: Hexane {RER}| molecular sieve separation of naphtha | APOS, U.
[11]Ethyl acetate used as liquid phase during the column chromatography with hexane (n-hexane/EtOAc 5:1). The Ecoinvent process used was: Ethyl acetate {RER}| production | APOS, U.
[12]The Ecoinvent process used was: Silicone product {RER}| production | APOS, U.
[13]The reported value was calculated by the following formula: 1/28800h*7h, where 28800 h is the lifetime of the two neck round bottomed flask and 7h is its time of usage.
[14]The reported value was calculated by the following formula: 1/50000h*7h, where 50000 h is the lifetime of the magnetic stirrer and 7h is the time of usage of the magnetic stirrer.
[15]The reported value was calculated by the following formula: 1/100000h*7h, where 100000 h is the lifetime of the stir bar and 7h is its time of usage.
[16]The reported value was calculated by the following formula: 1/57600h*7h, where 57600 h is the lifetime of the silicone oil container and 7h is its time of usage.
[17]The reported value was calculated by the following formula: 1/79750h*8h, where 79750 h is the lifetime of the aspiration system and 8h is its time of usage. One additional hour of functioning was considered for the work-up procedure.
[18]The reported value was calculated by the following formula: 1/20000h*8h, where 20000 h is the lifetime of the activated carbon air filter and 8h is its time of usage. One additional hour of functioning was considered for the work-up procedure.
[19]Transport of raw materials. The Ecoinvent process used was: Transport, freight, lorry 3.5-7.5 metric ton, EURO6 {RER}| transport, freight, lorry 3.5-7.5 metric ton, EURO6 | APOS, U. An average distance of 100 km was considered.
[20]Transport of small equipment. The Ecoinvent process used was: Transport, freight, lorry 3.5-7.5 metric ton, EURO6 {RER}| transport, freight, lorry 3.5-7.5 metric ton, EURO6 | APOS, U. an average distance of 100 km was considered.
[21]Transport for large equipment. The Ecoinvent process used was: Transport, freight, lorry 16-32 metric ton, euro6 {RER}| market for transport, freight, lorry 16-32 metric ton, EURO6 | APOS, U. an average distance of 100 km was considered.
[22]Electric energy necessary to heat the reaction mixture at 140°C. It was calculated as power*time, considering a power of 500W and a reaction time of seven hours. The Ecoinvent process used was: Electricity, low voltage {IT}| electricity voltage transformation from medium to low voltage | APOS, U.
[23]Electric energy necessary to evaporate n-hexane and ethyl acetate, calculated using the vaporization enthalpy for each substance, which are respectively 32 kJ/mol and 34 kJ/mol. The Ecoinvent process used was: Electricity, low voltage {IT}| electricity voltage transformation from medium to low voltage | APOS, U.
[24]Electric energy necessary to evaporate diethyl ether and toluene, calculated using the vaporization enthalpy for each substance which are respectively 27 kJ/mol and 38 kJ/mol. The Ecoinvent process used was: Electricity, low voltage {IT}| electricity voltage transformation from medium to low voltage | APOS, U.
[25]Electric energy necessary to the use of the aspiration system for the whole synthesis time, i.e. 8 h. The power was calculated by considering the air flow rate of 250 m^3/h, a total load loss of 110.8076 kg/m^2, and an efficiency of 90%. The Ecoinvent process used was: Electricity, low voltage {IT}| electricity voltage transformation from medium to low voltage | APOS, U.

[26]Avoided Products, it is assumed that 95% of Diethyl ether can be recovered. The Ecoinvent process used was: Diethyl ether, without water, in 99.95% solution state {RoW}| ethylene hydration | APOS, U.
[27]Avoided Products, it is assumed that 80% of Toluene can be recovered. The Ecoinvent process used was: Toluene, liquid {RER}| production | APOS, U.
[28]Avoided Products, it is assumed that 80% of Ethyl Acetate can be recovered. The Ecoinvent process used was: Ethyl acetate {RER}| production | APOS, U.
[29]Avoided Products, it is assumed that 80% of Hexane can be recovered. The Ecoinvet process used was: Hexane {RER}| molecular sieve separation of naphtha | APOS, U.
[30]Amount of toluene released into the atmosphere, as calculated by the formula reported in equation 1 of the main manuscript.
[31]Amount of 1,3-Propanediol released into the atmosphere, as calculated by the formula reported in equation 1 of the main manuscript.
[32]Amount of Diethyl ether released into the atmosphere, as calculated by the formula reported in equation 1 of the main manuscript.
[33]Amount of Hexane released into the atmosphere, as calculated by the formula reported in equation 1 of the main manuscript.
[34]Amount of Ethyl Acetate released into the atmosphere, as calculated by the formula reported in equation 1 of the main manuscript.
[35]Amount of Water released into the atmosphere, as calculated by the formula reported in equation 1 of the main manuscript.
[36]End of life treatment for what used during the synthesis, work up procedure and isolation of the final product. The Ecoinvent process used was: Spent solvent mixture {Europe without Switzerland}| treatment of spent solvent mixture, hazardous waste incineration, with recovery energy | APOS, U.
[37]End of life treatment for the silicon oil The Ecoinvent process used was: Spent solvent mixture {Europe without Switzerland}| treatment of spent solvent mixture, hazardous waste incineration, with recovery energy | APOS, U.
[38]End of life chosen for the Activated Silica used for the column chromatography, which is not recoverable. The Ecoinvent process used was: Hazardous waste, for incineration {Europe without Switzerland}| treatment of hazardous waste, hazardous waste incineration, with energy recovery | APOS, U.

Table S40. Contributions for the Life Cycle Inventory (LCI) for the production of 0.168 g of the plasticizer **4e** according to the procedure detailed in [11].

Description			Amount	Process Data Source
Input	Materials			
		Diester derivative 3e	0.28 g	Modelled from Ecoinvent v 3.8 database sub process as detailed in Table S35
		Propylene glycol	0.23 g	Ecoinvent v 3.8[1]
		Toluene	8.7 g	Ecoinvent v 3.8[2]
		P-toluenesulfonic acid	0.019 g	Modelled from Ecoinvent v 3.8 database sub process as detailed in Table S25
		K_2CO_3	10 g	Ecoinvent v 3.8[3]
		H_2O	90 g	Ecoinvent v 3.8[4]
		Diethyl ether	35.5 g	Ecoinvent v 3.8[5]
		H_2O	150 g	Ecoinvent v 3.8[6]
		NaCl	50 g	Ecoinvent v 3.8[7]
		$NaSO_3$	10 g	Ecoinvent v 3.8[8]
		Activated silica	85 g	Ecoinvent v 3.8[9]
		Hexane	275 g	Ecoinvent v 3.8[10]

		Ethyl acetate	75 g	Ecoinvent v 3.8[11]
		Silicon Oil	$1.02*10^{-3}$ kg	Ecoinvent v 3.8[12]
	Equipment/plants	Round-bottom two neck flask	$2.43*10^{-4}$ p	Modelled from Ecoinvent v 3.8 database sub process as detailed in Table S21[13]
		Magnetic stirrer	$1.4*10^{-4}$ p	Modelled from Ecoinvent v 3.8 database sub process as detailed in Table S15[14]
		Magnetic stirr bar	$7*10^{-5}$ p	Modelled from Ecoinvent v 3.8 database sub process as detailed in Table S14[15]
		Silicone oil container	$1.22*10^{-4}$ p	Modelled from Ecoinvent v 3.8 database sub process as detailed in Table S13[16]
		Aspiration system	$1*10^{-4}$ p	Modelled from Ecoinvent v 3.8 database sub process as detailed in Table S8[17]
		Activated carbon air filter	$4*10^{-4}$ p	Modelled from Ecoinvent v 3.8 database sub process as detailed in Table S9[18]
	Transports	Transport of raw materials	70.44 kgkm	Ecoinvent v3.8[19]
		Transport of small equipment	0.15 kgkm	Ecoinvent v 3.8[20]
		Transport of large equipment/plants	0.0167 tkm	Ecoinvent v 3.8[21]
	Energy	Electric Energy	3500 Wh	Ecoinvent v 3.8[22]
		Electric Energy	131.05 kJ	Ecoinvent v 3.8[23]
		Electric Energy	16.52 kJ	Ecoinvent v 3.8[24]
		Electric Energy	0.67 kWh	Ecoinvent v 3.8[25]
Output	Avoided Products	Diethyl ether	33.64 g	Ecoinvent v 3.8[26]
		Toluene	6.96 g	Ecoinvent v 3.8[27]
		Ethyl acetate	59.96 g	Ecoinvent v 3.8[28]
		Hexane	219.77 g	Ecoinvent v 3.8[29]
	Emission to air	Toluene	$1.41*10^{-5}$ g	SimaPro airborne emission substance lis[30]
		1,3-Propanediol	$7.05*10^{-9}$ g	SimaPro airborne emission substance list[31]
		Diethyl ether	$8.78*10^{-4}$ g	SimaPro airborne emission substance list[32]

	Hexane	$2.35*10^{-3}$ g	SimaPro airborne emission substance list[33]
	Ethyl acetate	$3.59*10^{-4}$ g	SimaPro airborne emission substance list[34]
	Water	$5.53*10^{-5}$ g	SimaPro airborne emission substance list[35]
End of Life	Spent Solvent Mixture	384.03 g	Ecoinvent v 3.8[36]
	Spent Solvent Mixture	$1.02*10^{-3}$ kg	Ecoinvent v 3.8[37]
	Waste treatment	85 g	Ecoinvent v 3.8[38]

[1]The Ecoinvent process used was: Propylene glycol, liquid {RER}| production | APOS, U.
[2]Toluene, liquid {RER}| production | APOS, U.
[3]Sodium carbonate was used during the work up procedure but this material wasn't available on the Ecoinvent database, so Potassium Carbonate was used. The Ecoinvent process used was: Potassium carbonate {GLO}| production, from potassium hydroxide | APOS, U.
[4]Water necessary for the preparation of the solution of diethyl ether. The Ecoinvent process used was: Water, deionised {Europe without Switzerland} | water production, deionised | APOS, U.
[5]Diethyl ether used during thw workup procedure for the extraction of the organic phase. The Ecoinvent process used was: Diethyl ether, without water, in 99.95% solution state {RoW}| ethylene hydration | APOS, U.
[6]Water used for the washing of the organic phase, after the extraction with diethyl ether, in order to remove the residual catalyst. The Ecoinvent process used was: Water, deionised {Europe without Switzerland} | water production, deionised | APOS, U.
[7]Sodium chloride used to wash the organic phase to remove unreacted reagents. The Ecoinvent process used was: Sodium chloride, brine solution {RER}| production | APOS, U.
[8]Sodium sulphate anhydrous used to remove the residual water in the organic phase. The Ecoinvent process used was: Sodium sulphate, anhydrite {RER}| Mannheim process | APOS, U.
[9]Silica used for the column chromatography to purify the residue. The Ecoinvent process used was: Activated silica {GLO}| production | APOS, U.
[10]Hexane used as liquid phase during the column chromatography together with ethyl acetate (n-hexane/EtOAc 5:1). The Ecoinvent process used was: Hexane {RER}| molecular sieve separation of naphtha | APOS, U.
[11]Ethyl acetate used as liquid phase during the column chromatography with hexane (n-hexane/EtOAc 5:1). The Ecoinvent process used was: Ethyl acetate {RER}| production | APOS, U.
[12]The Ecoinvent process used was: Silicone product {RER}| production | APOS, U.
[13]The reported value was calculated by the following formula: 1/28800h*7h, where 28800 h is the lifetime of the two neck round bottomed flask and 7h is its time of usage.
[14]The reported value was calculated by the following formula: 1/50000h*7h, where 50000 h is the lifetime of the magnetic stirrer and 7h is the time of usage of the magnetic stirrer.
[15]The reported value was calculated by the following formula: 1/100000h*7h, where 100000 h is the lifetime of the stir bar and 7h is its time of usage.
[16]The reported value was calculated by the following formula: 1/57600h*7h, where 57600 h is the lifetime of the silicone oil container and 7h is its time of usage.
[17]The reported value was calculated by the following formula: 1/79750h*8h, where 79750 h is the lifetime of the aspiration system and 8h is its time of usage. One additional hour of functioning was considered for the work-up procedure.
[18]The reported value was calculated by the following formula: 1/20000h*8h, where 20000 h is the lifetime of the activated carbon air filter and 8h is its time of usage. One additional hour of functioning was considered for the work-up procedure.

[19]Transport of raw materials. The Ecoinvent process used was: Transport, freight, lorry 3.5-7.5 metric ton, EURO6 {RER}| transport, freight, lorry 3.5-7.5 metric ton, EURO6 | APOS, U. An average distance of 100 km was considered.
[20]Transport of small equipment. The Ecoinvent process used was: Transport, freight, lorry 3.5-7.5 metric ton, EURO6 {RER}| transport, freight, lorry 3.5-7.5 metric ton, EURO6 | APOS, U. an average distance of 100 km was considered.
[21]Transport for large equipment. The Ecoinvent process used was: Transport, freight, lorry 16-32 metric ton, euro6 {RER}| market for transport, freight, lorry 16-32 metric ton, EURO6 | APOS, U. an average distance of 100 km was considered.
[22]Electric energy necessary to heat the reaction mixture at 140°C. It was calculated as power*time, considering a power of 500W and a reaction time of seven hours. The Ecoinvent process used was: Electricity, low voltage {IT}| electricity voltage transformation from medium to low voltage | APOS, U.
[23]Electric energy necessary to evaporate n-hexane and ethyl acetate, calculated using the vaporization enthalpy for each substance, which are respectively 32 kJ/mol and 34 kJ/mol. The Ecoinvent process used was: Electricity, low voltage {IT}| electricity voltage transformation from medium to low voltage | APOS, U.
[24]Electric energy necessary to evaporate diethyl ether and toluene, calculated using the vaporization enthalpy for each substance which are respectively 27 kJ/mol and 38 kJ/mol. The Ecoinvent process used was: Electricity, low voltage {IT}| electricity voltage transformation from medium to low voltage | APOS, U.
[25]Electric energy necessary to the use of the aspiration system for the whole synthesis time, i.e. 8 h. The power was calculated by considering the air flow rate of 250 m^3/h, a total load loss of 110.8076 kg/m^2, and an efficiency of 90%. The Ecoinvent process used was: Electricity, low voltage {IT}| electricity voltage transformation from medium to low voltage | APOS, U.
[26]Avoided Products, it is assumed that 95% of Diethyl ether can be recovered. The Ecoinvent process used was: Diethyl ether, without water, in 99.95% solution state {RoW}| ethylene hydration | APOS, U.
[27]Avoided Products, it is assumed that 80% of Toluene can be recovered. The Ecoinvent process used was: Toluene, liquid {RER}| production | APOS, U.
[28]Avoided Products, it is assumed that 80% of Ethyl Acetate can be recovered. The Ecoinvent process used was: Ethyl acetate {RER}| production | APOS, U.
[29]Avoided Products, it is assumed that 80% of Hexane can be recovered. The Ecoinvet process used was: Hexane {RER}| molecular sieve separation of naphtha | APOS, U.
[30]Amount of toluene released into the atmosphere, as calculated by the formula reported in equation 1 of the main manuscript.
[31]Amount of 1,3-Propanediol released into the atmosphere, as calculated by the formula reported in equation 1 of the main manuscript.
[32]Amount of Diethyl ether released into the atmosphere, as calculated by the formula reported in equation 1 of the main manuscript.
[33]Amount of Hexane released into the atmosphere, as calculated by the formula reported in equation 1 of the main manuscript.
[34]Amount of Ethyl Acetate released into the atmosphere, as calculated by the formula reported in equation 1 of the main manuscript.
[35]Amount of Water released into the atmosphere, as calculated by the formula reported in equation 1 of the main manuscript.
[36]End of life treatment for what used during the synthesis, work up procedure and isolation of the final product. The Ecoinvent process used was: Spent solvent mixture {Europe without Switzerland}| treatment of spent solvent mixture, hazardous waste incineration, with recovery energy | APOS, U.
[37]End of life treatment for the silicon oil The Ecoinvent process used was: Spent solvent mixture {Europe without Switzerland}| treatment of spent solvent mixture, hazardous waste incineration, with recovery energy | APOS, U.
[38]End of life chosen for the Activated Silica used for the column chromatography, which is not recoverable. The Ecoinvent process used was: Hazardous waste, for incineration {Europe without Switzerland}| treatment of hazardous waste, hazardous waste incineration, with energy recovery | APOS, U.

Table S41. Endpoint damage assessment results (ReCiPe 2016, H/A) associated with the production of 1g of the five bioplasticizers **4a-e**.

Damage category	Unit	Plasticizer **4a**	Plasticizer **4b**	Plasticizer **4c**	Plasticizer **4d**	Plasticizer **4e**
Human health	DALY	6.19E-05	4.18E-05	8.77E-05	8.02E-05	7.83E-05
Ecosystems	species.yr	1.30E-07	8.82E-08	1.85E-07	1.69E-07	1.65E-07
Resources	USD2013	2.29E+00	1.54E+00	3.24E+00	2.6E+00	2.89E+00

Table S42. Detailed single score results for the synthesis of 1 g of each of the five bioplasticizers **4a-e**.

Damage category	Unit	Plasticizer **4a**	Plasticizer **4b**	Plasticizer **4c**	Plasticizer **4d**	Plasticizer **4e**
Total	Pt	1.08E+00	7.32E-01	1.54E+00	1.41E+00	1.37E+00
Human health	Pt	1.03E+00	6.98E-01	1.46E+00	1.34E+00	1.31E+00
Ecosystems	Pt	3.53E-02	2.39E-02	5.00E-02	4.57E-02	4.46E-02
Resources	Pt	1.63E-02	1.10E-02	2.31E-02	2.12E-02	2.06E-02

Table S43. Detailed single score (ReCiPe 2016 H/A) results for the synthesis of 1 g of bioplasticizer **4b**.

Damage category	Human health	Ecosystems	Resources
Unit	Pt	Pt	Pt
Total	6.98E-01	2.39E-02	1.10E-02
Plasticizer 4b	9.83E-08	9.95E-08	0.00E+00
Precursor 3b	3.08E-01	1.05E-02	4.91E-03
Propylene glycol	1.18E-04	4.71E-06	2.98E-06
Toluene	1.14E-03	4.90E-05	1.19E-04
P-toluenesulfonic acid	3.01E-05	1.06E-06	7.80E-07
Na_2CO_3	4.63E-03	1.33E-04	5.08E-05
H_2O	1.31E-05	9.20E-07	4.73E-08
Diethyl ether	1.61E-02	6.01E-04	1.04E-03
H_2O	2.18E-05	1.53E-06	7.88E-08
Brine solution	1.34E-03	3.03E-05	8.63E-06
Na_2SO_4	1.28E-03	3.32E-05	1.04E-05
Silica gel	3.26E-02	8.38E-04	2.24E-04
Hexane	2.26E-02	9.37E-04	2.61E-03
Ethyl acetate	2.45E-02	8.87E-04	1.04E-03
Transport of reagents	3.89E-03	1.37E-04	1.26E-04
Round bottomed flask	1.39E-05	4.51E-07	1.46E-07
Heating plate	4.58E-04	6.72E-06	3.79E-06
Stir bar	4.27E-07	4.29E-09	1.39E-09
Aspiration system	4.05E-02	6.08E-04	1.40E-04
Active carbon filter	3.46E-02	9.30E-04	1.95E-04
Transport of aspiration system and filter	2.78E-04	1.08E-05	9.97E-06
Transport small equipment	8.53E-06	3.00E-07	2.76E-07
Silicone oil container	3.11E-04	2.66E-06	9.41E-07
Silicone oil	3.80E-04	1.37E-05	8.63E-06
Energy to recover ether and toluene	2.09E-04	7.95E-06	4.46E-06
Energy to recovery hexane and ethylacetate	1.66E-03	6.30E-05	3.54E-05
Energy for aspiration system	3.05E-02	1.16E-03	6.52E-04
Energy for heating for 7h	1.59E-01	6.06E-03	3.40E-03
Diethyl ether recovered	-1.52E-02	-5.69E-04	-9.83E-04
Toluene recovered	-9.12E-04	-3.92E-05	-9.53E-05
Ethyl acetate recovered	-1.96E-02	-7.09E-04	-8.30E-04
Hexane recovered	-1.80E-02	-7.48E-04	-2.09E-03
Waste treatment of reaction waste	4.69E-02	2.26E-03	2.24E-04
Waste treatment silica gel	1.99E-02	6.76E-04	1.97E-04
Waste treatment silicone oil	1.24E-04	5.98E-06	5.95E-07

Table S44. Detailed single score results (ReCiPe 2016 H/A) associated to the production of 1.1314 g of the diester precursor **3b**.

Damage category	Human health	Ecosystems	Resources
Unit	Pt	Pt	Pt
Total	3.08E-01	1.05E-02	4.91E-03
Precursor 3b	2.65E-09	8.26E-09	0.00E+00
Precursor 2b	1.51E-01	5.16E-03	2.46E-03
Levulinic acid	6.76E-02	2.21E-03	1.09E-03
Toluene	6.44E-04	2.77E-05	6.73E-05
H_2SO_4	2.61E-06	5.75E-08	1.26E-08
Na_2CO_3	1.31E-03	3.76E-05	1.44E-05
H_2O	3.70E-06	2.60E-07	1.34E-08
Diethyl ether	4.54E-03	1.70E-04	2.93E-04
H_2O	6.17E-06	4.33E-07	2.23E-08
Brine	3.80E-04	8.56E-06	2.44E-06
Na_2SO_4	3.61E-04	9.37E-06	2.94E-06
Transport of	5.68E-04	2.00E-05	1.84E-05
Round bottomed	3.93E-06	1.27E-07	4.12E-08
Stir bar	1.21E-07	1.21E-09	3.92E-10
Heating plate	1.29E-04	1.90E-06	1.07E-06
Activated carbon	9.76E-03	2.63E-04	5.50E-05
Aspiration system	1.14E-02	1.72E-04	3.96E-05
Transport of	2.41E-06	8.49E-08	7.81E-08
Transport of	7.86E-05	3.05E-06	2.82E-06
Silicone oil	8.78E-05	7.51E-07	2.66E-07
Silicone oil	1.07E-04	3.86E-06	2.44E-06
Energy to recover	7.18E-05	2.73E-06	1.53E-06
Energy for	8.62E-03	3.28E-04	1.84E-04
Energy for	4.50E-02	1.71E-03	9.62E-04
Diethyl ether	-4.30E-03	-1.61E-04	-2.78E-04
Toluene	-5.15E-04	-2.21E-05	-5.38E-05
Waste treatment	1.09E-02	5.24E-04	5.21E-05
Waste treatment	3.51E-05	1.69E-06	1.68E-07

Table S45. Detailed single score results (ReCiPe 2016 H/A) associated to the production of 1.0166 g of levulinic acid from exploded *Cynara cardunculus* L. residual biomass.

Damage category	Human health	Ecosystems	Resources
Unit	Pt	Pt	Pt
Total	6.76E-02	2.21E-03	1.09E-03
Exploded biomass	1.33E-04	9.72E-06	1.61E-06
HCl	5.20E-07	1.80E-08	2.40E-08
H_2O	6.39E-08	4.48E-09	2.30E-10
2Me-THF	2.11E-02	7.36E-04	4.33E-04
Transport reagents	1.06E-05	3.73E-07	3.44E-07
Aspiration system	8.37E-03	1.26E-04	2.90E-05
Active carbon filter	7.15E-03	1.92E-04	4.02E-05
Transpor aspiration system and filter	5.76E-05	2.23E-06	2.06E-06
MW reactor	4.31E-05	6.11E-07	2.86E-07
Energy for microwave treatment	1.09E-03	4.17E-05	2.34E-05
Round bottomed flask	2.51E-06	8.14E-08	2.63E-08
Heating plate	4.78E-04	7.01E-06	3.95E-06
Stir bar	7.12E-08	7.15E-10	2.31E-10
Energy for stirring	1.59E-04	6.07E-06	3.41E-06
Vigreux column	1.16E-06	3.76E-08	1.21E-08
Silicone oil container	1.30E-05	1.11E-07	3.92E-08
Silicone oil	1.59E-03	5.70E-05	3.60E-05
Energy for heating during distillation	6.64E-03	2.53E-04	1.42E-04
Vacuum pump	1.95E-03	3.16E-05	1.26E-05
Energy for vacuum pump	9.96E-03	3.79E-04	2.13E-04
Energy for aspiration system	6.31E-03	2.40E-04	1.35E-04
Transport small equipment	2.79E-05	9.85E-07	9.06E-07
Transport small equipment	3.64E-08	1.28E-09	1.18E-09
Waste treatment of reaction wastes	2.02E-03	9.74E-05	9.69E-06
Waste treatment of silicone oil	5.19E-04	2.49E-05	2.48E-06

Table S46. Detailed single score results (ReCiPe 2016 H/A) associated to the production of 5.008 g (i.e., the amount needed to obtain 1.0166 g of levulinic acid) of exploded *Cynara cardunculus* L. biomass.

Damage category	Human health	Ecosystems	Resources
Unit	Pt	Pt	Pt
Total	1.33E-04	9.72E-06	1.61E-06
Exploded biomass	0.00E+00	1.14E-16	0.00E+00
Epigean residue	3.87E-06	4.27E-06	2.95E-08
H_2SO_4	2.74E-06	6.03E-08	1.32E-08
H_2O impregnation	3.03E-07	2.48E-08	6.59E-10
H_2O washing	4.10E-07	3.35E-08	8.91E-10
Transport biomass	3.21E-06	1.13E-07	1.04E-07
Transport H_2SO_4	1.51E-07	5.30E-09	4.88E-09
Chipping of biomass	7.08E-07	2.19E-08	5.93E-09
Tank with filter for impregnation	3.29E-07	2.73E-09	1.01E-09
Steam explosion tank	1.08E-06	1.01E-08	3.74E-09
Energy for steam explosion	2.59E-05	9.85E-07	5.53E-07
Steam	1.66E-05	6.14E-07	4.20E-07
Settling tank	1.41E-08	1.16E-10	4.32E-11
Press	2.35E-09	2.91E-11	1.04E-11
Energy for first pressing	3.26E-07	1.24E-08	6.98E-09
Energy to heat water at 50°C	3.19E-06	1.22E-07	6.83E-08
Press	9.50E-09	1.18E-10	4.22E-11
Energy for second pressing	1.32E-06	5.03E-08	2.82E-08
Storage tank	2.88E-09	2.37E-11	8.82E-12
Transport impregnation tank and filter	1.37E-08	5.32E-10	4.92E-10
Transport steam explosion reactor	4.39E-10	1.70E-11	1.57E-11
Transport of press	5.24E-12	2.03E-13	1.88E-13
Aspiration system	2.00E-06	3.01E-08	6.94E-09
Activated carbon filter	1.71E-06	4.61E-08	9.63E-09
Energy for aspiration system	1.51E-06	5.75E-08	3.23E-08
Transport aspiration system and active carbon filter	1.38E-08	5.34E-10	4.94E-10
H_2SO_4 recovered	-1.92E-06	-4.22E-08	-9.27E-09
H_2O recovered	-2.12E-07	-1.74E-08	-4.61E-10
Waste treatment of H_2SO_4 containing waste	6.95E-05	3.33E-06	3.27E-07
Waste treatment of waste from second pressing	5.35E-08	-1.05E-08	1.12E-09

Table S47. Detailed single score results (ReCiPe 2016 H/A) associated to the production of 1 t of *Cynara cardunculus* L. epigean residue.

Damage category	Unit	Total	Epigean residue	Seeds	Deep tillage	Surface tillage	Harrowing	Sowing	Nitrogen fertiliser	Phosporus fertiliser	Compost	Fertilising	Bag	Seeds harvesting	Biomass harvesting	Transport	Nitrogen fertiliser deriving from hypogeum	Phosporus fertiliser deriving from hypogeum	Potassium fertiliser deriving from hypogeum
Human health	Pt	1.93E+00	1.82E-01	5.50E-04	2.11E-02	3.14E-02	7.33E-03	6.75E-03	2.85E-01	2.85E-01	1.06E+00	6.90E-03	1.74E-02	8.82E-02	3.14E-03	5.10E-03	-2.95E-02	-3.49E-02	-2.52E-03
Ecosystems	Pt	2.13E+00	2.01E+00	6.07E-04	5.25E-04	8.90E-04	2.13E-04	1.98E-04	7.42E-03	7.42E-03	9.81E-02	2.00E-04	6.03E-04	2.35E-03	9.26E-05	1.94E-04	-9.34E-04	-9.08E-04	-7.73E-05
Resources	Pt	1.47E-02	0.00E+00	4.19E-06	3.18E-04	5.23E-04	1.01E-04	9.26E-05	2.06E-03	2.06E-03	8.47E-03	1.12E-04	5.65E-04	9.07E-04	4.48E-05	1.73E-04	-3.92E-04	-2.52E-04	-5.35E-05

Table S48. Detailed midpoint results (ReCiPe 2016) of the sensitivity analysis referred to the synthesis of 1g of bioplasticizer 4b, in accordance with the three scenarios considered alternatively to the reference one.

Impact category	Unit	Reference scenario	Scenario 1: Swedish mix	Scenario 2: +30% Yield	Scenario 3: 95% solvent recovery
Global warming	kg CO_2 eq	2.04E+01	1.00E+01	1.28E+01	1.98E+01
Stratospheric ozone depletion	kg CFC11 eq	1.22E-05	6.12E-06	7.63E-06	1.19E-05
Ionizing radiation	kBq Co-60 eq	1.73E+00	9.20E+00	1.08E+00	1.71E+00
Ozone formation, Human health	kg NOx eq	3.24E-02	1.57E-02	2.03E-02	3.16E-02
Fine particulate matter formation	kg PM2.5 eq	2.22E-02	1.16E-02	1.39E-02	2.19E-02
Ozone formation, Terrestrial ecosystems	kg NOx eq	3.32E-02	1.63E-02	2.08E-02	3.23E-02
Terrestrial acidification	kg SO2 eq	6.16E-02	2.91E-02	3.86E-02	6.07E-02
Freshwater eutrophication	kg P eq	7.31E-03	4.35E-03	4.60E-03	7.01E-03
Marine eutrophication	kg N eq	5.47E-04	4.00E-04	3.43E-04	5.37E-04
Terrestrial ecotoxicity	kg 1,4-DCB	7.24E+01	6.95E+01	4.50E+01	7.15E+01
Freshwater ecotoxicity	kg 1,4-DCB	7.73E-01	6.45E-01	4.83E-01	7.64E-01
Marine ecotoxicity	kg 1,4-DCB	1.02E+00	8.47E-01	6.39E-01	1.01E+00
Human carcinogenic toxicity	kg 1,4-DCB	1.41E+00	1.23E+00	8.72E-01	1.40E+00
Human non-carcinogenic toxicity	kg 1,4-DCB	1.59E+01	1.21E+01	9.94E+00	1.57E+01
Land use	m^2a crop eq	5.98E-01	7.39E-01	3.75E-01	5.88E-01
Mineral resource scarcity	kg Cu eq	8.59E-02	9.26E-02	4.53E-02	8.51E-02
Fossil resource scarcity	kg oil eq	5.11E+00	1.77E+00	3.20E+00	4.91E+00
Water consumption	m^3	3.29E-01	2.27E-01	2.06E-01	3.26E-01

Table S49. Reductions in glass transition temperature (T_g), melting temperature (T_m) and storage modulus (E') induced by the bioplasticizers **4a-e** when added at 10 or 20 phr to 250 mg of PHB, and their relative single score environmental impacts.

		Tg (°C)		Tm (°C)		E' (MPa)	
Entry code	Plasticizer content (phr)	ΔTg	Pt	ΔTm	Pt	ΔE'	Pt
**4a-10**	10	12	2.71E-02	2.8	2.71E-02	407	2.71E-02
**4a-20**	20	16.3	5.42E-02	4.8	5.42E-02	1021	5.42E-02
**4b-10**	10	4	1.83E-02	2.8	1.83E-02	935	1.83E-02
**4b-20**	20	6.2	3.66E-02	4.7	3.66E-02	1200	3.66E-02
**4c-10**	10	5	3.84E-02	0.8	3.84E-02	541	3.84E-02
**4c-20**	20	9.3	7.68E-02	6.4	7.68E-02	1249	7.68E-02
**4d-10**	10	7.7	3.51E-02	1.8	3.51E-02	615	3.51E-02
**4d-20**	20	16.8	7.03E-02	8.2	7.03E-02	1432	7.03E-02
**4e-10**	10	9.6	3.43E-02	1.3	3.43E-02	494	3.43E-02
**4e-20**	20	16.5	6.85E-02	9.2	6.85E-02	1449	6.85E-02

FIGURES

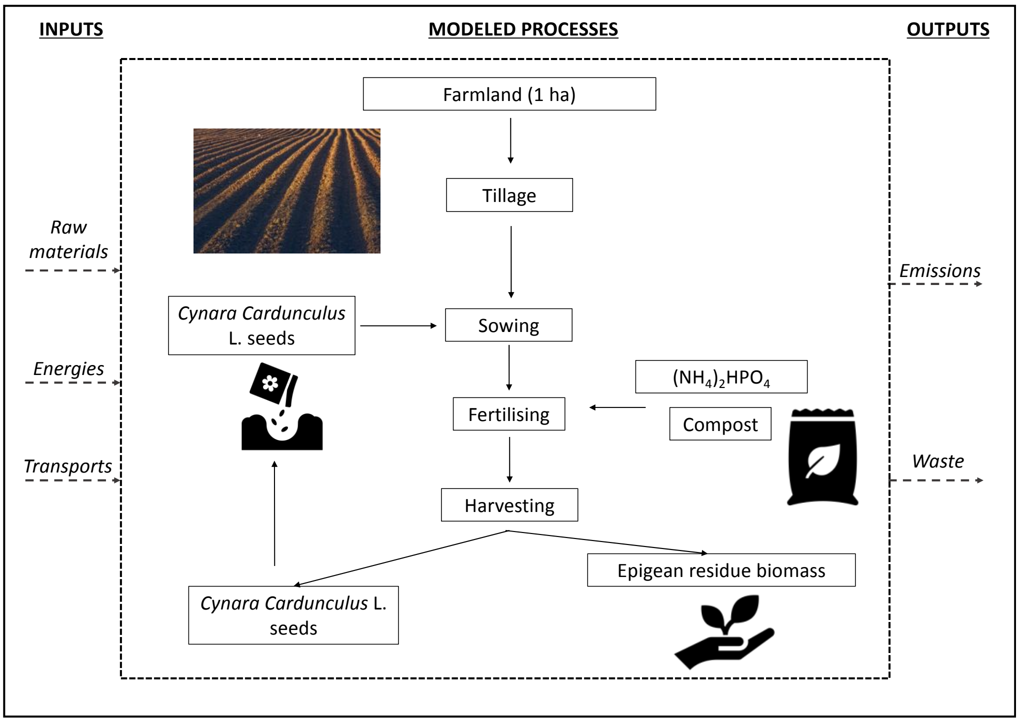

Figure S1. Flowchart showing the system boundaries considered in the LCA of *Cynara Cardunculus* L. crop production.

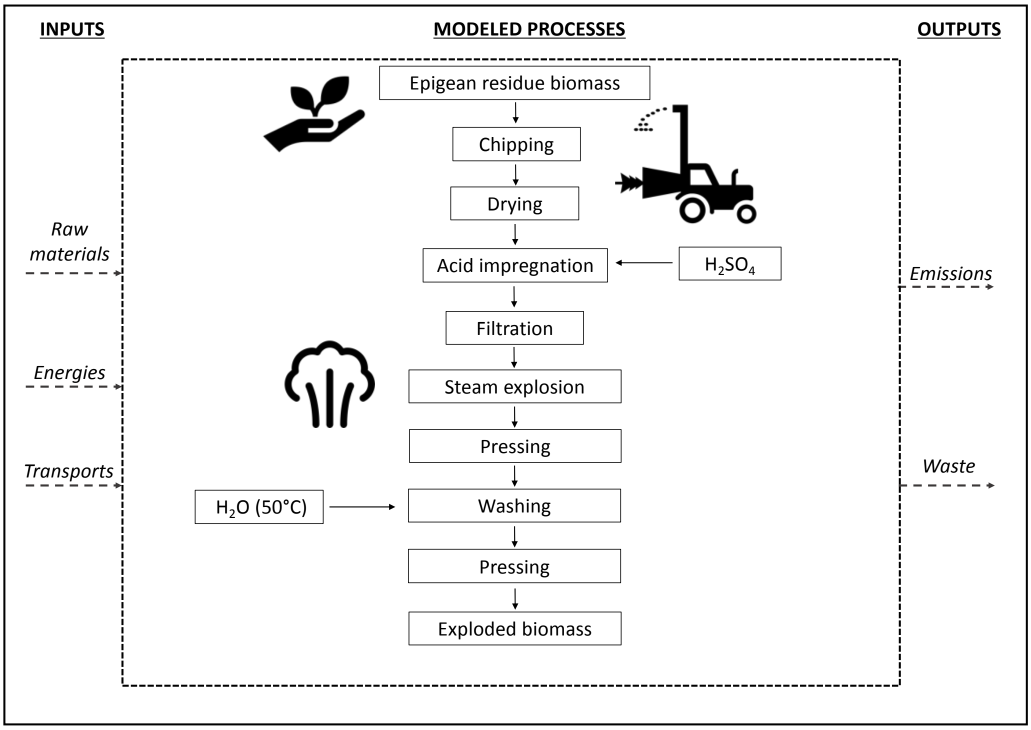

Figure S2. Flowchart showing the system boundaries considered in the LCA of residual *Cynara Cardunculus* L. biomass pre-treatment/steam explosion process.

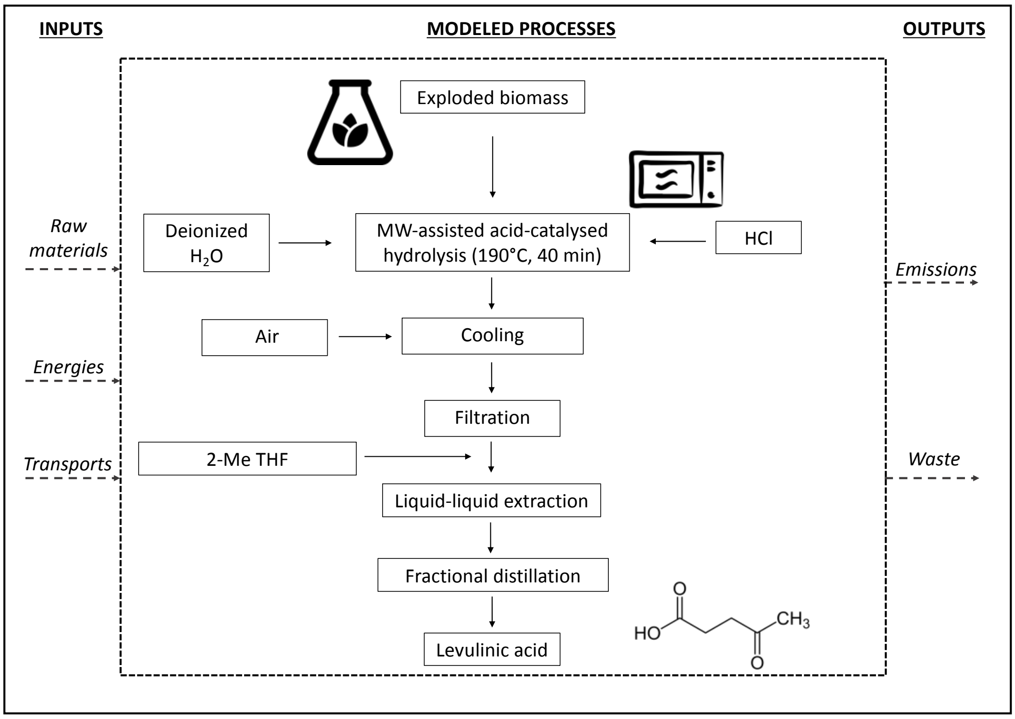

Figure S3. Flowchart showing the system boundaries considered in the LCA of the obtainment of levulinic acid from exploded residual *Cynara Cardunculus* L. biomass.

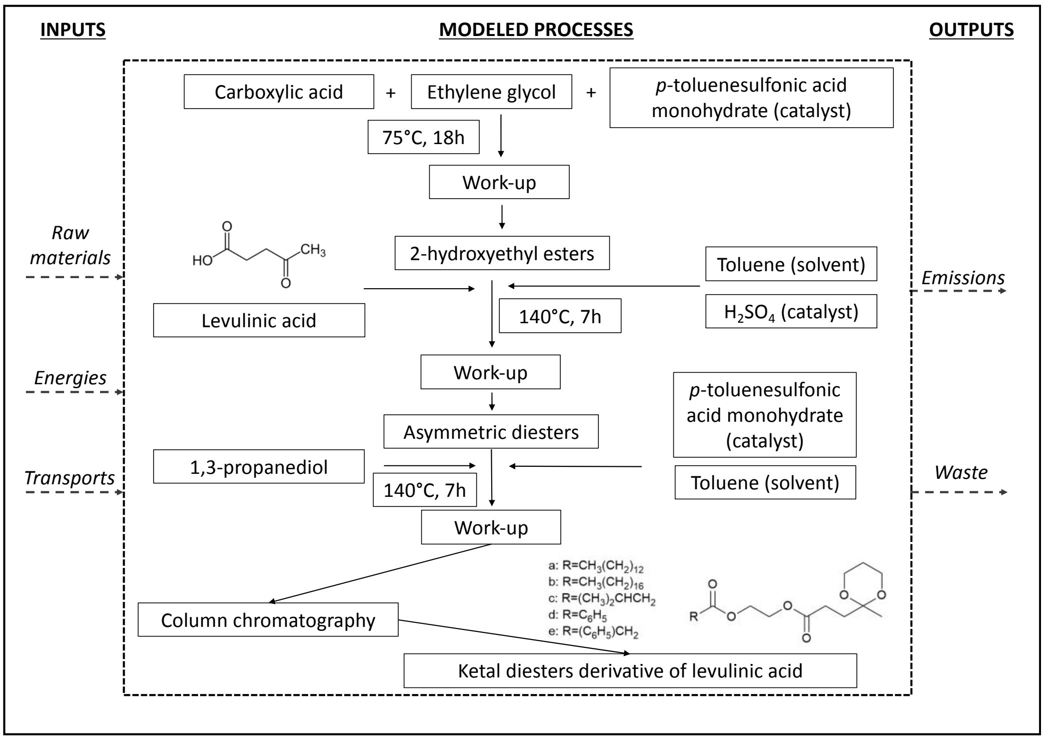

Figure S4. Flowchart showing the system boundaries considered in the LCA of the synthesis of five ketal diester derivatives of levulinic acid.

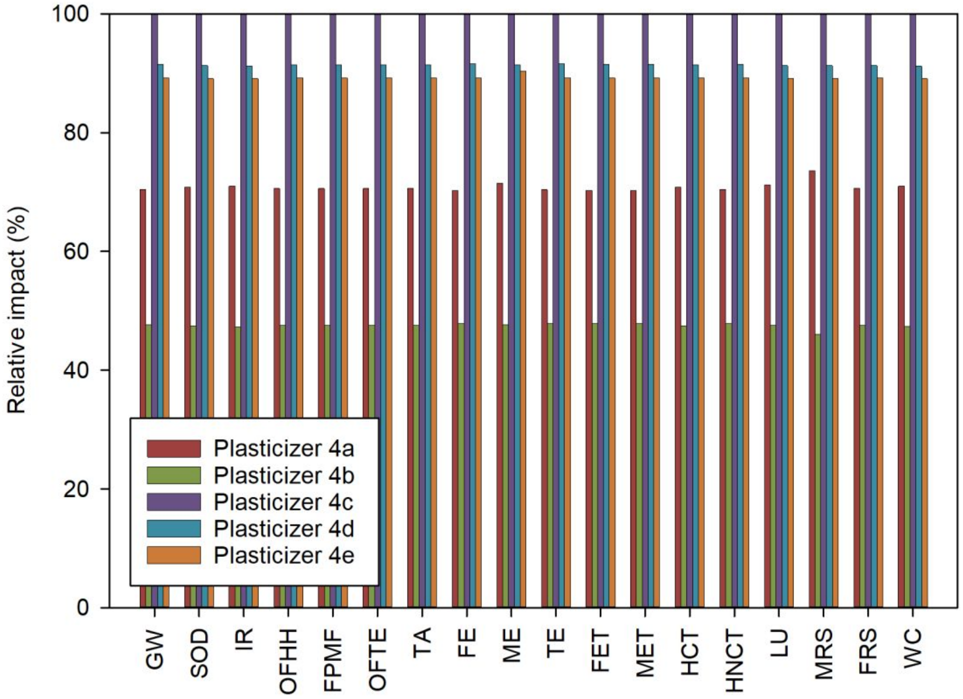

Figure S5. Relative environmental impacts, calculated at a midpoint level (ReCiPe 2016, H) associated to the preparation of 1 g of the five bioplasticizers **4a-e** (**Scheme 1** of the main manuscript). The impact categories considered by the impact assessment method are: Global warming (GW, kg CO_2 eq), stratospheric ozone depletion (SOD, kg CFC-11 eq), ionizing radiation (IR, kBq Co-60 eq), ozone formation-human health (OFHH, kg NO_x eq), fine particulate matter formation (FPMF, kg PM2.5 eq), ozone formation-terrestrial ecosystems (OFTE, kg NO_x eq), terrestrial acidification (TA, kg SO_2 eq), freshwater eutrophication (FE, kg P eq), marine eutrophication (ME, kg N eq), terrestrial ecotoxicity (TE, kg 1,4-DCB), freshwater ecotoxicity (FET, kg 1,4-DCB), marine ecotoxicity (MET, kg 1,4-DCB), human carcinogenic toxicity (HCT, kg 1,4-DCB), human non-carcinogenic toxicity (HNCT, kg 1,4-DCB), land use (LU, m2a crop eq), mineral resource scarcity (MRS, kg Cu eq), fossil resource scarcity (FRS, kg oil eq), and water consumption (WC, m^3).

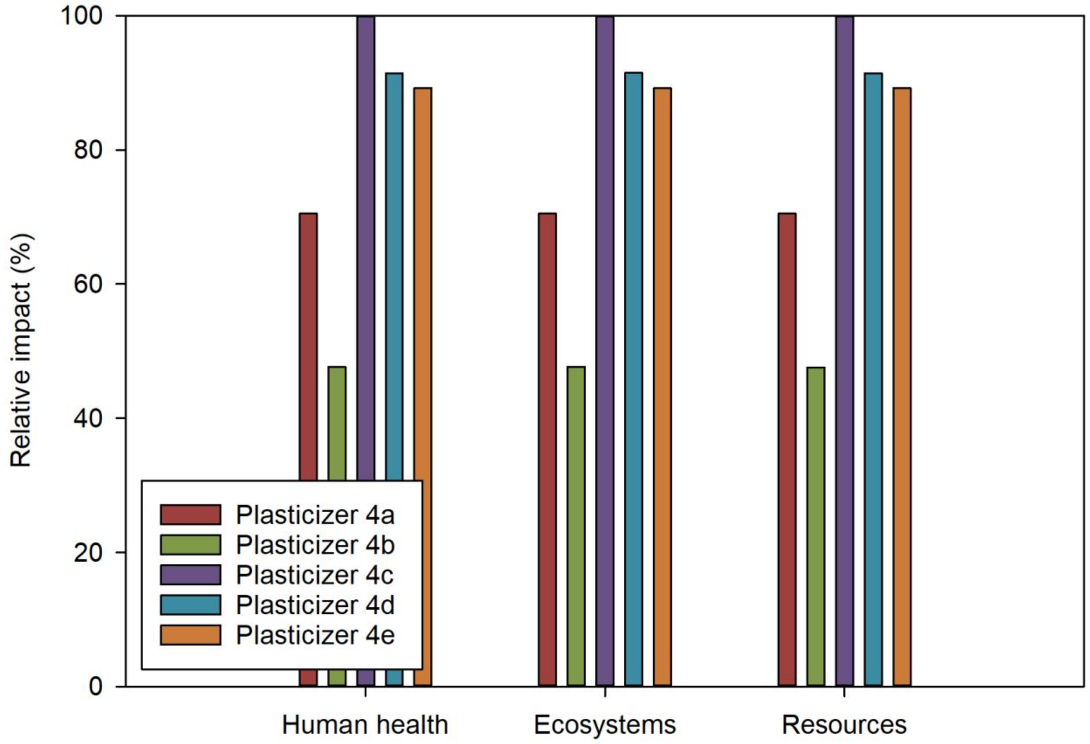

Figure S6. Relative impact (%) associated to the production of 1 g of the five bioplasticizers **4a-e**, calculated at the endpoint level, i.e., in terms of the damage categories Human health (DALY), Ecosystems (species·year) and Resources (USD2013).

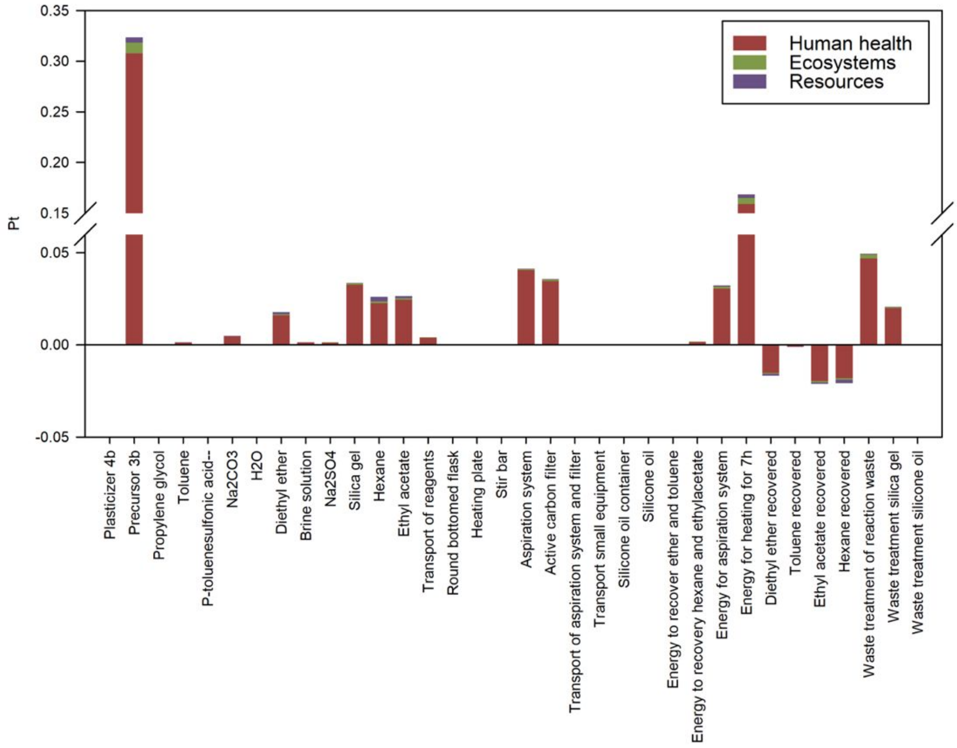

Figure S7. Single score results (ReCiPe 2016 H/A) associated to the production of 1 g of the bioplasticizer 4**b**.

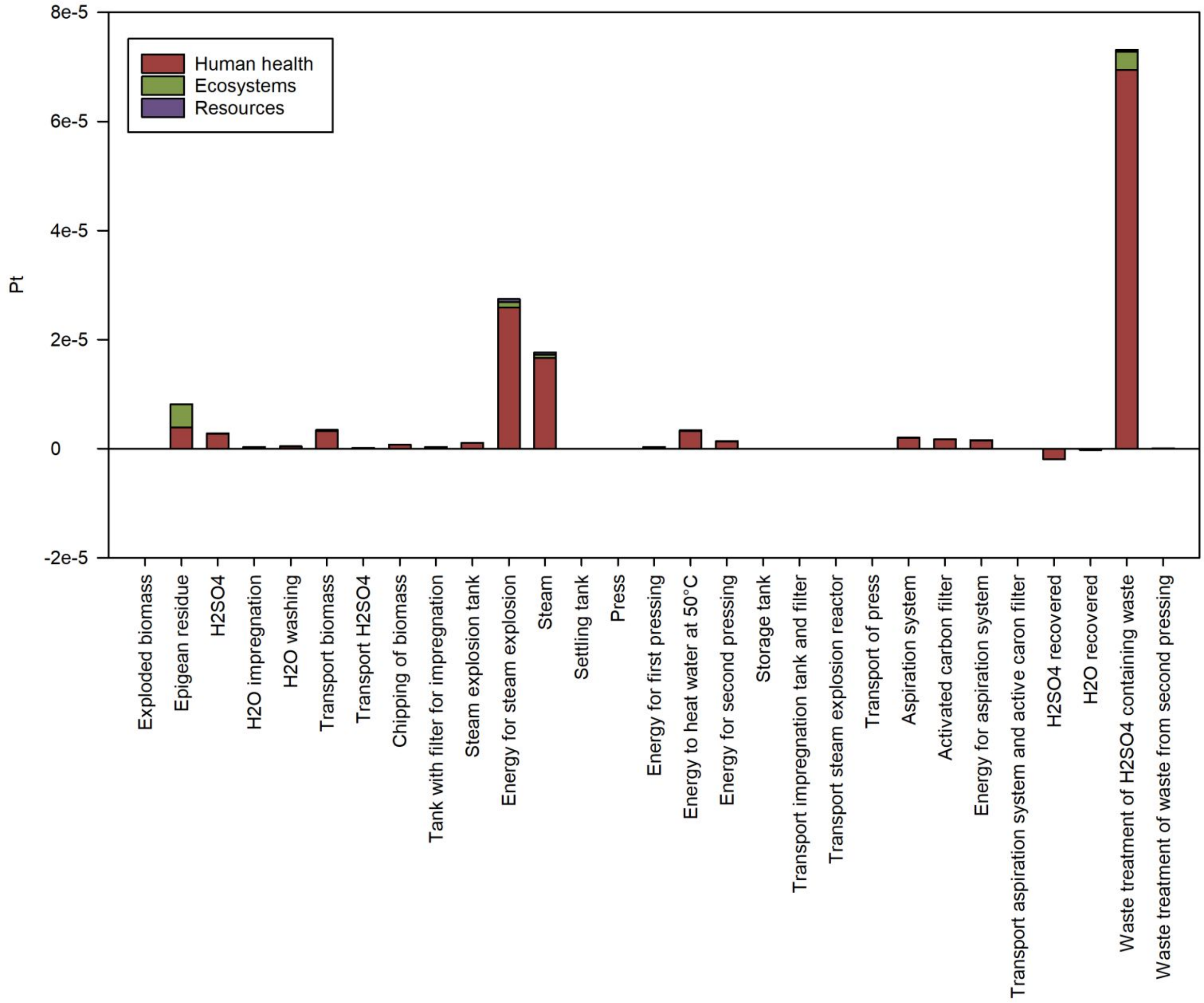

Figure S8. Single score results (ReCiPe 2016 H/A) associated to the production of 5.008 g (i.e., the amount needed to obtain 1.0166 g of levulinic acid) of exploded *Cynara cardunculus* L. biomass.

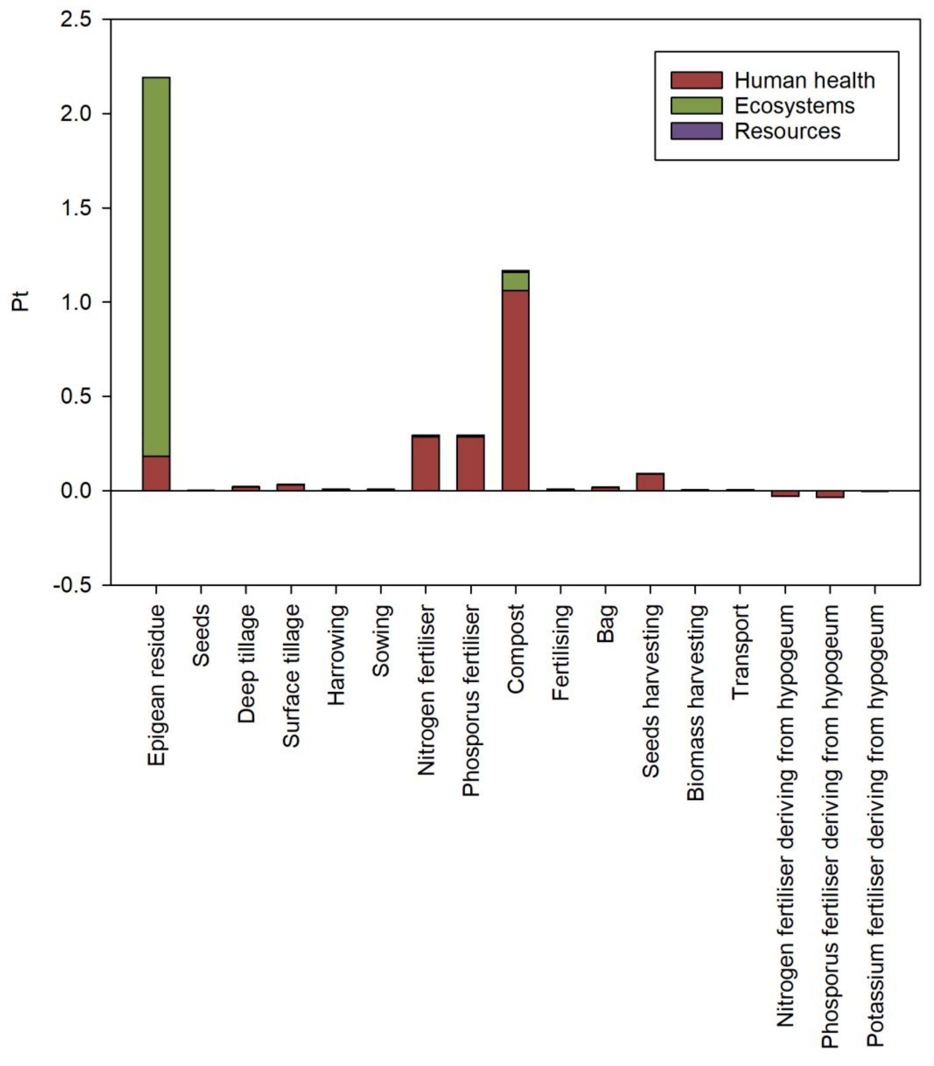

Figure S9. Single score results (ReCiPe 2016 H/A) associated to the production of 1 t of *Cynara cardunculus* L. epigean residue.

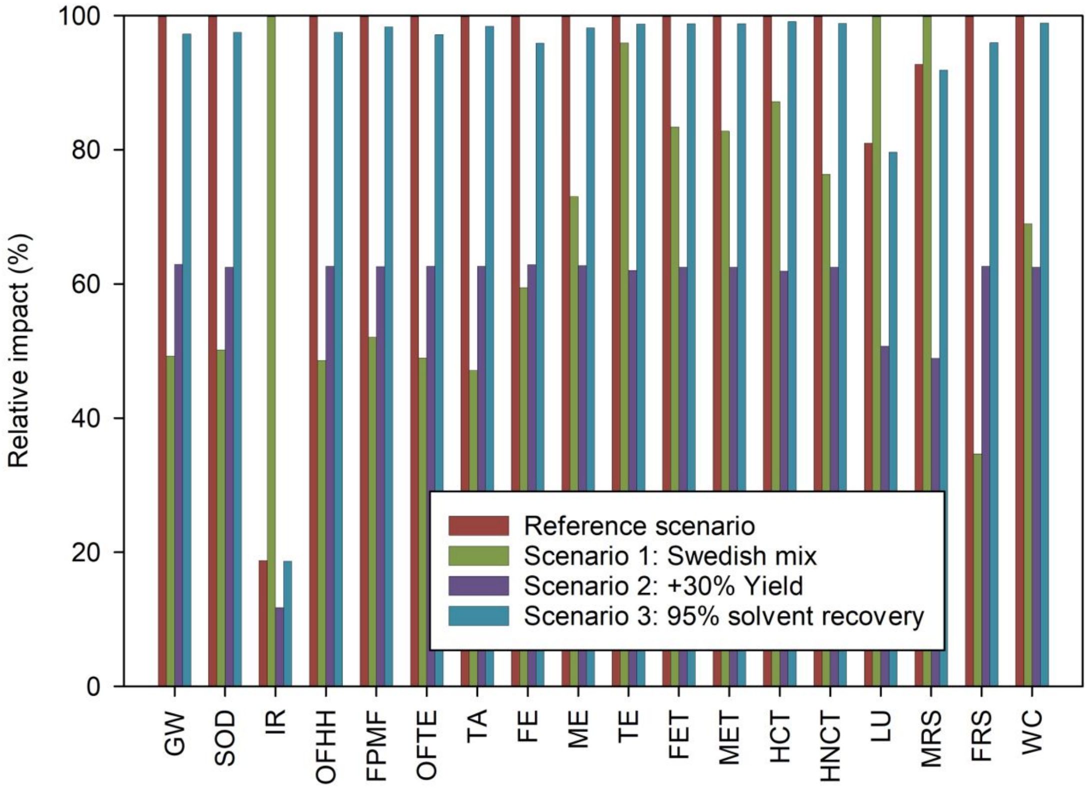

Figure S10. Relative environmental impacts, calculated at a midpoint level (ReCiPe 2016, H) associated to the preparation of 1 g of the bioplasticizer **4b** in accordance with the three scenarios considered alternatively to the reference one. The impact categories considered by the impact assessment method are: Global warming (GW, kg CO_2 eq), stratospheric ozone depletion (SOD, kg CFC-11 eq), ionizing radiation (IR, kBq Co-60 eq), ozone formation-human health (OFHH, kg NO_x eq), fine particulate matter formation (FPMF, kg PM2.5 eq), ozone formation-terrestrial ecosystems (OFTE, kg NO_x eq), terrestrial acidification (TA, kg SO_2 eq), freshwater eutrophication (FE, kg P eq), marine eutrophication (ME, kg N eq), terrestrial ecotoxicity (TE, kg 1,4-DCB), freshwater ecotoxicity (FET, kg 1,4-DCB), marine ecotoxicity (MET, kg 1,4-DCB), human carcinogenic toxicity (HCT, kg 1,4-DCB), human non-carcinogenic toxicity (HNCT, kg 1,4-DCB), land use (LU, m2a crop eq), mineral resource scarcity (MRS, kg Cu eq), fossil resource scarcity (FRS, kg oil eq), and water consumption (WC, m^3).

REFERENCES

[1] Nemecek, T.; Kägi, T. Life cycle inventories of agricultural production systems, Ecoinvent report n°15, December **2007**, pp. 27-40.

[2] Lupwayi, N. Z.; Clayton, G. W.; O'Donovan, J. T.; Harker, K. N.; Turkington, T. K.; Soon, Y. K. Nitrogen release during decomposition of crop residues under conventional and zero tillage. *Canadian J. Soil Sci.* **2006**, 86, 11-19.

[3] Peoples, M. B.; Herridge, D. F. Quantification of Biological Nitrogen Fixation in Agricultural Systems, in Pedrosa F. O.; Hungria, M.; Yates, G.; Newton, W. E. (eds.) Nitrogen Fixation: From Molecules to Crop Productivity. Kluwer AcademicPublisher, **2000**, pp. 519-524.

[4] Grimm, M.; Jones, R. J. A.; Rusco, E.; Montanarella, L. Soil Erosion Risk in Italy: a revised USLE approach. European Soil Bureau Research Report No.11, EUR 20677 EN, (**2002**), 28pp. Office for Official Publications of the European Communities, Luxembourg. https://esdac.jrc.ec.europa.eu/ESDB_Archive/pesera/pesera_cd/pdf/ita_er06.pdf

[5] Wolfensberger, U.; Dinkel, F. Beurteilung nachwachsender Rohstoffe in der Schweiz in den Jahren 1993-1996, FAT und Carbotech, im Auftrag des Bundesamtes für Landwirtschaft, Bern, **1997**.

[6] Elliott, D. C.; Frye, J. G. Hydrogenated 5-carbon compound and method of making, US Patent n° 5,883,266, March 16, **1999**.

[7] Khoo, H. H.; Wong, L. L.; Tan, J.; Isoni, V.; Sharrat, P. Synthesis of 2-methyl tetrahydrofuran from various lignocellulosic feedstocks: Sustainability assessment via LCA. *Res. Cons. Recycling* **2015**, 95, 174-182.

[8] Cespi, D.; Passarini, F.; Neri, E.; Vassura, I.; Ciacci, L.; Cavani, F. Life Cycle Assessment comparison of two ways for acrylonitrile production: the SOHIO process and an alternative route using propane. *J. Cleaner Prod.* **2014**, 69, 17-25.

[9] Gallego-Schmid, A.; Mendoza, J. M. F.; Azapagic, A. Environmental assessment of microwaves and the effect of European energy efficiency and waste management legislation. *Sci. Total Environ.* **2018**, 618, 487-499.

[10] Jinxing, W.; Wenhao, W.; Wenying, Z. Method for preparing p-toluenesulfonic acid by toluene sulfonation, CN Patent n° 101845004B, September 29, **2010**.

[11] Sinisi, A.; Degli Esposti, M.; Toselli, M.; Morselli, D.; Fabbri, P. Biobased ketal-diester additives derived from levulinic acid: synthesis and effect on the thermal stability and thermo-mechanical properties of Poly(vinyl chloride). *ACS Sustainable Chem. Eng.* **2019**, 7, 13920-13931.

[12] Gervajio, G. C. Fatty Acids and Derivates from Conconut Oil, Bailey's Industrial Oil and Fat Products, Sixth Edition, Six Volume Set. Edited by Freidoon Shahidi. John Wiley & Sons, Inc., **2005**.

[13] Anneken, D. J.; Both, S.; Christoph, R.; Fieg, G.; Steinberner, U.; Westfechtel, A. Fatty Acids, in Ullmann's Encyclopedia of Industrial Chemistry, Wiley-VCH, Weinheim , **2006**, Volume 14, pp. 73-116.
